# Coumarins in musculoskeletal health: a systematic review

**DOI:** 10.3389/fnut.2026.1737767

**Published:** 2026-09-09

**Authors:** Yongsheng Fu, Jian Wang, Weiguo Wang

**Affiliations:** 1 Clinical College of Chinese Medicine, Gansu University of Chinese Medicine, Lanzhou, China; 2 First Clinical Medical College, Shandong University of Traditional Chinese Medicine, Jinan, China; 3 College of Biology, Hunan University, Changsha, China; 4 Orthopedic Department, Affiliated Hospital of Shandong University of Traditional Chinese Medicine, Jinan, China

**Keywords:** anti-inflammation, anti-oxidative stress, bone metabolism, coumarins, musculoskeletal diseases, osteoarthritis, osteosarcoma, rheumatoid arthritis

## Abstract

Musculoskeletal diseases encompass a spectrum of pathologies such as osteoporosis, rheumatoid arthritis, osteoarthritis, and osteosarcoma, characterized by degenerative alterations and aberrant hyperplasia in bone mass, synovial tissue, and cartilage. Recently, botanical compounds have demonstrated promising potential in ameliorating skeletal and muscular aging. Coumarins, as naturally occurring phytochemicals, exhibit diverse pharmacological properties including anti-inflammatory, antiviral, antioxidant, antitumor, and immunomodulatory activities. This comprehensive review systematically elucidates how coumarins and their derivatives confer protection against Musculoskeletal Diseases primarily through modulation of inflammatory and oxidative mediators. These compounds target pivotal signaling pathways, such as NF-κB, MAPKs, Wnt/*β*-catenin, Sirt1, and PI3K/AKT, to enhance bone mineral density, mitigate cartilage degradation, suppress synovial pannus formation, and impede sarcoma cell migration. Despite their therapeutic promise, clinical translation of coumarins requires further investigation. Their dual capabilities in regulating bone metabolic homeostasis and immune responses, coupled with epigenetic interactions in musculoskeletal pathophysiology, provide valuable perspectives for retarding disease progression and advancing novel therapeutic interventions.

## 1 Introduction

Musculoskeletal diseases (MSDs) represent a class of systemic conditions that impair human morphology, skeletal movement, and muscular function, resulting from systemic degenerative processes and the progression of chronic inflammation (1). The musculoskeletal system comprises joints, tendons, ligaments, and synovium, among other components (2). Aging and traumatic injury are significant contributors to decreased bone mass, cartilage damage, degenerative changes in joint tissues, reduced muscle strength, and delayed fracture healing (3). Although often overlooked by patients due to their chronic nature—such as in osteoporosis (OP), rheumatoid arthritis (RA), osteoarthritis (OA), and osteosarcoma (OS)—these disorders progressively accumulate, leading to permanent functional impairment and significantly diminished quality of life (4). With the accelerating global aging population, the incidence of MSDs is rising, particularly among women aged 55–60, currently affecting approximately 1.7 billion people worldwide (5, 6). These conditions not only cause joint stiffness, swelling, and mobility limitations but also elevate the risk of other chronic diseases, including cardiovascular disorders, endocrine dysfunctions, and psychological burdens, making MSDs the third leading cause of non-communicable diseases (NCDs) globally (7). Beyond individual health impacts, MSDs exert substantial pressure on healthcare costs and social productivity (8). Conventional treatments for MSDs—such as exercise therapy, non-steroidal anti-inflammatory drugs (NSAIDs), hormone supplements, and laser therapy—have made incremental progress in pain relief, while surgical intervention remains necessary for severe cases (9). However, long-term use of these approaches carries risks of hepatorenal toxicity and gastrointestinal ulcers, and surgeries may lead to varying degrees of post-operative tissue damage (10). Therefore, the development of safer and more effective therapeutic strategies for MSDs warrants further exploration.

For millennia, natural herbs have served as an indispensable source of bioactive plant compounds and have played a significant role in the management of various degenerative diseases, including neurological, immune, and endocrine disorders (11, 12). Advances in scientific technology have further enabled the development of innovative herbal formulations and the extraction of high-value metabolites from these plants (13). A distinctive advantage of plant-derived metabolites—whether used as alternatives to synthetic drugs or as complementary therapies—lies in their favorable safety profile, characterized by lower toxicity and fewer side effects. They exert multifaceted functions, including anti-inflammatory, antioxidant, and anti-aging effects, as well as the regulation of metabolic homeostasis, thereby improving patient compliance and biocompatibility (14, 15). Coumarins, a class of secondary metabolites derived from the phenylpropanoid pathway, are found in over 30 plant families, such as Fabaceae, Asteraceae, Apiaceae, Rutaceae, Moraceae, and Lamiaceae (16). Coumarins exhibit a broad spectrum of pharmacological activities—such as anti-inflammatory, analgesic, antimicrobial, antioxidant, immunomodulatory, antitumor, antidiabetic, anticoagulant, and cardioprotective effects—and are widely distributed in polyphenol-rich plants including angelica (*Angelica archangelica*), soybeans, chamomile (*Matricaria chamomilla*), fennel (*Foeniculum vulgare*), and citrus fruits (Citrus spp.) (17–19). Owing to their extensive pharmacological potential, natural coumarins now demonstrate promising applications in drug discovery, healthcare, and wellness industries (20). Consequently, growing research attention is being directed toward exploring their medicinal value.

## 2 Methods

This systematic review was conducted in accordance with the Preferred Reporting Items for Systematic Reviews and Meta-Analyses (PRISMA) guideline and checklist (21).

### 2.1 Search strategy

Systematic searches were conducted in Web of Science, PubMed, and Embase databases for *in vivo* and *in vitro* studies published between January 1, 2000, and September 30, 2025, focusing on coumarins in the treatment of osteoporosis (OP), rheumatoid arthritis (RA), osteoarthritis (OA), and osteosarcoma (OS). The detailed search strategy is presented in Supplementary Table S1. A literature search was performed using a combination of keywords and Medical Subject Headings (MeSH) terms, connected by the Boolean operators “AND” or “OR.” The main search terms were: “linear furan coumarins OR angular furan coumarins OR linear pyranocoumarins OR angular pyranocoumarins OR coumarin OR osthenol OR fraxetin OR demethylsuberosin OR scopoletin OR esculetin OR umbelliferone OR Psoralen OR bergaptol OR Seselin OR xanthyletin” AND “osteoporosis OR bone loss OR decreased bone density OR osteoarthritis OR cartilage injury OR rheumatoid arthritis OR osteosarcoma.” All published articles were retrieved, and the titles and abstracts were screened.

### 2.2 Selection criteria

The studies included in this report were original or interventional, focusing on the effects of coumarins on musculoskeletal health, and reported outcomes related to OP, RA, OA, and OS. The PICO framework (Population, Intervention/Exposure, Comparison, Outcome) was used to screen relevant literature for this systematic review.

Inclusion criteria: studies investigating exposure to coumarins or coumarin-like compounds; studies involving musculoskeletal diseases including OP, RA, OA, and OS; studies with a controlled experimental design, i.e., including both control and experimental groups; experimental groups receiving different doses of coumarins or coumarin-like compounds, while control groups could receive blank control; comparisons involving *in vivo* or *in vitro* studies; original studies providing experimental data with clear experimental methods, appropriate interventions, defined comparators, and reliable outcomes. Detailed inclusion criteria are presented in Supplementary Table S2.

Exclusion criteria: studies not related to coumarin-like compounds; studies not involving musculoskeletal diseases (OP, RA, OA, and OS); non-original or non-interventional studies, such as review articles, meta-analyses, and case reports; studies involving combinations of coumarins with other compounds but not related to MSDs outcomes; studies written in languages other than English.

### 2.3 Data extraction

All records retrieved from the databases were imported into Zotero software as a library. Duplicate records were manually removed using the software tool and subsequently reconfirmed. Based on their titles and abstracts, two reviewers independently performed study eligibility screening and data extraction. Full-text review was then conducted, and quantitative data were organized into tables. Data were extracted from the full text of the included articles and entered into the corresponding columns. The information screened included compound source, exposure components, intervention model, route of administration, applied dose, therapeutic effects, biological targets, drug toxicity, and study limitations/future directions. Any discrepancies were resolved through discussion or by consultation with a third reviewer.

### 2.4 Risk of bias assessment

Two reviewers independently assessed study quality using a modified version of the OHAT Risk of Bias Tool for animal studies and the OHAT Risk of Bias Tool for *in vitro* studies (22–24). Any disagreements between the authors were resolved through discussion or by a third reviewer. Different domains of each included study were evaluated, with one question per domain. Full compliance with a domain and direct evidence of low risk of bias were recorded as “definitely low risk of bias (++).” Minor deviations or indirect evidence of low risk were marked as “probably low risk of bias (+).” Unclear information regarding the relevant risk of bias or indirect evidence of high risk was recorded as “probably high risk (−).” “Definitely high risk (−−)” was used for direct evidence of high-risk bias practices. The overall study assessment adopted a three-tier quality grading system (25, 26). Under this grading system, Tier I studies were considered high-quality articles, with >50% of criteria rated as definitely or probably low risk; Tier II studies were of moderate quality; and Tier III articles were of low quality, with >50% of criteria rated as definitely and/or probably high risk.

## 3 Results

### 3.1 Selection of studies

**Figure 1:**
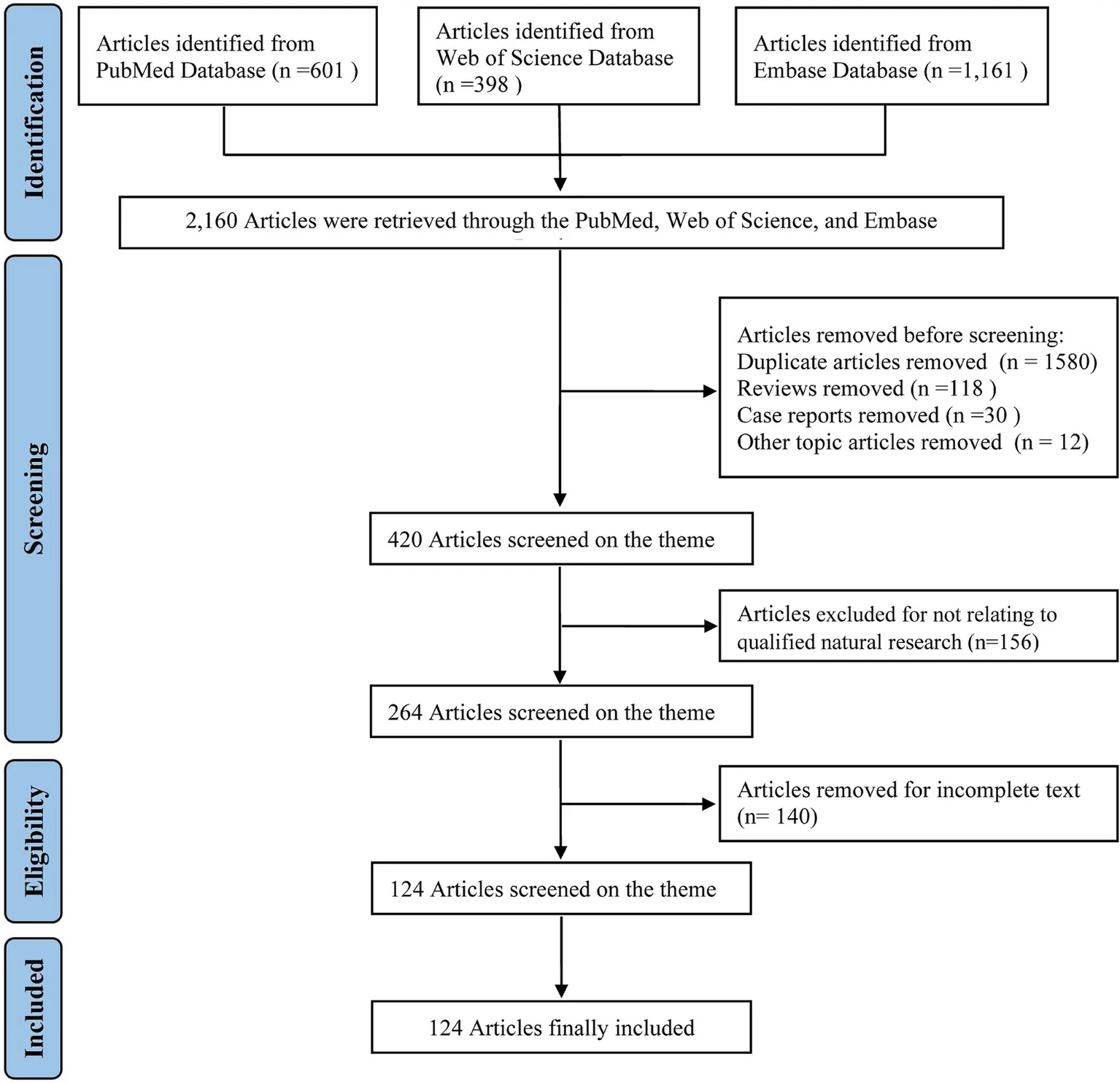
Flow diagram illustrating inclusion and exclusion criteria for literature on coumarin treatment for skeletal muscle disorders. 124 articles ultimately met the analysis criteria.

2,160 articles were identified from three databases (Web of Science, PubMed, and Embase). A total of 1,740 articles were removed due to duplication, being reviews, case reports, or focusing on other research topics. The remaining 420 articles were screened by title and for eligibility, and 156 articles were excluded. The full-text manuscripts of the remaining 264 articles were obtained for assessment. Of these, 140 full-text manuscripts were excluded due to incomplete text content. Finally, 124 articles were deemed eligible and included in the final analysis (Figure 1).

### 3.2 Risk of bias assessment

The included studies were assessed using the OHAT Risk of Bias Tool. Most *in vitro* studies exhibited a low risk of bias in terms of selection bias, performance bias, attrition/exclusion bias, detection bias, and reporting bias. None of the included studies clearly described blinding or allocation concealment. In most studies, the purity of coumarins was not disclosed in the assessment of exposure characteristics; information on coumarin treatment duration and tested concentrations or treatment concentrations for cell models was also lacking. More than half of the included studies showed inconsistencies in treatment conditions or failed to disclose some or all parameter information (Supplementary Tables S3–S6). All included studies were rated as Tier I [except for the study by Takashima et al. (27)], based on more than 50% of the domains being classified as definitely and/or probably low risk of bias. The study by Takashima et al. (27) had unclear experimental conditions and study outcomes.

In animal studies, most studies exhibited a low risk of bias in terms of performance bias, attrition/exclusion bias, detection bias, and reporting bias. No studies reported allocation concealment or blinding assessment. Incomplete reporting was observed regarding randomization, coumarin purity in exposure characteristics, and information on controlled diet/dietary coumarin intake (Supplementary Tables S7–S10). No studies were rated as Tier III (low-quality articles with >50% of criteria rated as definitely and/or probably high risk).

### 3.3 Clinical application of coumarins in the musculoskeletal system

**Figure 2:**
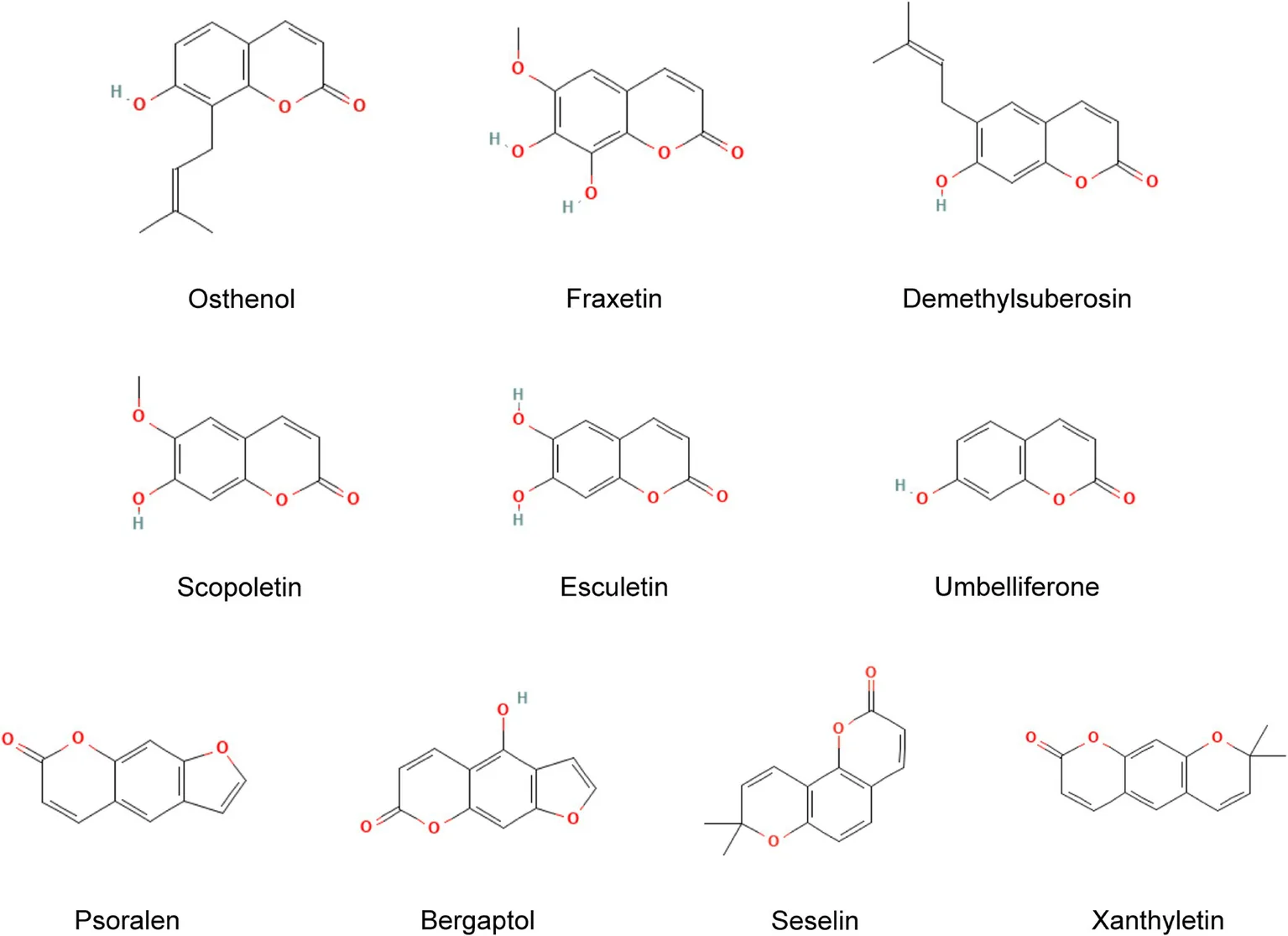
Chemical structures of 10 major coumarins. Based on pyrone ring structures, the top six structures are simple coumarins, and the latter four are complex coumarins.

Natural coumarin was first isolated in 1820 from the leguminous plant (*Dipteryx odorata* Wild) and is found in a wide variety of natural species, possessing a characteristic aromatic odor (28). To date, over 1,300 coumarin species have been discovered. In addition to being found in the roots, leaves, fruits, and flowers of plants, coumarins have also been identified in bacteria, fungi, and marine products (29). Based on their structural characteristics, coumarins are mainly classified into simple coumarins and complex coumarins. Complex coumarins are primarily divided into furanocoumarins (linear and angular), pyranocoumarins (linear, angular, and condensed types), substituted coumarins, and other types of coumarins (such as 4-phenylcoumarins and isocoumarins) (30–33). Under glycosylation, coumarins exert potent antioxidant capacity by scavenging oxygen free radicals (34). The chemical structures of ten major coumarins are shown in Figure 2.

Given their anti-inflammatory and anti-aging pharmacological effects, coumarins may represent promising agents against age-related musculoskeletal decline. Various coumarin compounds have been employed to advance research on muscle–bone–immune interactions, such as Urolithin A. Urolithin A (UA), also known as phenylcoumarin or dibenzopyrone, ameliorates age-related mitochondrial dysfunction accumulation and muscle function decline (35). Whitfield et al. demonstrated that daily administration of 1,000 mg of Urolithin A improved mitochondrial function in the skeletal muscle of clinical subjects, downregulated CRP inflammatory responses, alleviated muscle damage, and enhanced aerobic exercise capacity (36). Zhao et al. found that after an 8-week observation period, all participants receiving 1 g/day of UA exhibited good safety and tolerability within one month following the intervention and after the completion of the study, along with reduced levels of 3-MH, a marker of skeletal muscle protein breakdown (37). Singh et al. also found that the daily 500 mg UA group significantly downregulated plasma levels of acylcarnitines in participants, improved fatty acid oxidation, and promoted the levels of the glycogen debranching enzyme AGL and myophosphorylase PYGM, thereby influencing muscle aging and metabolism (38). Liu et al. reported that although participants taking UA showed no significant improvements in the 6-min walk distance or maximal ATP production in hand muscles, long-term daily administration of 1,000 mg UA significantly enhanced skeletal muscle-specific endurance in older adults and improved metabolic markers of mitochondrial function (39). Coumarins not only focus on degeneration and tissue repair in muscle/tendon–aerobic metabolism but also regulate osteoclast differentiation and skeletal degeneration induced by immune system cell lineages (monocytes/macrophages) (40). One study found that coumarins alleviated impaired bone turnover and remodeling in diabetic osteoblasts and osteoclasts by disrupting hyperglycemia-mediated AGE-RAGE interactions (41). These findings progressively reveal the future prospects of coumarin research and application in musculoskeletal degeneration.

Although coumarins have been demonstrated to possess various nutritional and pharmacological properties, the demand for their synthetic organic chemical extraction and derivatives continues to increase (42). Most commercial applications of coumarins are achieved through synthesis, primarily in food processing, fragrance production, and the rubber industry (43). In the medical field, coumarins and their derivatives have been used in *in vitro* experiments, animal models, and human studies to realize their pharmacophysiological potential in treating various diseases, such as targeted kinase intervention in degenerative diseases, screening of coumarin nuclear substituents for anticancer significance, and regulation of Th17/Treg immune cell activity (44–46). In 1954, the United States Food and Drug Administration (FDA) banned the use of coumarin in food due to the hepatotoxicity of coumarin extracted from cinnamon (47, 48). Owing to the multiple metabolic benefits of cinnamon coumarin, Europe adjusted the tolerable daily intake (TDI) of coumarin to 0.1 mg/kg/day to prevent hepatotoxicity and ensure clinical safety (49). However, fundamental studies on acute oral toxicity have found that esculetin and the citrus-derived auraptene did not cause death or clinical toxicity at LD50 doses of 125–2000 mg/kg/day, even when validated in animals (50). In clinical studies, alternative coumarins may mitigate their toxic effects and, in some cases, may have positive effects in humans (51). Due to differences in species origin, synthetic coumarins or those used in combination with other drugs are safe and well-tolerated in most patients, with only a small proportion showing signs of mild liver injury (52, 53). Another existing assessment of hepatotoxicity risk found that even when cinnamon bark coumarin intake exceeded the TDI, the incidence of coumarin-induced abnormal liver function changes was low, allowing for safe use in clinical practice (54). Therefore, as a dietary nutritional supplement, coumarin remains an important candidate for promoting health benefits within a reasonable applied dose.

### 3.4 Effects of coumarin in musculoskeletal diseases

**Figure 3:**
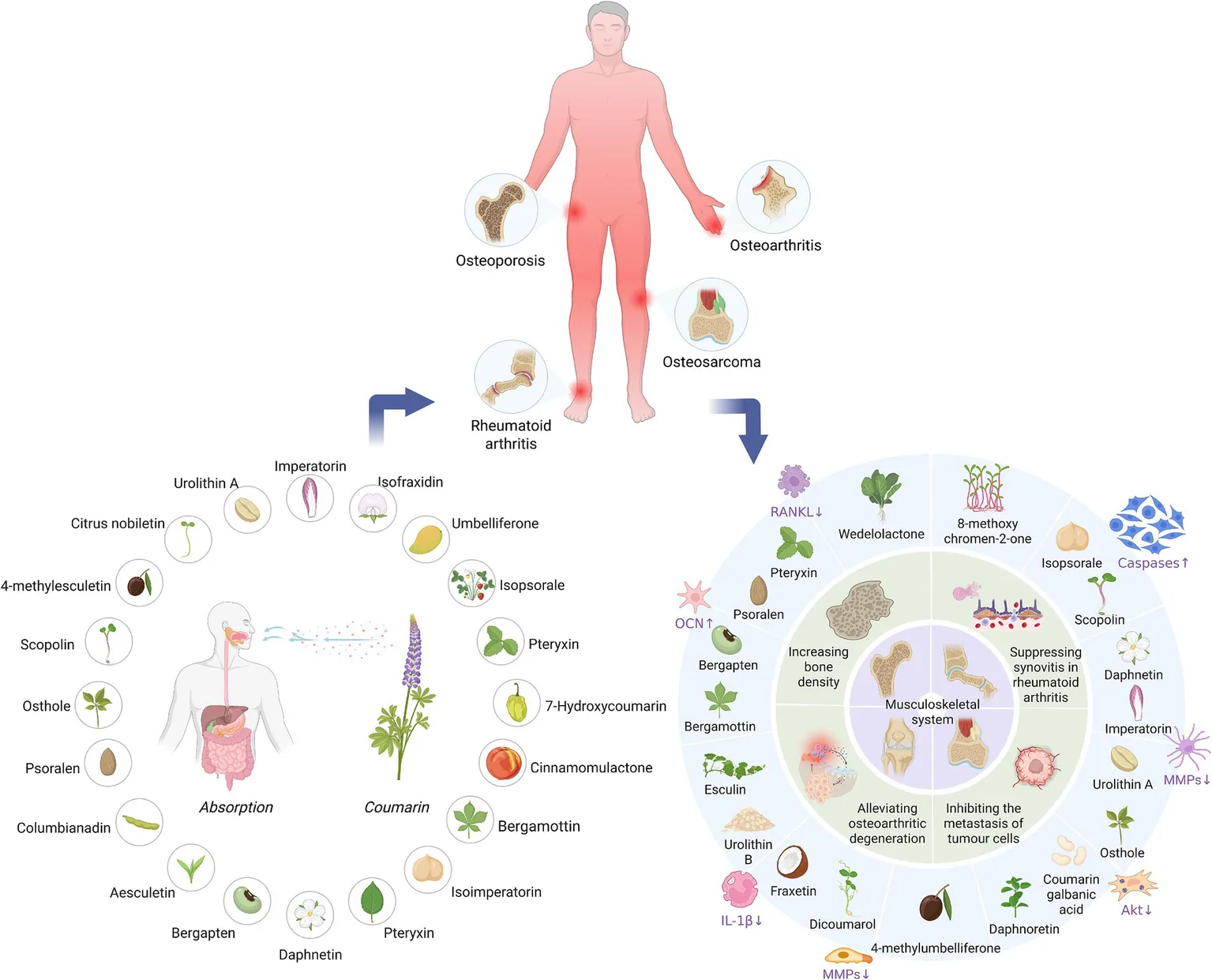
Mechanisms of coumarin in alleviating musculoskeletal diseases. Coumarin, extracted from various natural plants, targets cytokines and signaling pathways involved in osteoporosis, rheumatoid arthritis, osteoarthritis, and osteosarcoma. ↑, promotion; ↓, inhibition.

Musculoskeletal diseases, such as osteoporosis (OP), rheumatoid arthritis (RA), osteoarthritis (OA), and osteosarcoma (OS), pose major challenges to global healthcare systems. As research into the mechanisms of coumarin in managing these diseases continues to expand, it holds promise for bridging the gap between emerging fields and established therapeutic strategies (Figure 3). This review Systematically summarized current studies and mechanisms of coumarin across various musculoskeletal diseases, highlighting significant findings to support further pharmacological investigation and clinical exploration.

#### 3.4.1 Mechanisms of coumarin in osteoporosis

##### 3.4.1.1 Overview of coumarin

Osteoporosis (OP) is a progressively degenerative skeletal metabolic disorder characterized by reduced bone strength, decreased bone mineral density, and increased bone fragility, with advanced stages frequently leading to fractures (55). Major risk factors include declining estrogen levels, genetic predisposition, inadequate intake of nutrients such as calcium, and long-term medication-induced bone loss (56). The core pathological mechanism involves an imbalance between osteoclast-mediated bone resorption and osteoblast-driven bone formation (57). Disruption of bone metabolic homeostasis activates the OPG/RANKL/RANK signaling cascade, promoting osteoclast activity while impairing osteoblast-mediated bone regeneration, ultimately leading to bone loss and aberrant remodeling (58). The condition is broadly categorized into primary and secondary OP. Primary OP is typically age-related, whereas secondary OP often results from factors such as excessive alcohol consumption or glucocorticoid use (59). To counteract bone loss, various *in vivo* and *in vitro* OP models, including ovariectomy, aging, and steroid-induced systems, have been established to facilitate the development of safer and more effective targeted therapies for OP (60).

##### 3.4.1.2 Effects of coumarins on bone quality in animal models

The destruction of bone microstructure is a prominent characteristic of OP, with its restoration serving as a key criterion for evaluating drug efficacy (61). Primary osteoporosis constitutes the major component of OP, with the majority of postmenopausal women presenting with primary OP, a condition closely associated with estrogen deficiency. In animal models of primary OP established using OVX, coumarins have been shown to increase bone density and trabecular bone mass while reducing bone loss within the effective dose range. At the microstructural level, OP may be reflected by significant changes in bone mass and trabecular microstructure in the femur (62). Urolithin A (UA), a coumarin-derived compound produced from nuts, has been found to have beneficial effects on aging and age-related diseases (63). An OP mouse model was established via OVX and orally treated with different doses of UA (once daily, 10–20 mg, for 8 weeks). After treatment, mice showed increased trabecular thickness (Tb. Th) and number (Tb. N), bone volume fraction (BV/TV), and bone mineral density (BMD), as well as decreased trabecular separation (Tb. Sp). Three-dimensional (3D) image reconstruction demonstrated that UA significantly reduced trabecular bone loss in the distal femur, and no obvious side effects or damage to metabolic organs (liver and kidney) were observed (64). Pteryxin (PTX) has the potential to inhibit osteoclast activity and differentiation in OP. In one study, PTX was administered via intraperitoneal injection (once every two days, 5–10 mg/kg, for 6 weeks), which increased BV/TV and Tb. N and significantly decreased Tb. Sp in mouse trabecular bone (65). Under Micro-CT observation, the novel compound BX isolated from Psoralen (Pso) significantly increased Tb. N in OVX mice. Oral administration (once daily, 15–135 mg/kg, for 12 weeks) promoted trabecular bone growth and density, significantly increasing BMD, BV/TV, Tb. Th, and Tb. N, while inhibiting Tb. Sp levels (66). Another study demonstrated that Psoralen (Pso) administered orally (once daily, 10–40 mg, for 4 weeks) targeted the inhibition of HSD17B2 in the liver of OVX mice to enhance estrogen levels, thereby increasing trabecular bone and bone marrow cavity filling (67). Isoimperatorin (ISO), a furanocoumarin, promoted trabecular bone formation in OVX mice via oral administration (once daily, 5–10 mg/kg, for 8 weeks), as evidenced by increased BMD, BV/TV, Tb. N, and ALP levels, and decreased Tb. Sp (68). However, ISO did not appear to significantly affect Oc. S/BS in mice, indicating that ISO does not interfere with osteoclastogenesis in OVX mice. Nodakenin administration (once daily, 20 mg/kg, for 6 weeks) improved BMD and bone microstructure parameters in OVX mice, increasing BMD, Tb. N, BV/TV, and SMI levels (69). Yamamoto and Takashima found that novel 4,6-substituted coumarin derivatives play an important role in promoting cortical bone osteogenesis in OVX rats. KY-054, a novel 4,6-substituted coumarin, administered orally (once daily, 10 mg/kg, for 8 weeks), increased metaphyseal and diaphyseal cortical bone volume (Ct. BV) and cortical bone mineral content (Ct. BMC) without affecting medullary volume (Med. V) (70). Takashima et al. (27) found that the novel KY-054 (once daily, 10 mg/kg, for 8 weeks) inhibited cyclin-dependent kinase 8 (CDK8) activity, thereby promoting cortical Ct. BV and Ct. BMC levels in rats. Among the derivatives, 2-oxo-4-[4-(tetrahydro-2H-pyran-4-yloxymethyl)phenyl]-2H-chromene-6-carboxamide (11 m) and 2-oxo-4-[4-(tetrahydro-2H-pyran-4-ylmethoxy)phenyl]-2H-chromen-6-carboxamide (29v) exhibited the strongest osteogenic activity.

Dharmendra et al. (71) demonstrated that Dalbergin (DBN) administered to OVX mice (once daily, 1–10 mg/kg for 8 weeks) significantly increased BV/TV and Tb. N in trabecular bone of the tibia and lumbar vertebrae, and promoted BMP2 expression to counteract bone loss. Ham et al. (72) found that supplementation of the diet with 0.02% Methoxsalen for 8 weeks upregulated the expression of Runx-2, OSX, OCN, and ALP in mouse osteoblasts, alleviating OVX-induced bone loss and bone microstructural deterioration. Bergapten (BP) administered orally (once daily, 20 mg/kg for 12 weeks) improved the trabecular bone microstructure of the femur and increased the expression levels of RUNX2 and OCN in mice (73). These findings suggest that the improvement of trabecular bone microarchitecture by coumarins in animal studies of OP may be associated with the regulation of BMP2 and RUNX2 expression in osteoblasts. Wedelolactone was administered intraperitoneally to mice (once every two days, 10 mg/kg for 4 weeks), increasing BV/TV and Tb. N and promoting new bone formation (74). Bergamottin (BM) (once daily, 15–30 mg/kg for 8 weeks) increased BV/TV, Tb. N, Tb. Th, and BMD levels in OVX mice, and inhibited trabecular separation to alleviate bone loss (75). Sashidhara et al. found that oral administration of coumarin-dihydropyridine hybrids (once daily, 1–10 mg/kg for 8 weeks) increased BV/TV, Tb. N, SMI levels, and the OPG/RANKL ratio, and inhibited osteoclast activity to promote trabecular bone microstructural recovery in OVX mice (76). These studies indicate that coumarins may have the potential to inhibit osteoclast differentiation in primary OP animal models by regulating the OPG/RANKL molecular mechanism, thereby alleviating bone resorption.

Secondary osteoporosis is triggered by diseases, medications, or nutritional deficiencies when bone becomes fragile, including factors such as glucocorticoids, chronic kidney disease, diabetes mellitus, and malnutrition (77). One animal study on secondary OP found that oral administration of Isopsoralen (once daily, 5–20 mg/kg for 4 weeks) reduced cGMP levels in bone tissue of glucocorticoid-induced OP mice, and increased PKG and RUNX2 expression to promote osteoblast differentiation *in vivo* (78). Li et al. (79) found that intraperitoneal injection of Osthole (once daily, 5 mg/kg for 8 weeks) increased BMD, BV/TV, and Tb. Th in the vertebrae of nephrectomized mice, and inhibited TRAP and MMP-9 expression on the trabecular bone surface, thereby ameliorating bone loss. Ham et al. (80) found that BP and MTS were added to a high-fat diet in mice at a concentration of 0.02%, which increased BV/TV, Tb. N, and Tb. Th in the femur of diabetic mice, promoted the gene expression of RUNX2, OPG, OCN, and TRAP, reduced the gene expression of NFATc1 and OSX, and improved the skeletal microstructure of diabetic mice. Li et al. (81) found that oral administration of Bergapten (once daily, 10–20 mg/kg for 20 weeks) inhibited the JNK/MAPK, PI3K/AKT/mTOR, and NF-κB inflammatory signaling pathways, reduced RANKL-RANK activity in mice, and decreased osteoclast differentiation and maturation in diabetes-related OP. These studies further support the potential of coumarins to modulate secondary OP. Therefore, coumarin-related compounds are potential dietary supplements for enhancing bone quality, and their mechanism of action may involve enhancing trabecular bone microstructure *in vivo*, reducing bone marrow cavity cavitation, improving systemic metabolism and bone mineral density, and alleviating inflammation to prevent bone loss in animal models of OP.

##### 3.4.1.3 Cellular and molecular mechanisms of coumarins in the prevention of osteoporosis

###### 3.4.1.3.1 Regulation of BMP-2 and Runx2 to promote osteogenesis

Osteoblasts play a crucial role in generating organic bone matrix and enhancing bone matrix mineralization, thereby maintaining bone microstructure and homeostasis (82). The Runx family of transcription factors regulates gene expression involved in skeletal development, including bone cell proliferation, differentiation, and mineralization (83). Among them, Runx2 activity and transcription significantly influence osteoblast formation, with dynamic changes in RUNX2-DNA binding sites affecting stage-specific osteogenic differentiation (84). Runx2 activates osteoblast-specific genes such as osteopontin (OPN), alkaline phosphatase (ALP), and osteocalcin (OCN), promoting mesenchymal stem cell (MSC) differentiation and skeletal growth (85). Xue et al. demonstrated that Wedelolactone (1 μg/mL, 72–108 h) increased the expression of ALP, RUNX2, and OPN in bone marrow mesenchymal stem cells (BMSCs) in a concentration-dependent manner, promoting osteoblastogenesis and bone mineralization (74). Sashidhara et al. found that coumarin-dihydropyridine hybrids (1–100 pM, 48 h) enhanced the expression of osteogenic genes, including RUNX2 and Col-1, in mice, promoted bone formation rate (BFR) and mineral apposition rate (MAR), and ameliorated bone resorption (76). Both Psoralen (20 μg/mL, 24–72 h) and Isopsoralen (2–50 μM, 24–72 h), derived from *Psoralea corylifolia*, were shown to promote osteoblast differentiation and maturation by inhibiting miR-488 expression and enhancing estrogen receptor *α* (ERα) to target RUNX2 expression, respectively (86, 87). Aesculetin (1–10 μM, 36–60 h) promoted the activation of OPN, ALP, and RUNX2 in MC3T3-E1 cells, with the strongest activity and mineralization observed in differentiating osteoblast matrix vesicles when treated with ≥5 μM Aesculetin (88).

Runx2 regulates osteoblast progenitor differentiation and proliferation by inducing Wnt signaling pathway genes, with the Wnt/*β*-catenin pathway playing a key role in osteoblastogenesis and ossification. One study found that Wedelolactone (6 μM, 9 days) induced phosphorylation of GSK-3*β*, which elevated the expression of casein kinase 2α (CK2α), promoted the nuclear transcription of Wnt5a, β-catenin, and Runx2, and enhanced osteoblast differentiation and bone mineralization at a concentration of 30 μM (89). Although GSK3β traditionally serves as a negative regulator of glycogen synthesis, its phosphorylation-mediated regulation in osteogenic control increases *β*-catenin nuclear translocation to promote osteoblast differentiation. Isoimperatorin (5 μM, 14 days) significantly increased the mRNA expression of RUNX2, OPN, and BGLAP in MC3T3 cells, promoted ALP activity, and enhanced osteogenic differentiation (68). Xiao et al. demonstrated that Bergapten (0.1–10 μM, 14 days) upregulated the Wnt/*β*-catenin pathway, increased RUNX2 and ALP expression, and promoted the differentiation of BMSCs into osteoblasts (73). Bergamottin (BM) (5–20 μM, 24 h) was shown to enhance the expression of T-cell factor 7 (TCF7) and activate Runx2 activity in osteoblasts by activating the Wnt/*β*-catenin signaling pathway (75). Yan et al. found that Imperatorin (25–75 μM, 24 h) increased the levels of RUNX2, COL1A1, and OC in bone marrow osteoblasts by promoting GSK3β Ser9 phosphorylation and β-catenin transcription (90). Therefore, the stimulatory effect of Runx2 on osteoblasts may depend on GSK3β, which plays an important role in coumarin-regulated Wnt/β-catenin signaling pathways.

Deficiency or dysfunction of BMP2 protein can impair skeletal growth and maturation, and RUNX2 differentiation is closely linked to BMP2 activity (91). Kumar et al. (92) found that Dalbergin (DBN) (1 pM–100 μM, 48 h) promoted the expression of osteoblast genes Runx2, ALP, OCN, and COL1 by 4- to 5-fold compared with the control group. Isocoumarin A (2.5–10 μM, 24–72 h) increased the protein levels of BMP-2, RUNX2, and ALP in MC3T3-E1 cells in a dose-dependent manner, activated the Akt/Erk1/2 signaling pathway, and promoted the mineralization of the osteoblast extracellular matrix (93). BMP2 binding to membrane receptors activates multiple signaling pathways, including Wnt and MAPK, and facilitates Smad1/5/8-mediated Smad4 translocation to downstream targets, enhancing the differentiation and maturation of ALP- and RUNX2-related osteogenic factors (94). Kuo et al. found that Osthole (5–20 μM, 48–72 h) was shown to mediate multiple signaling pathways including BMP-2, Erk1/2, and p38 MAPK to alleviate OP progression (95). Osthole (10^−4^ mol/L, 6–12 days) promoted osteoblast differentiation by significantly increasing *β*-catenin-BMP signaling (96). Furthermore, BMP2 also increased osteoblast differentiation in Osthole-treated rat osteoblasts through the downstream p38 MAPK/Runx-2 pathway (97). Basem M. et al. (98) found that 5’-Hydroxy Auraptene (5’-HA) (10–50 μM, 5–12 days) significantly induced osteoblast proliferation by activating Smad1/4 phosphorylation and increasing BMP2 expression in BMSCs, while the BMP inhibitor LDN-193189 significantly suppressed 5’-HA-induced osteoblast stimulation and differentiation. Psoralen (10–100 μM, 48 h) increased the mRNA expression of BMP2 and BMP4, particularly the 12xSBE-OC-Luc gene, and increased the phosphorylated protein levels of Smad1/5/8, thereby promoting the expression of the target gene Osx to enhance osteoblast differentiation (99). Imperatorin and Bergapten (0.3–10 μM, 48 h) increased BMP-2 gene levels in osteoblasts through the Smad1/5/8 and p38 MAPK/Erk signaling pathways, thereby inducing ALP activity and promoting osteoblast differentiation and maturation (100). In summary, multiple coumarins such as Isocoumarin A, Osthole, and Psoralen may regulate BMP2 through various signaling pathways (e.g., Erk1/2), ultimately targeting the expression and transcription of the downstream osteogenic factor Runx2.

###### 3.4.1.3.2 Regulation of OPG/RANKL signaling to suppress osteoclastogenesis

Receptor activator of nuclear factor kappa-B ligand (RANKL), a member of the tumor necrosis factor (TNF) ligand family, binds to the RANK receptor on osteoclast surfaces to drive osteoclastogenesis, maturation, and differentiation (101). Osteoprotegerin (OPG), produced by osteoblasts and osteocytes, acts as a decoy receptor that competitively binds RANKL, thereby inhibiting osteoclast maturation, differentiation, and bone resorption (102). The OPG/RANKL ratio serves as a key indicator of bone formation, resorption, and skeletal integrity (103). Dharmendra et al. (71) found that DBN (1 pM–100 μM, 24–48 h) reduced the expression of the osteoclast markers TRAP and RANK, increased the OPG/RANKL ratio in osteoblasts, and improved bone microstructure. Osthole (0.1–10 μM, 7 days) upregulated the OPG/RANKL ratio, inhibited osteoclast formation, and suppressed NFATc1, c-Fos, and MMP-9 expression to ameliorate bone loss (79). Psoralidin (10^−6^ mol/L, 9–12 days) promoted the proliferation and differentiation of ROB cells, significantly suppressed RANKL expression, and increased the mRNA expression of IGF-1, *β*-catenin, Runx-2, and the OPG/RANKL ratio, thereby promoting osteoblast formation and inhibiting osteoclast activity (104). These studies suggest that coumarins may influence osteoclast activity by upregulating OPG/RANKL expression, further elucidating the important role of OPG in bone formation.

Nuclear factor of activated T cells 1 (NFATc1), activated by RANKL, plays a central role in regulating the formation and differentiation of osteoclast precursors and serves as a master transcription factor for osteoclast fusion and bone resorption (105). NFAT promotes NFATc1 expression feedback and nuclear translocation (106). Activated NFATc1 drives the expression of key osteoclast genes, ncluding Dcstamp, Atp6v0d2, Oscar, Ocstamp, Ctsk, and Calcr, promoting osteoclast precursor maturation and functional protein expression (107). Multiple coumarin compounds, such as Isoimperatorin (ISO) (4–8 μM, 1–7 days), Osthole (5 μM, 2–4 days), and Psoralidin (1–30 μM, 48 h), inhibited the binding of RANK to RANKL, reduce the expression of downstream NFATc1 and c-Fos, thereby decreasing osteoclast formation and alleviating bone resorption (108–110). In addition, the calcium (Ca^2+^) signaling pathway is crucial for enhancing NFATc1 activity, and intracellular Ca^2+^ flux is involved in the auto-amplification of NFATc1 and the expression of downstream osteoclast-related factors. Pteryxin (PTX) (10–20 μM, 48–96 h) blocked the MAPK and Ca^2+^/calcineurin-NFATc1 signaling pathways, reduced the activity of NFATc1 and osteoclast-specific genes, and inhibited osteoclast formation and bone loss (65). Jeong et al. (111) found that praeruptorin A (10 μM, 72 h) reduced RANKL-induced Ca^2+^ activity, suppressed the expression levels of downstream NFATc1-related molecules, and prevented osteoclast fusion. Xanthotoxin (XAT) (0.1–1 μM, 72 h) downregulated Ca^2+^ oscillation expression in bone marrow macrophages (BMMs), inhibited the downstream calcium-CaMKK/PYK2 pathway and NFATc1 activity, and restored bone strength (112). These findings suggest that coumarins may regulate the Ca^2+^ pathway to suppress the transcription and expression of NFATc1, and multiple signaling pathways are also involved in the RANKL/Ca^2+^/NFATc1 cascade during osteoclast formation.

RANKL binding to RANK on osteoclast precursors activates the NF-κB signaling pathway, which is critical for osteoclast differentiation and formation (113). Activation of IκBα in the NF-κB pathway promotes the transcription of subunits NF-κB p50 and NF-κB p65, enabling their binding to downstream osteoclast target genes (114). Multiple pharmacological studies of coumarin compounds, including Urolithin A (5–10 μM, 48–96 h), Aesculin (5–20 μM, 3–5 days), Bergapten (1.2–5 μmol/L, 5–7 days), Osthole (10^−6^ mol/L to 10^−5^ mol/L, 1–7 days), Osthole-loaded N-octyl-O-sulfonyl chitosan micelles (NSC-OST) (10^−5^ mol/L, 48 h), Citrus nobiletin (4–50 μM, 72 h), and Isofraxidin (6.25–25 μM, 24 h), have demonstrated that they inhibit RANKL-induced IκB phosphorylation and p65 ubiquitination levels in the NF-κB pathway, thereby suppressing osteoclast gene expression and bone resorption (64, 115–120). Furthermore, other studies have found that Praeruptorin B (5–10 μM, 1–6 days) and Urolithin B (1–25 μM, 12–72 h) downregulated ERK phosphorylation levels and enhanced the expression of glutathione S-transferase Pi 1 (GSTP1), respectively, to reduce NF-κB p65 expression and inhibit osteoclastogenesis and activity (121, 122). Therefore, coumarin phytochemicals may have the potential to block osteoclastogenesis and bone destruction by inhibiting the RANKL-induced NF-κB pathway. Moreover, the NF-κB pathway is regulated by multiple upstream signaling pathways, including GSTP1 and MAPK isoforms, to alleviate the progression of OP.

**Figure 4:**
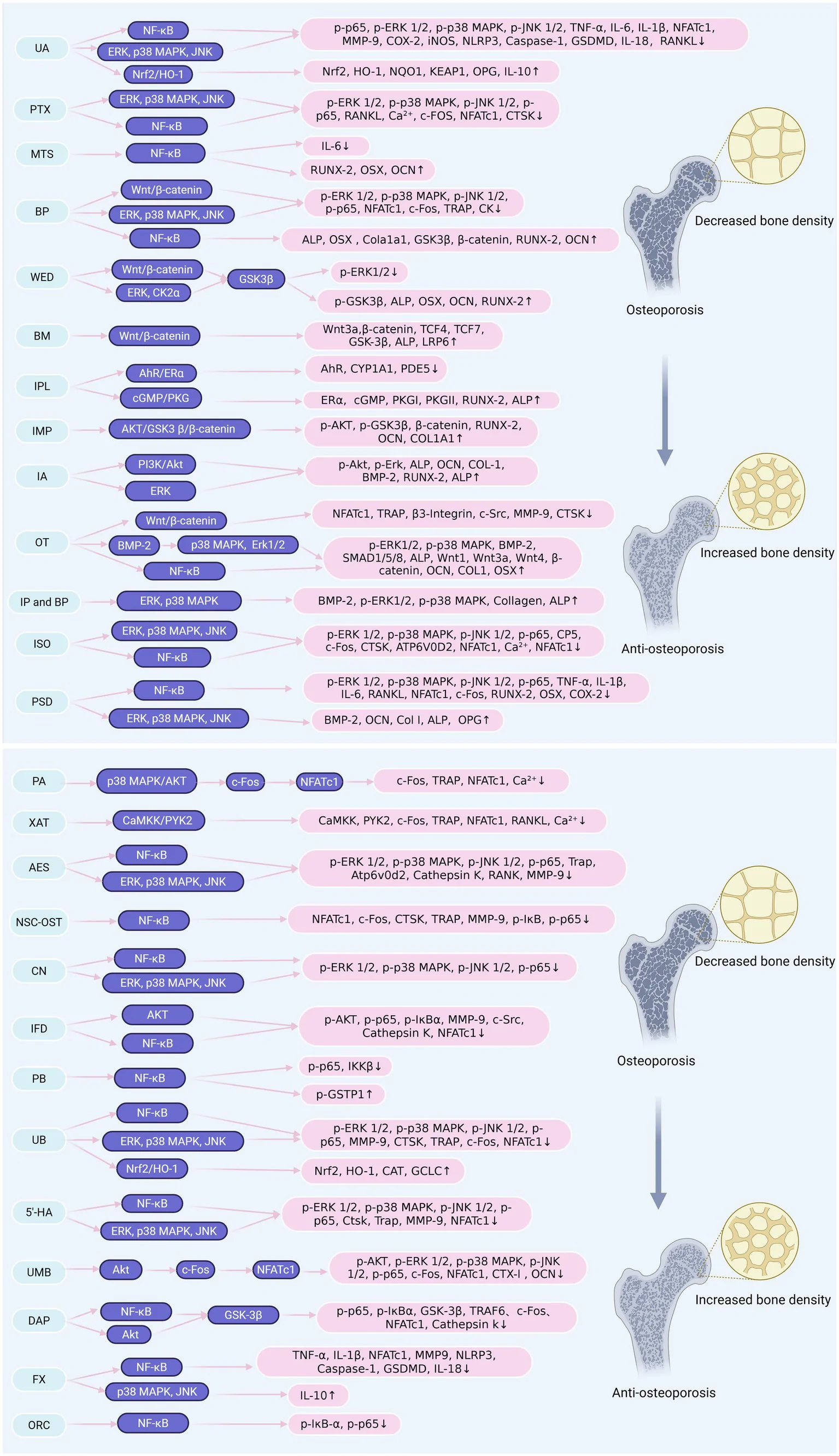
Effects of coumarins on osteoporosis and mechanisms for improving bone mineral density via multiple signaling pathways. UA, urolithin A; PTX, pteryxin; MTS, methoxsalen; BP, bergapten; WED, wedelolactone; BM, bergamottin; IPL, isopsoralen; IMP, imperatorin; IA, isocoumarin A; OT, osthole; ISO, isoimperatorin; PSD, psoralidin; PA, praeruptorin A; XAT, xanthotoxin; AES, aesculin; NSC-OST, osthole-loaded N-octyl-O-sulfonyl chitosan micelles; CN, citrus nobiletin; IFD, isofraxidin; PB, praeruptorin B; UB, urolithin B; 5’-HA, 5′-hydroxy auraptene; UMB, umbelliferone; DAP, daphnetin; FX, fraxin; ORC, orcinol. ↑, promotion; ↓, inhibition.

RANKL-RANK complex formation recruits TRAF6 and activates downstream MAPK pathways—including ERK, JNK, and p38 MAPK—which promote early osteoclast survival and differentiation while influencing osteoblast proliferation (123). Praeruptorin A (10 μM, 72 h) inhibited RANKL-induced p38 and Akt signaling, reduced osteoclast differentiation stimuli, and downregulated c-Fos and NFATc expression levels (111). Urolithin A (5–20 μM) inhibited RANKL-induced osteoclastogenesis by reducing the phosphorylation levels of JNK, Erk1/2, and p38 MAPK proteins (124). Basem M. et al. (125) found that 5′-hydroxy auraptene (1–50 μM) reduced RANKL-induced phosphorylation levels of ERK1/2, p38 MAPK, and JNK, and inhibited osteoclast differentiation in mouse bone marrow (BM) cells. TRAF6 is a key regulator of RANKL/RANK-triggered signaling cascades, and activation of the downstream PI3K/AKT cascade leads to impaired osteoblast differentiation and an osteoclastic phenotype (126). Umbelliferone (5–50 μM, 48–96 h) inhibited RANKL-induced osteoclast formation by suppressing the Akt/c-Fos/NFATc1 signaling pathway, and ameliorated osteoclast-specific gene expression in BMMs, including TRAP, OSCAR, and CtsK-mediated bone loss (127). Furthermore, both Bergapten (1.2–5 μmol/L, 5–7 days) and Daphnetin (20–40 μM, 96 h) have been demonstrated not only to inhibit osteoclast differentiation and maturation through the Akt/NFATc1 signaling pathway but also to intervene in the MAPK pathway to alleviate bone loss (116, 128). In summary, coumarin compounds may regulate osteocyte differentiation and bone resorption by modulating the activity of multiple pathways, including MAPK, NF-κB, and PI3K/AKT, in both osteoblasts and osteoclasts, and may positively regulate bone metabolic homeostasis by interfering with various cross-signaling pathways (Figure 4).

###### 3.4.1.3.3 Regulation of oxidative factors to ameliorate bone metabolism

Reactive oxygen species (ROS) play a significant role in bone metabolism signaling. Osteoclast precursors can generate substantial ROS under RANKL stimulation, and excessive ROS levels promote osteoclast formation and activity (129). TRAF6 and Nox1 act as endogenous activators of ROS production induced by RANKL, leading to antioxidant enzyme deficiency, accelerated osteoclast precursor formation, trabecular bone loss, and increased bone resorption (130). Multiple studies have demonstrated that Psoralidin (1–30 μM, 48 h), Osthole (16 μM, 48 h), and Notopterol (8 μM, 48 h) significantly inhibit the activity of nitric oxide (NO) and reactive oxygen species (ROS), and ameliorate bone resorption and bone loss in zebrafish OP models (108, 131). Excessive ROS production enhances the cascade reaction of NFATc1 and upstream Ca^2+^ signaling pathways, thereby regulating osteoclast activity and formation (132). Xanthotoxin (0.1–1 μM, 72 h) inhibited RANKL-induced Ca^2+^ oscillations and ROS activity in bone marrow macrophages, downregulated activated NFATc1 and c-FOS levels, and suppressed osteoclast maturation and bone loss in mice (112). Excessive ROS generation not only affects osteoblast differentiation and maturation but also stimulates the MAPK pathway to increase RANKL-stimulated osteoclast differentiation and maturation (133). Pteryxin (PTX) (10–20 μM, 48–96 h) and Isoimperatorin (4–8 μM, 1–7 days) reduced the activity of the MAPK/Ca^2+^ signaling pathway to inhibit ROS expression, downregulated NFATc1 gene expression in osteoclasts, and suppressed osteoclast maturation and differentiation (65, 108). Urolithin B (10–100 μM, 2–5 days) and Fraxin (5–10 μM, 1–5 days) increased the levels of ROS-scavenging enzymes in OVX rats to inhibit ROS accumulation, suppressed the MAPK and NF-κB pathways to reduce NFATc1 expression and activity, and hindered osteoclast differentiation and maturation (134, 135). Therefore, coumarin compounds may protect bone health by modulating signaling pathways such as MAPK and Ca^2+^, reducing ROS generation to enhance antioxidant stress levels, and alleviating NFATc1-induced dysfunction in bone metabolism.

###### 3.4.1.3.4 Regulation of inflammatory factors to ameliorate bone metabolism

Chronic inflammation is closely associated with bone metabolic imbalance, and the deficiency of key inflammatory cytokines can significantly suppress bone loss. IL-1, IL-6, and TNF-*α*, highly inflammatory cytokines produced mainly by immune macrophages and bone marrow cells, influence bone metabolism by activating various signaling pathways in osteoclasts (136). Numerous studies have shown that coumarins exert notable anti-inflammatory effects in alleviating OP progression through multiple pathways. An *in vitro* study demonstrated that Urolithin A (5–10 μM, 48–96 h) inhibited the expression of IL-6 and TNF-*α* in osteoclasts, attenuates the NF-κB/NLRP3 signaling pathway, and suppresses the secretion of IL-1β and IL-18, thereby inhibiting the formation of osteoclast inflammatory and pyroptotic factors (64). Psoralidin (1–30 μM, 48 h) suppressed the expression of inflammatory factors TNF-*α* and IL-6, blocks RANKL/RANK signaling, and promotes OPG generation to mitigate bone resorption (123). Fraxetin (10-20 μM, 12-48 h) inhibited TNF-α and IL-1β expression levels in MG-63 cells and prevents osteoporosis by reducing inflammatory cell activity and blocking apoptosis (137). Boddu et al. (138) found that Orcinol (2.5–10 μM, 5 days) dose-dependently reduced TNF-*α* expression and inhibited the NF-κB pathway and RANKL-induced osteoclast differentiation and maturation. Furthermore, Si et al. found that in a primary animal model of osteoporosis (OP), oral administration of Osthole (once daily, 4 g/kg for 4 weeks) increased femoral bone mineral density (BMD) in ovariectomized (OVX) rats and improved bone metabolism by suppressing the expression levels of interleukin-6 (IL-6), tumor necrosis factor-alpha (TNF-α), and Ca^2+^ (139). In summary, the inflammatory microenvironment plays a critical role in the regulation of bone metabolic homeostasis. Coumarins may exert potential effects on alleviating bone resorption and protecting the bone microstructure in animal models of OP by inhibiting pro-inflammatory factors and modulating osteoclast differentiation and Ca^2+^ levels. The specific mechanisms of coumarins in different OP models are summarized in Supplementary Table S11.

### 3.5 Mechanisms of coumarin in rheumatoid arthritis

#### 3.5.1 Overview of rheumatoid arthritis

RA is a chronic immune-mediated Musculoskeletal Disease characterized by abnormal vascular formation in joint tissues and persistent synovial hyperplasia, leading to pain, swelling, and functional impairment in multiple joints (140). Infiltration of innate immune cells and activated inflammatory cytokines affects local joint lesions, progressively promoting hyperplasia of adjacent synovial tissues and degradation of the cartilage matrix, thereby contributing to inflammatory joint destruction and damage (141). The onset of RA is closely associated with genetic predisposition and environmental factors. For many years, traditional Chinese medicine has been shown to regulate joint and synovial injury in RA animal models through multi-target and multi-pathway mechanisms, exerting significant modulatory effects on immune-inflammatory cells influenced by genetic variations (142, 143). This study examined the anti-inflammatory and antioxidant effects of coumarins in the context of RA, highlighting their potential and advantages in improving RA prognosis.

#### 3.5.2 Effects of coumarins on joints in animal models of rheumatoid arthritis

The pathogenesis of RA is closely linked to the invasion of inflammatory factors, tumor-like proliferation of synovial tissue, and abnormal vascular proliferation, with pathological manifestations including joint bone erosion and swelling (144). Widely used animal models for RA include collagen-induced arthritis (CIA) and adjuvant-induced arthritis (AIA) (145). The quantitative assessment of joint swelling and tenderness serves as an objective structural indicator for systematically evaluating and monitoring joint damage during RA progression (146). Isopsoralen administered via intraperitoneal injection (once every two days, 5–20 mg/kg for 3 weeks) reduced paw thickness and arthritis score in CIA mice, ameliorated ankle joint injury, increased BV/TV, BMD, Tb. N, and Tb.Th levels, and decreased Tb. Sp levels, thereby alleviating RA-induced bone destruction (147). Urtica dentata Hand administered orally (once daily, 20–60 mg/kg for 4 weeks) dose-dependently reduced RA arthritis score and edema, increased the synthesis of CD4^+^ and CD25^+^ regulatory T cells (Tregs), and protected joint tissue from inflammatory cytokine damage (148). Debasis et al. found that intraperitoneal injection of 8-methoxy-chromen-2-one (MCO) (once daily, 2–20 mg/kg for 3 weeks) restored joint injury in CIA rats and reduced joint pain and ankle arthritis score in RA rats as assessed by the open field test behavioral study (149). Scopolin, a natural coumarin component found in the stems of Dan Tai (Scopolia japonica), has been shown to possess potential anti-RA and autoimmune disease effects (150). Pan et al. (151) found that intraperitoneal administration of a high dose of Scopolin (once daily, 25–100 mg/kg for 9 days) reduced neovascularization and joint swelling in synovial tissue in rats, exhibiting nearly restored normal joint structure. Daphnetin (once daily, 2.25–4.5 mg/kg for 3 weeks) inhibited inflammatory cell infiltration and synovial hyperplasia, and reduced arthritis score and joint swelling to alleviate joint injury in rats (152). Lucinéia et al. (153) demonstrated that the 3-phenylcoumarin derivative 6,7-dihydroxy-3-[3′,4′-methylenedioxyphenyl]-coumarin administered via intra-articular injection (1.5 mg/kg for 6 h) inhibited inflammatory cell infiltration in the synovium of RA rats, reducing joint edema and injury. Shirani et al. (154) found that Auraptene (once daily, 16–64 mM/kg for 2 weeks) and Umbelliprenin (once daily, 16–64 mM/kg for 2 weeks), natural coumarins present in plants of the Apiaceae family, when administered orally, significantly reduced joint thickness and paw swelling in CFA-induced rats compared with indomethacin and prednisolone treatment, exhibiting significant anti-inflammatory potential. Glytabastan B (once daily, 12.5–25 mg/kg for 4 weeks) administered via intraperitoneal injection not only reduced paw swelling and arthritis score but also exerted protective effects against bone erosion and destruction (155). Therefore, different coumarin compounds, including Isopsoralen, Scopolin, Auraptene, and Umbelliprenin, may enhance the alleviation of joint swelling and pain by reducing inflammatory cytokine levels and inflammatory immune cell infiltration, exhibiting superior anti-synovial hyperplasia and joint-protective effects compared with conventional drugs in RA models.

#### 3.5.3 Cellular and molecular mechanisms of coumarins in the regulation of rheumatoid arthritis

##### 3.5.3.1 Regulation of inflammatory factors in joint destruction

The invasion of immune cytokines within the joint cavity—such as dendritic cells, monocytes, and helper T cells—along with a highly hypoxic environment, creates a pathogenic microenvironment for the formation of RA synovial pannus (156). Inflammatory chemokines recruit immune cells into the synovial cavity by stimulating vascular endothelial cells, leading to the accumulation of activated T cells and B cells in fibroblast-like synoviocytes (FLS) and the secretion of inflammatory cytokines that exacerbate joint damage (157). Type A synovial macrophages and FLS release type B cells under inflammatory signaling, producing inflammatory and pro-angiogenic factors (158). The persistent growth of inflammatory pannus facilitates the migration of leukocytes from joint tissues to bone, forming a bridge for inflammatory damage between adjacent cartilage and synovium (159). In recent years, coumarin compounds such as columbianadin, umbelliferone, daphnetin, and scopoletin have shown significant potential in modulating inflammatory cell invasion and excessive immune responses in RA FLS. Columbianadin (12.5–50 μM, 24–48 h) inhibited TNF-*α*-induced invasion of inflammatory factors in MH7A cells, reduced the expression of MMP2 and MMP-9 to suppress synovial cell degeneration, and improved synovial cell adhesion and migration (160). Enhanced VAV2 phosphorylation activity promotes Rac-1 expression, inhibits synovial cell apoptosis and autophagy, and facilitates abnormal FLS growth. Umbelliferone (10–40 μM, 24–48 h) reduced the production of pro-inflammatory cytokines (IL-1, IL-6, IL-8) in FLS and inhibited the abnormal invasion and inflammatory response of RA FLS by decreasing Wnt1 and GSK-3 protein levels (161). Chen et al. (162) and Mao et al. (163) found that Daphnetin (40 μg/mL, 48–72 h) activated the caspase-induced cell death pathway in FLS of CIA rats, increased the gene and protein expression of FasL, caspase-9, and Cyt-c, as well as the Bax/Bcl-2 ratio, thereby promoting synovial cell apoptosis and alleviating FLS inflammation. The enhanced apoptotic activity is an important mechanism by which Daphnetin significantly inhibits FLS hyperproliferation and reduces inflammatory migration in a dose-dependent manner. Imperatorin (0.713–71.3 μM, 48 h) not only significantly reduced collagenase-induced joint inflammation but also promoted apoptosis to attenuate synovial hyperplasia and pannus formation (164). 3R-(4′-hydroxyl-3’-O-*β*-D-glucopyranosyl phenyl)-dihydro isocoumarin (GDC) (3–30 μM, 24 h) inhibited the expression of CNTF, IFN-*γ*, IL-4, and TIMP-1 in lipopolysaccharide (LPS)-induced FLS, reducing inflammatory cytokine levels and ameliorating abnormal FLS proliferation (165). Another study showed that Scopoletin (15–60 μM, 24 h) inhibited the elevation of IL-6 expression in IL-1β-stimulated FLS and significantly affected the MAPK/PKC/CREB signaling pathway in a dose-dependent manner, thereby inhibiting angiogenesis and exerting anti-RA effects (166).

Stimulation of FLS and immune cells in RA joints leads to continuous production of inflammatory mediators such as IL-1β, TNF-*α*, and PGE2, resulting in increased osteoclast numbers and bone resorption. Matrix metalloproteinases (MMPs) degrade extracellular matrix components in RA, cleaving the structural integrity of joint cartilage and bone tissue (167). Additionally, MMPs are selectively cleaved by FLS, osteoclasts (OC), endothelial cells (EC), and neutrophils in RA joints, participating in cartilage inflammation and degradative enzyme synthesis through chemokine modification, growth factor transcription, and proenzyme activation (168). Inflammatory signaling pathways can also directly catalyze joint inflammation and damage in RA immune diseases via MMPs (169). Glytabastan B (3–6 μM, 24 h) inhibited the elevation of MMP expression in IL-1β-induced SW982 human synovial cells, attenuated RANKL-induced osteoclast expression, and alleviated articular cartilage matrix degradation and bone resorption (155). Dolichosin A (10–20 μM, 48–96 h) not only significantly reduced IL-1β-induced synovial inflammation in SW982 synovial cells, including the expression of TNF-*α*, IL-6, and COX-2, but also suppressed MMP-3 and RANKL levels to reduce osteoclast number and TRAP activity (170). Geum et al. found that Cinnamomulactone (0.1 μM, 24 h) inhibited TNF*α*-stimulated gene expression of MMP-3 and MMP-1 in FLS, and alleviated joint injury and destruction in RA rats (171). Umbelliferone (10–40 μM, 24–48 h) alleviated adjuvant-induced joint injury and synovial inflammation in rats by inhibiting MMP-2 and MMP-9 activity in MH7A cells (161). Kim et al. (172) demonstrated that Decursin (20–60 μM, 24 h) inhibited LPS-stimulated MMP-9 expression in THP-1 cells in a dose-dependent manner, reduced the inflammatory levels of IL-8, MCP-1, IL-1β, and TNF-α, and alleviated FLS inflammatory injury. In summary, these coumarin compounds reduce the production of immune-related inflammatory factors such as IL-1, TNF-α, and COX-2, inhibit the synthesis of genes and proteins involved in articular extracellular matrix (ECM) degradation, including MMP-2, MMP-3, and MMP-9, and ameliorate RA-induced synovial and bone degeneration. Furthermore, compounds such as Columbianadin, Umbelliferone, and Scopoletin may regulate inflammatory signaling pathways, potentially affecting downstream inflammatory factors and bone matrix degeneration. These findings reveal the potential of coumarins in regulating the migration and invasion of RA FLS.

The anti-inflammatory therapeutic effects of coumarin compounds, including Columbianadin, Daphnetin, Osthole, and 4-methylesculetin, have also been demonstrated in animal models of rheumatoid arthritis. Columbianadin (once daily, 10–40 mg/kg for 6 weeks) administered by gavage inhibited the synoviocyte-mediated VAV2/Rac-1 signaling pathway and ameliorated cartilage necrosis, calcification, and synovial inflammation in CIA mice (160). Gao et al., Tu et al., and Shu et al. demonstrated (173–175) that Daphnetin (once daily, 2.25–4.5 mg/kg for 3 weeks) administered orally reduced the production of serum IL-1, TNF-*α*, and macrophage migration inhibitory factor (MIF) in rats, thereby ameliorating synovitis and joint destruction caused by pro-inflammatory cytokines. These studies collectively indicate that Daphnetin may inhibit the overactivation of Th-type immune factors to ameliorate FLS inflammation. Hemshekhar et al. (176) found that oral administration of 4-methylesculetin (once daily, 25–50 mg/kg for 2 weeks) inhibited serum IL-6 and COX-2 levels, reduced MMP-13 and MMP-9 levels, and promoted the synthesis of cathepsin-D and tartrate-resistant acid phosphatase (TRAP) in joint tissues, thereby alleviating joint injury and bone resorption in rats. Oral administration of Osthole (once daily, 20–40 mg/kg for 3 weeks) reduced serum TNF-*α* and IL-1β levels, alleviated the arthritis index, toe volume, and toe swelling rate in rats, and also inhibited the protein levels of p-NF-κB, p-IKK, p-p38, p-ERK, and p-JNK, as well as the activation and release of the NLRP3 inflammasome, thereby alleviating synovitis and joint deformity in rats (177). Oral administration of Umbelliferone (once daily, 10–40 mg/kg for 4 weeks) inhibited NF-κB and VEGF expression, reduced the expression of COX-2, PGE2, TNF-α, and IL-17 in the joints of RA rats, and downregulated the RANKL/RANK/OPG ratio to inhibit osteoclast-induced joint injury (178). In summary, these findings elucidate the anti-inflammatory mechanisms of coumarin compounds in RA from the perspective of related immune cells and provide new preclinical evidence for the therapeutic research targeting synovitis and cartilage destruction in RA.

##### 3.5.3.2 Regulation of oxidative factors in joint destruction

Infiltration of mononuclear immune cells, neutrophils, and macrophages into RA joint tissues can trigger ROS-mediated oxidative reactions and inflammation. Excessive ROS induces the generation of inflammatory factors, leading to synovial inflammation and fibrosis (179). ROS is mediated by the mitochondrial NADPH oxidase system, with NADPH oxidase 4 (NOX4) inducing a significant increase in oxygen consumption and superoxide (O^₂-^) generation (180). ROS-catalyzed superoxide dismutase promotes the recruitment of inflammatory factors such as TNF-*α*, IL-17, and iNOS in RA FLS, leading to invasive migration and autoimmune bone damage in RA (181). 3R-(4′-hydroxyl-3’-O-*β*-D-glucopyranosyl phenyl)-dihydro isocoumarin (GDC) (3–30 μM, 24 h) regulated mitochondrial activity, reduced iNOS levels, and blocked the NF-κB and MAPK signaling pathways to alleviate FLS hyperplasia and joint destruction (166). Osthole (25–50 μM, 48 h) activated AMPK expression, ameliorated abnormal mitochondrial activity in FLS, reduced ROS levels to restore mitochondrial homeostasis, and simultaneously suppressed the gene expression of Mfn1, Mfn2, Opa1, and MSTO1 to promote synovial cell death (177). Osthole (50 μM, 48 h) also increased miR-1224-3p levels to reduce IL-6 and IL-1β expression, and inhibited AGO1 levels to alleviate abnormal cell proliferation in human primary synovial fibroblasts (HUM-iCell-s010) (182). Oxypeucedanin hydrate (15–60 μM, 24 h) inhibited the elevation of iNOS, COX-2, IL-1β, and TNF-*α* expression in LPS-induced RAW264.7 macrophages and blocked the transcription of the NF-κB/MAPK inflammatory pathway in CIA rats by reducing ROS production (183). Therefore, these findings indicate that Oxypeucedanin hydrate, Osthole, and coumarin-based chalcones 3a–f may inhibit mitochondrial oxidative stress by promoting antioxidant capacity and anti-inflammatory cascades, consequently affecting downstream cell death or pyroptosis pathways to improve oxidative stress and pathological progression in RA.

##### 3.5.3.3 Regulation of MAPK, PI3K/AKT, and NF-κB signaling pathways to ameliorate joint destruction

**Figure 5:**
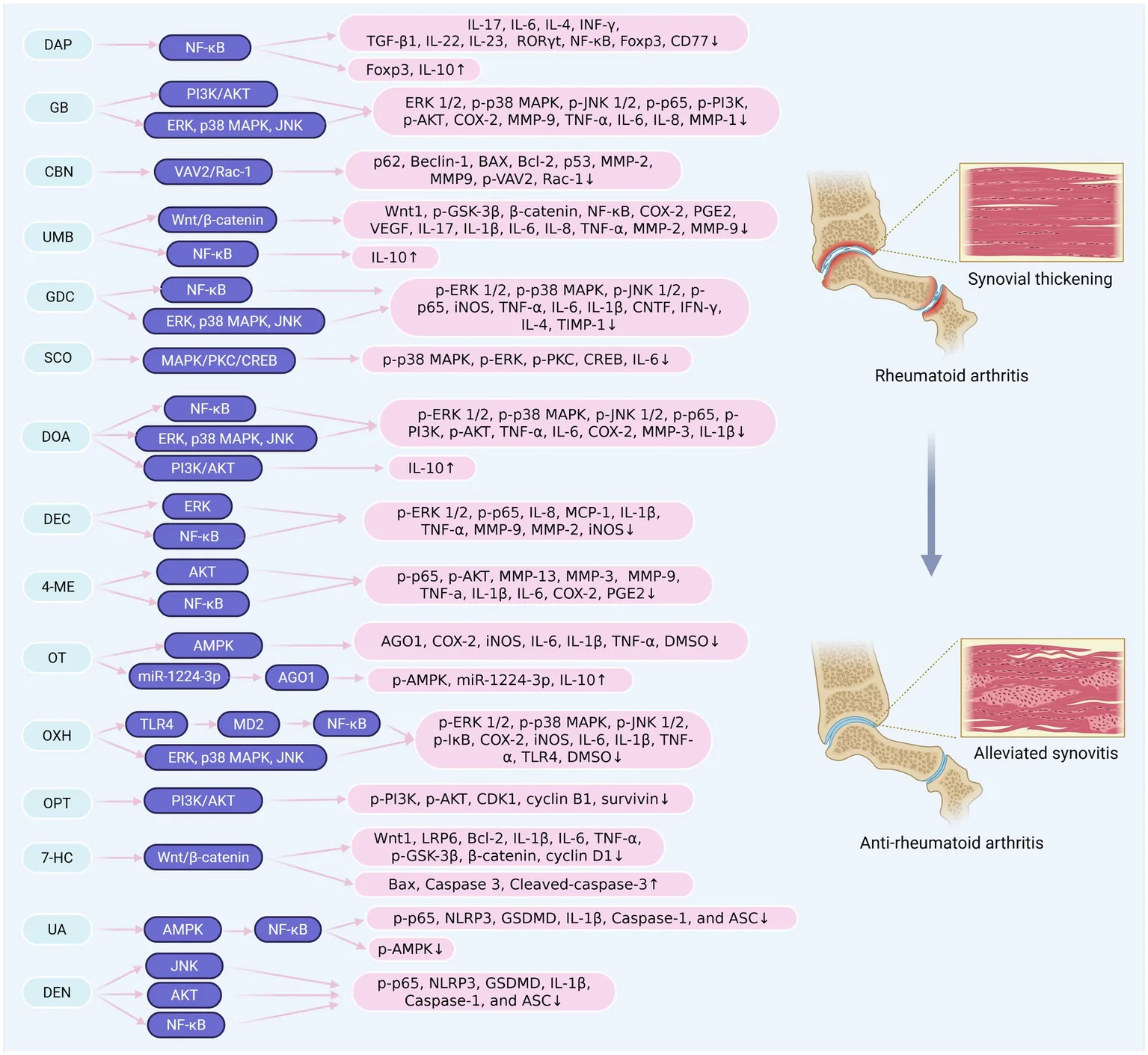
Immunomodulatory and anti-inflammatory mechanisms of coumarins in rheumatoid arthritis (RA) through associated signaling pathways. DAP, daphnetin; GB, glytabastan B; CBN, columbianadin; UMB, umbelliferone; GDC, 3R-(4′-hydroxyl-3’-O-β-D-glucopyranosyl phenyl)-dihydro isocoumarin; SCO, scopoletin; DOA, dolichosin A; DEC, decursin; 4-ME, 4-methylesculetin; OT, osthole; OXH, oxypeucedanin hydrate; OPT, oxyimperatorin; 7-HC, 7-hydroxycoumarin; UA, urolithin A; DEN, dendrocoumarin. ↑, promotion; ↓, inhibition.

FLS inflammation, pannus formation, and tissue fibrosis in RA progression are closely associated with signaling pathways such as MAPK, PI3K/Akt, TLR4, and AMPK-coupled pathways (184). These pathways directly or indirectly regulate the phosphorylation activity of NF-κB, thereby transcribing and activating downstream pro-inflammatory factors, chemokines, and immune regulatory receptors, and even modulating NFATc1 to influence osteoclastogenesis in RA joints (185). Coumarin compounds, such as Osthole and Umbelliferone, have shown significant potential in regulating the MAPK and NF-κB signaling pathways for the treatment of RA animal models, delaying synovial inflammation and joint destruction (177, 178). In *in vitro* studies, Oxyimperatorin (50–100 μg/mL, 24–48 h) targeted the PI3K/AKT pathway to inhibit DNA replication and the expression level of cyclin-dependent kinase 1 (CDK1), thereby disrupting the cell cycle of FLS and promoting cell death to alleviate the invasion of inflammatory FLS cells (186). Li et al. (178, 187) found that Umbelliferone (10–40 μM, 24–48 h) and 7-Hydroxycoumarin (20–80 μM, 48 h) reduced the expression and activity of Wnt1, GSK-3, and Ser9 proteins by blocking the Wnt/*β*-catenin signaling pathway, thereby inhibiting the transcription and invasion of inflammatory factors in FLS. Urolithin A (10–40 μM, 12–24 h) activated AMPK and blocked the NF-κB signaling pathway to suppress inflammatory changes in FLS cells, and reduced the protein level of the NLRP3 inflammasome to inhibit pyroptosis (188). Thus, coumarin compounds may alleviate RA progression by inhibiting the NF-κB signaling pathway and regulating downstream inflammatory pathways involved in pyroptosis. Glytabastan B (3–6 μM, 24 h) and Dolichosin A (10–20 μM, 48–96 h) blocked MAPK and PI3K/AKT signaling to inhibit joint inflammation and reduced RANKL-induced osteoclastogenesis (155, 170). Oxypeucedanin hydrate (15–60 μM, 24 h) increased anti-inflammatory activity in RAW264.7 macrophages by inhibiting the TLR4/MD2 and NF-κB/MAPK signaling pathways (183). Dendrocoumarin (10 μM, 24 h) inhibited the NF-κB, Akt, and JNK signaling pathways, reducing the expression levels of IL-2, IL-6, IL-8, and IFN-*γ* to suppress MH7A cell inflammation (189). Therefore, coumarin compounds may inhibit inflammatory factors and pyroptosis by blocking the activation of NF-κB, MAPK, PI3K/AKT, AMPK, and Wnt/*β*-catenin signaling pathways, thereby alleviating joint destruction and synovial pathological fibrosis, highlighting their potential as an intervention strategy for RA (Figure 5). The effects of coumarins on RA in *in vivo* and *in vitro* studies are summarized in Supplementary Table S12.

### 3.6 Mechanisms of coumarin in osteoarthritis

#### 3.6.1 Overview of osteoarthritis

Osteoarthritis (OA) is an age-related degenerative chronic Musculoskeletal Disease characterized by progressive joint pain, osteophyte formation, and joint dysfunction. Inflammatory factors derived from macrophages and chemokines are primary contributors to OA pathogenesis, activating pro-inflammatory and catabolic responses in cartilage-resident cells, leading to subchondral bone remodeling, degradation, and synovitis (190). Current OA inflammation models, including chemical induction (e.g., sodium monoiodoacetate injection) and surgical models (e.g., collateral ligament transection and medial meniscectomy), induce joint instability and injury, providing a basis for exploring drug mechanisms and potential clinical strategies for OA (191). Given the multi-target anti-inflammatory mechanisms of coumarins, summarizing their efficacy and molecular regulatory mechanisms in OA treatment is of significant importance.

#### 3.6.2 Effects of coumarins on cartilage quality in animal models

Cartilage damage in OA is manifested by alterations in subchondral and trabecular bone properties, including reduced subchondral bone thickness, changes in bone volume, and decreased [BMD (192)]. As the largest joint in the human body, the knee exhibits a lower elastic modulus in epiphyseal bone and reduced proximal tibial hardness with OA progression. Furthermore, low-quality proximal tibial hardness can lead to severe joint deformation, closely associated with knee pain and dysfunction (193). Psoralen (once daily, 0.1 mg/kg for 5 administrations) administered via intra-articular injection increased estrogen levels in OA rabbits, elevated BMD, Tb.Th, and Tb. N, decreased Tb. Sp, and alleviated cartilage damage and cartilage thickness (194). Wan Nurfarahin et al. (195) found that oral administration of Scopoletin (once daily, 200–400 mg/kg for 4 weeks) significantly reduced cartilage erosion and subchondral bone sclerosis in rats, and Micro-CT revealed increased subchondral bone structural density in the tibia following Scopoletin treatment. Esculin (once daily, 40 mg/kg for 4 weeks) alleviated superficial cartilage degeneration and osteophyte formation in DMM mice, and ameliorated OA-induced joint injury caused by proteoglycan loss and cartilage erosion (196). Tsuchiya et al. (197) found that 4-methylumbelliferone mixed into the diet of DMM mice at 5% (w/w) reduced the OARSI score in the joints, decreased osteophyte formation and joint pain after 2 weeks. Nodakenin administered orally (once daily, 10 mg/kg for 8 weeks) increased subchondral bone mass and trabecular number in DMM mice, reduced trabecular separation, and ameliorated articular cartilage degeneration (198). Urolithin B administered via intraperitoneal injection (once every 2 days, 50 mg/kg for 8 weeks) improved the OARSI score in the knee joint of ACLT mice, reduced MMP-13 levels, increased COL2a expression, and ameliorated joint space narrowing and inhibited osteophyte formation (199). Oral administration of Osthole (once daily, 2–5 mg/kg for 4 weeks) significantly increased cartilage proteoglycan content, improved walking distance, and reduced fall latency in OA mice. It also inhibited the activity of the NF-κB and HIF-2*α* pathways in mouse chondrocytes, reduced the inflammatory expression of COX-2 and RUNX2, and affected MMP-13, Syndecan IV, and ADAMTS-5 levels to alleviate osteoarthritis degeneration (200). These findings reveal that coumarin compounds such as Psoralen, Scopoletin, Esculin, Nodakenin, 4-methylumbelliferone, and Urolithin B may exert multiple mechanisms in ameliorating subchondral bone degeneration, including increasing trabecular number, reducing osteophyte formation, and enhancing cartilage matrix, demonstrating their potential to ameliorate OA degeneration.

#### 3.6.3 Cellular and molecular mechanisms of coumarins in the regulation of osteoarthritis

##### 3.6.3.1 Regulation of inflammatory factors to ameliorate joint injury

Inflammation is a fundamental mechanism in OA pathophysiology, and excessive mechanical stress, joint injury, or incorrect biomechanics also serve as important catalysts for articular cartilage degeneration. Pro-inflammatory factors (e.g., IL-1β and TNF-*α*) produced by autocrine and paracrine cells recruit macrophages to synthesize NO and eicosanoids (e.g., PGE2 and leukotrienes), inducing the activation of catabolic pathways in the cartilage matrix and joint degeneration (201). Byakangelicin inhibited IL-1β-mediated expression of iNOS, COX-2, TNF-α, and IL-6 in mouse chondrocytes, alleviating joint inflammation, while promoting collagen II and aggrecan expression and reducing ADAMTS-5 and MMP levels to prevent cartilage ECM degradation (202). Inflammatory factors stimulate the production of matrix-degrading enzymes such as MMPs, particularly key enzymes including MMP-3, MMP-13, and ADAMTS, which impede the production of proteoglycans and type II collagen in the cartilage ECM, leading to structural damage and matrix degradation (203). Urolithin B (5–10 μM, 48–96 h) inhibited IL-1β-induced production of MMP-3 and MMP-13 in chondrocytes and upregulated the expression of collagen II and aggrecan, thereby alleviating joint degeneration (199). Imperatorin (50 μM, 24–48 h) significantly reduced the production of HIF-1*α*, NLRP3, IL-1β, and IL-18 in LPS-induced synovial fibroblasts and inhibited the expression of TGF-β1, TIMP-1, and VEGF, thereby alleviating OA synovial inflammation and fibrosis (204). Idota et al. (205) found that 4-Methylumbelliferone (0.5–2 μM, 12 h) reduced glycolysis dependence in IL-1β-induced human ACL-derived cells and inhibited the expression of MMP-1, MMP-3, and IL-6, thereby alleviating cartilage matrix degradation. Fraxetin (10–50 μM, 24 h) reduced IL-1β-induced protein expression levels of TNF-α and IL-6 in chondrocytes, promoted collagen II synthesis, and inhibited MMP-13 levels, exerting anti-chondrocyte ECM degradation and anti-inflammatory effects (206). Daphnetin (12–48 ng/mL, 8 weeks) inhibited IL-1β-induced expression of IL-12, MMP-3, MMP-9, and MMP-13 in rabbit chondrocytes and increased IL-10 synthesis to enhance the anti-inflammatory capacity in OA (207). Bergapten (10 μM, 24 h) activated the ANP32A/ATM signaling pathway to inhibit IL-1β-induced expression of IL-6, TNF-α, COX-2, and MMP-13 in rat chondrocytes, maintaining chondrocyte anti-inflammatory capacity and promoting cartilage ECM secretion (208). You et al. (209) demonstrated that Coumestrol (1–10 μM, 21 days) inhibited the expression of MMP-3 and MMP-13 in primary rat chondrocytes to alleviate proteoglycan loss and downregulated iNOS, COX-2, and PGE2 levels to suppress inflammatory cell proliferation. In summary, multiple findings indicate that coumarin compounds such as Byakangelicin, Imperatorin, Fraxetin, and Bergapten alleviate OA synovial inflammation and arthritic injury by inhibiting the release of key pro-inflammatory cytokines, including IL-1β, IL-6, iNOS, and COX-2. Furthermore, coumarin compounds such as Urolithin B, 4-Methylumbelliferone, Daphnetin, and Coumestrol may reduce MMP production by suppressing inflammatory expression, increase proteoglycan synthesis to alleviate cartilage matrix degradation, and promote articular cartilage repair and anti-inflammatory function.

##### 3.6.3.2 Regulation of oxidative factors to ameliorate joint injury

Oxidative stress in OA can shorten telomerase with aging, accelerate chondrocyte senescence, alter the properties of cartilage collagen fibers, and increase collagen brittleness (210). ROS produced by NADPH oxidases (e.g., NOX2) are central to mitochondrial energy metabolism in cartilage, and mitochondrial free radical damage leads to ROS accumulation, affecting chondrocyte growth and cartilage matrix catabolism (211). Esculin (5–20 μM, 24–48 h) reduced IL-1β-induced ROS production in chondrocytes, inhibited COX-2 and iNOS levels, and regulated oxidative stress and inflammation in chondrocytes through the Sirt1 pathway (196). Nodakenin (75 μM, 24 h) inhibited LPS-induced mitochondrial Drp1 phosphorylation expression and excessive ROS activity in chondrocytes, downregulated the expression of COX-2, IL-1β, TNF-*α*, and NLRP3, and suppressed MMP-13 mRNA levels to alleviate cartilage degradation and inflammatory response (198). Psoralidin (5–15 μM, 24–48 h) inhibited IL-1β-induced NO and ROS production in chondrocytes, reduced the expression of caspase-3, caspase-9, MMP-1, and MMP-13, and alleviated chondrocyte apoptosis and degeneration (212). Psoralen (125–150 μM, 24–72 h) also increased the antioxidant capacity of IL-6-induced chondrocytes by activating the Keap1/Nrf2/ARE signaling pathway, and reduced the production of MMP-13 and collagen II to improve the articular cartilage microenvironment (213). Notably, oxidative stress triggers inflammation and cartilage matrix degradation, and mitochondrial damage also causes dysfunction of chondrocyte autophagy. Psoralen is an important compound that maintains the balance of this key mechanism. D’Amico et al. (214) found that Urolithin A (6.25–12 μM, 24 h) improved mitochondrial respiration, promoted the expression of the LC-3 autophagy gene BNIP3, and activated the PINK1-Parkin signaling pathway to enhance autophagy in human chondrocytes, thereby reducing cartilage matrix degeneration and joint inflammation. Isofraxidin (1–50 μM, 24 h) inhibited IL-1*β*-stimulated NO, iNOS, COX-2, and PGE2 expression in human OA chondrocytes to ameliorate inflammation, and suppressed the expression of MMPs, ADAMTS-4, and ADAMTS-5 to alleviate ECM degradation (215). Therefore, coumarins regulate oxidative stress to alleviate OA progression. These compounds may modulate ROS, autophagy, and cell death by ameliorating mitochondrial damage, inhibiting inflammation, and activating the Nrf2 pathway, exerting potential protective effects on the remodeling and degeneration of the OA joint microenvironment.

##### 3.6.3.3 Regulation of NF-κB, MAPK, and Wnt/β-catenin signaling pathways to ameliorate joint injury

NF-κB is a key signaling pathway through which inflammatory factors disrupt cartilage matrix integrity and promote catabolism in OA. Its activation requires excessive mechanical stress and abundant release of pro-inflammatory factors, and it strongly stimulates inflammatory mediators (such as IL-1β, TNF-*α*, and PGE2) in a positive feedback manner (216). The canonical NF-κB pathway involves dimers composed of p65, c-Rel, and p50 subunits, requires the IKK complex (IKKα/β/*γ*) and IκB multimer for dependent negative feedback, and coordinates multiple signaling layers to promote the production of matrix-degrading enzymes and inflammation, exacerbating OA onset and progression (217). Coumarin compounds such as Byakangelicin (0.625–2.5 μM, 24–48 h), Isofraxidin (1–50 μM, 24 h), Hydroxypyridinone-Coumarin (5–50 μM, 48 h), and Wedelolactone (10–50 μM, 24–72 h) exert protective effects on OA chondrocytes by inhibiting the NF-κB signaling pathway, attenuating p65 nuclear translocation and IκBα phosphorylation activity in chondrocytes, suppressing the release of downstream inflammatory factors, promoting aggrecan synthesis, and inhibiting ECM-degrading enzyme levels, thereby alleviating joint inflammation and cartilage degeneration (202, 215, 218, 219). Toll-like receptors (TLRs), as surface-expressed pattern recognition receptor (PRR) families, such as TLR2 and TLR4, bind to their downstream ligand myeloid differentiation primary response 88 (MyD88)/NF-κB, which can activate OA inflammatory factors and chondrocyte degeneration (220). Fraxetin (10–50 μM, 24 h) inhibited IL-1β-induced chondrocyte apoptosis and the release of inflammatory mediators in rat chondrocytes by suppressing the TLR4/MyD88/NF-κB signaling pathway (206). Isofraxidin (5–20 μM, 24–48 h) attenuated TLR4/NF-κB signaling pathway activity by inhibiting the fusion of the TLR4/myeloid differentiation protein-2 (MD-2) complex, reversing LPS-induced ECM degradation and inflammatory release in chondrocytes (221). Therefore, TLR2 and TLR4 play important roles in mediating OA progression by activating the downstream NF-κB pathway. Scoparone (5–20 μM, 24 h) and Urolithin A (3–30 μM, 24–48 h) inhibited IL-1β-induced NO, COX-2, and iNOS expression in human chondrocytes and downregulated MMP-13 and ADAMTS-5 levels to suppress cartilage ECM degradation by affecting the activation of the PI3K/Akt/NF-κB pathway (222, 223). Notably, the PI3K/Akt signaling pathway and the downstream NF-κB pathway have synergistic effects in promoting OA inflammatory responses, and coumarin compounds can block the PI3K/Akt pathway to influence the phosphorylation expression of the NF-κB pathway. Esculin (5–20 μM, 24–48 h) inhibited NF-κB transcription by activating the Sirt1 pathway, blocking IL-1β-induced p65 and IκB phosphorylation expression in human chondrocytes, and suppressing cartilage ECM degradation and inflammatory factor levels (196). Dicoumarol (30 μM, 24 h) inhibited LPS-induced NLRP3 inflammasome activation in rat synovial FLS, reduced IL-1β and IL-18 levels, and promoted TIMP1 and COL1A1 expression to alleviate OA synovial inflammation and fibrosis, but did not significantly affect NF-κB signaling (224). Specifically, OA inflammatory cells induced by the combination of LPS and ATP strongly promoted the nuclear translocation of NF-κB p65. Due to the differences in the chemical structure and pharmacodynamics of Dicoumarol, it did not intervene in NF-κB factors in this study but inhibited downstream inflammasome expression, demonstrating the intensive relationship between NF-κB and the NLRP3 inflammasome in OA. In summary, Byakangelicin, Wedelolactone, Isofraxidin, Scoparone, Urolithin A, Osthole, and Dicoumarol may alleviate OA progression through the classical NF-κB signaling pathway, while the TLR, PI3K/Akt, and Sirt1 pathways play important roles in influencing downstream NF-κB regulation, exhibiting unique mechanisms in regulating cell death.

MAPKs are serine/threonine protein kinases readily activated by extracellular signals, physical stimuli, and inflammatory factors (e.g., IL-1β and IL-6), regulating chondrocyte transcription, proliferation, differentiation, and senescence (225). The MAPK signaling pathway includes ERK, JNK, and p38 subtypes. Upon activation, they bind to downstream transcription factors to promote OA inflammatory responses, induce loss of cartilage matrix proteoglycans and release of degrading enzymes, and cause joint destruction and chondrocyte degeneration (226). Recent studies have found that coumarins and their metabolites play important regulatory roles in blocking MAPK signaling pathways. Ahmad et al. (227) found that Imperatorin (10–50 μM, 24 h) reduced iNOS and NO expression in IL-1*β*-induced human OA chondrocytes by blocking ERK–MAPK/AP1 signaling pathway activity, thereby alleviating OA inflammation. Daphnetin (12–48 ng/mL, 8 weeks) inhibited the MAPK signaling pathway in IL-1*β*-induced primary rabbit chondrocytes, downregulated Caspase-3 expression, and promoted Bcl-2 expression to attenuate chondrocyte apoptosis (207). Xanthotoxin (50–100 μM, 24–72 h) increased the expression of histone deacetylase 4 (HDAC4) in MSCs by blocking p38 MAPK signaling pathway and its phosphorylation, and inhibited Runx2 and MMP-13 levels in chondrocyte hypertrophic cells to maintain normal chondrocyte phenotype and function (228). Therefore, these coumarin compounds may alleviate OA joint degeneration by regulating the MAPK signaling pathway to alter chondrocyte degeneration and the release of inflammatory factors.

**Figure 6:**
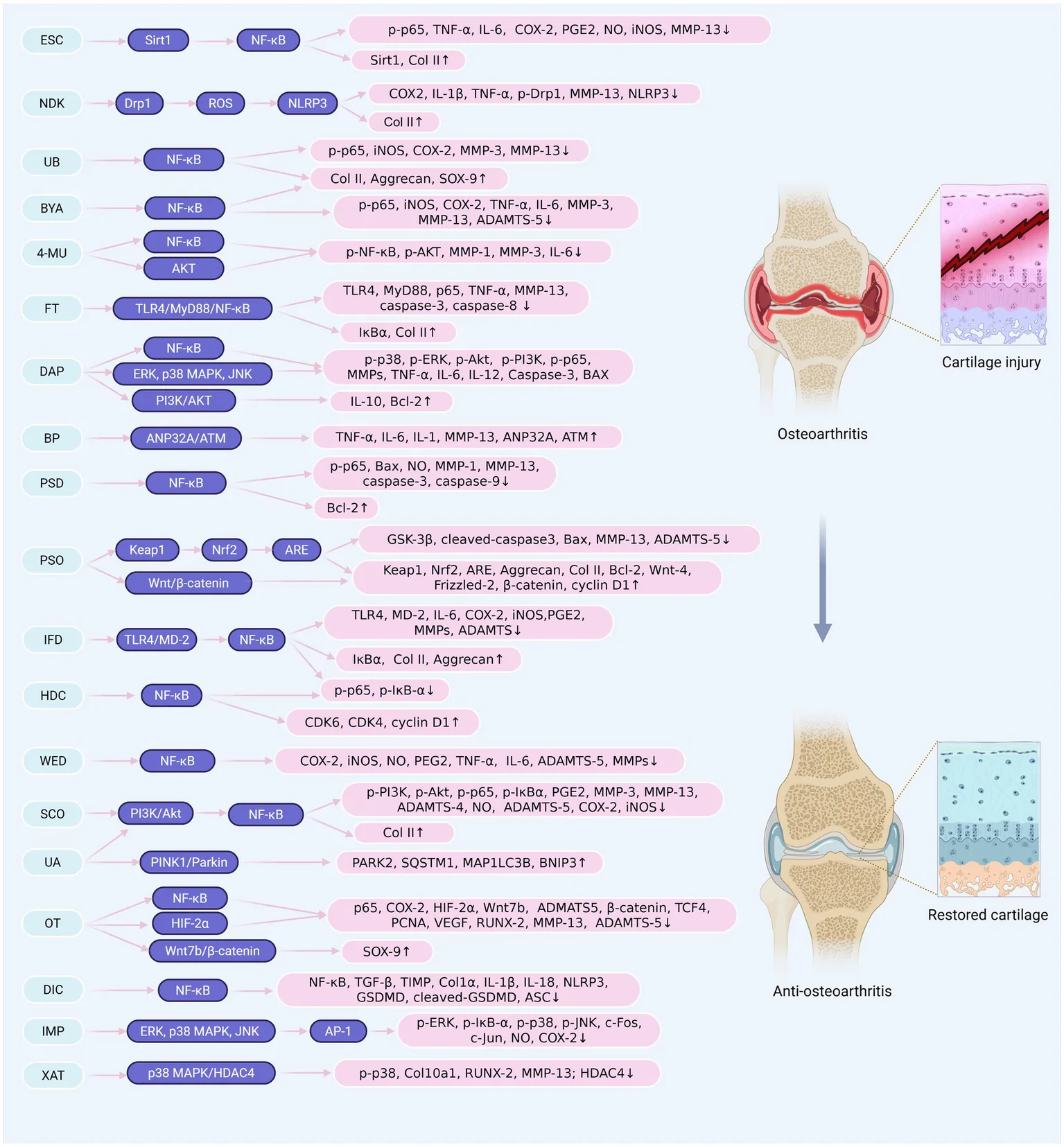
Anti-inflammatory mechanisms and cartilage-protective effects of coumarins in osteoarthritis via related signaling pathways. ESC, esculin; NDK, nodakenin; UB, urolithin B; BYA, byakangelicin; 4-MU, 4-Methylumbelliferone; FT, fraxetin; DAP, daphnetin; BP, bergapten; PSD, psoralidin; PSO, psoralen; IFD, isofraxidin; HDC, hydroxypyridinone-coumarin; WED, wedelolactone; SCO, scoparone; UA, urolithin A; OT, osthole; DIC, dicoumarol; IMP, imperatorin; XAT, xanthotoxin; PRL, psoralen. ↑, promotion; ↓, inhibition.

Canonical Wnt pathway activation by *β*-catenin can drive skeletal mesenchymal cells toward chondrocyte differentiation or osteogenic transition, while genetic mutations or overexpression lead to chondrocyte pathology and matrix damage. The Wnt/*β*-catenin signaling pathway plays a fundamental role in OA cartilage remodeling and the production of catabolic degrading enzymes (229). Psoralen (10^−7^–10^−5^ mol/L, 24–72 h) activated the Wnt/β-catenin signaling pathway and increased the protein and gene expression of Wnt4, Frizzled-2, Col-II, and cyclin D1 in rat chondrocytes in a dose- and time-dependent manner, thereby promoting chondrocyte proliferation (230). Previous studies have demonstrated that loss of Wnt4 predisposes to spontaneous OA joint and decreased cortical bone quality with aging (231). Osthole (6.25–25 μM, 24 h) inhibited the Wnt7b/*β*-catenin signaling pathway and reduced the expression of MMP13, ADAMTS-5, and MMP9 in IL-1β-induced chondrocytes in a dose-dependent manner, while increasing Col-II levels to suppress chondrocyte catabolism and degeneration (232). Specifically, the Wnt7 signaling pathway plays an important role in OA joint destruction and pathological progression (233). Therefore, the Wnt/β-catenin signaling pathway exhibits different mechanisms in the treatment of OA with coumarin compounds, and various Wnt subtypes show significant differences in regulating chondrocyte proliferation and catabolism of the inflammatory matrix. In summary, coumarins may exert significant effects on the differentiation mechanisms in OA treatment by regulating the NF-κB, MAPK, and Wnt/β-catenin pathways, promoting cartilage matrix collagen synthesis, inhibiting OA cell death, and modulating inflammation to exert chondroprotective potential (Figure 6). The effects of coumarins on OA in *in vivo* and *in vitro* studies are summarized in Supplementary Table S13.

### 3.7 Mechanisms of coumarin in osteosarcoma

#### 3.7.1 Overview of osteosarcoma

Osteosarcoma (OS) is a primary malignant bone tumor derived from mesenchymal stem cells, predominantly occurring in adolescent patients aged 15–19 years (234). OS sarcoma cells are characterized by the production of osteoid or immature bone and typically develop in the metaphyses of long bones, such as around the knee joint. The etiology of OS is complex, with most pathogenesis and genetic factors closely associated with complex syndromes such as Li-Fraumeni syndrome, retinoblastoma, and Bloom syndrome (235, 236). Reduced immune surveillance in the tumor microenvironment (TME), abnormal unlimited proliferation of tumor cells, and rapid metastasis contribute to OS development. Thus, immune cell regulation represents a key target for anti-tumor immunotherapy in OS progression (237). Coumarins, leveraging their anti-inflammatory and antioxidant pharmacological properties, exert immunomodulatory effects on tumor progression both *in vivo* and *in vitro*, highlighting their significant potential in OS therapy.

#### 3.7.2 Effects of coumarins in regulating osteosarcoma cell migration

Circulating tumor cells (CTCs) can escape from the primary lesion into the circulatory system in small numbers, leading to distant metastasis or recurrence of OS (238). Approximately 15–20% of OS patients present with metastasis at diagnosis, primarily to the lungs (85%) and bones (10%) (234). The development and metastasis of OS involve invasion of the extracellular host matrix, evasion of the host immune system, arrest and invasion of target organs, and adhesion and survival within target organs (239). Specific genetic factors and metastatic pathways in OS, such as Wnt/*β*-catenin, Notch, Fas/Fas ligand, and VEGF pathways, facilitate tumor migration and growth in target cells. In an animal study, Arai et al. found that intraperitoneal injection of 4-methylumbelliferone (10 mg/body for 2 weeks) inhibited LM8 tumor growth in mice, as evidenced by a 49% reduction in tumor wet weight and a significant 75% reduction in metastatic lung lesions (240). Notably, 4-methylumbelliferone significantly inhibited the surrounding stromal tissue and perivascular regions *in vivo*, markedly suppressed lung metastasis, and may inhibit the growth and metastasis of OS cells through multiple mechanisms, exhibiting inhibitory effects on tumor cell proliferation. 4-methylumbelliferone (1 mM, 72 h) inhibited hyaluronic acid (HA) secretion, reduced the formation of the functional tumor cell ECM, and significantly suppressed (85%) the probability of OS lung metastasis, but did not significantly affect cell proliferation (240). The biological interaction between exogenous HA (free HA) and endogenous HA (cell-associated HA) may differ. In this study, HA was exogenous, and further research is needed to clarify the antiproliferative mechanism of 4-methylumbelliferone against OS. Ahmadi et al. (241) found in an *in vitro* study that coumarin galbanic acid (25–100 μM, 24–48 h) reduced MMP-2 and MMP-9 enzymatic activity in MG-63 cells, and at a concentration of 100 μM, significantly inhibited cell invasion and adhesion, thereby impeding OS metastasis. Ahmadi et al. (242) also found that Urolithin A (75 μM, 24–48 h) significantly reduced the migration ability of human MG-63 cells by inhibiting MMP-2 and MMP-9 enzymatic activity, effectively suppressing MG-63 cell metastasis and invasion. Notably, MMPs play an important role in OS metastasis. By promoting the degradation of the tumor cell ECM, MMPs increase OS cell dissemination, which is closely associated with an increased incidence of lung metastasis in OS patients (243). Hosseini et al. (244) demonstrated that 7-Geranyloxycoumarin (25–100 μM, 24–48 h) reduced MMP-2 and MMP-9 levels in OS cells, decreased tumor cell invasion and adhesion, and blocked OS cell migration and metastasis by regulating JAK1 and JAK2 expression, without significantly affecting apoptosis. Kimura et al. (245) found that Fraxetin (10–100 μM), one of the dihydroxycoumarins (10–100 μM, 24–72 h), significantly inhibited TGF-β1 and VEGF expression to impede OS cell metastasis to the lungs or liver, and reduced the expression of cyclin D1, cyclin-dependent kinase (CDK) 4, and MMP-2 in LM8 cells to suppress tumor cell growth. Daphnetin (1–30 μM, 72 h) reduced the expression of RhoA and Cdc42, decreased the number of stress fibers and filopodia in LM8 mouse OS cells, and blocked OS cell invasion and migration (246). Therefore, coumarin compounds such as coumarin galbanic acid, esculetin, and 4-methylumbelliferone may inhibit the adhesion, invasion, and metastasis of OS cells by suppressing MMP expression and affecting tumor cell matrix synthesis.

#### 3.7.3 Molecular mechanisms of coumarins in inducing osteosarcoma cell apoptosis

Apoptosis, a major component of programmed cell death (PCD), plays a crucial role in OS development. Dysregulation or reduction of apoptotic function can disrupt the balance between OS cell proliferation and death, increasing the risk of tumorigenesis and metastasis (247). Extrinsic apoptosis, initiated by TNF, induces the aggregation and hydrolysis of Fas-associated death domain-containing protein (FADD) and caspase-8/10, and activates effector caspases-3/7 to target downstream cellular substrates (248). Osthole (0.5–12.5 μM, 24 h) was shown to downregulate BCL-2 and promote the expression of caspase-3 and BAX, thereby inhibiting MG-63 cell proliferation and inducing apoptosis (249). Multiple studies have found that coumarin compounds such as coumarin galbanic acid (25–100 μM, 24–48 h), Urolithin A (75 μM, 24–48 h), and 7-Geranyloxycoumarin (25–100 μM, 24–48 h) significantly increase apoptosis to promote OS cell death and inhibit OS cell proliferation, migration, and adhesion (241, 242, 244). Specifically, apoptotic factors such as caspase-3 and Bax inhibited OS cell proliferation and induced OS cell death following coumarin treatment, thereby impeding tumor cell migration.

The intrinsic apoptotic pathway often results from disruption of the mitochondrial membrane, leading to the release of caspase-9, caspase-activated factor 1 (Apaf-1), and cytochrome c from the mitochondrial intermembrane space into the cytoplasm, which induces activated caspase-9 cleavage and promotes caspase-3/6/7 transcription, along with regulation of Bax expression in the Bcl-2 family (250). Psoralen inhibited the activity of MG-63 cells (8 μg/mL, 48 h) and U2OS cells (10–50 μg/mL, 48 h) and induced cell cycle arrest and apoptosis in a dose-dependent manner. The mechanism was associated with 4-PBA-induced elevation of ATF-6 and CHOP protein levels in the endoplasmic reticulum (ER) and a decrease in Bcl-2 protein levels (251). Lu et al. (252) found that Methoxsalen (500–1,000 μM, 24 h) increased phospholipase C (PLC) levels in MG63 human osteosarcoma cells to promote Ca^2+^ release, and induced Ca^2+^ activity by increasing ER stress, thereby promoting OS cell death. Daphnoretin (1–4 μM, 48 h) disrupted the mitochondrial outer membrane by inhibiting Bcl-2 expression and increasing Bax expression, causing cytochrome c release and activating the caspase-9 and caspase-3 cascade to induce OS cell death (253). Therefore, coumarin-induced apoptosis may have a destructive effect on OS cell proliferation and metastasis, and mitochondrial ER stress may induce cytochrome c release, promoting the accumulation of apoptotic proteins within cells to induce OS cell death.

#### 3.7.4 Regulation of the AKT Signaling pathway to suppress osteosarcoma cell

**Figure 7:**
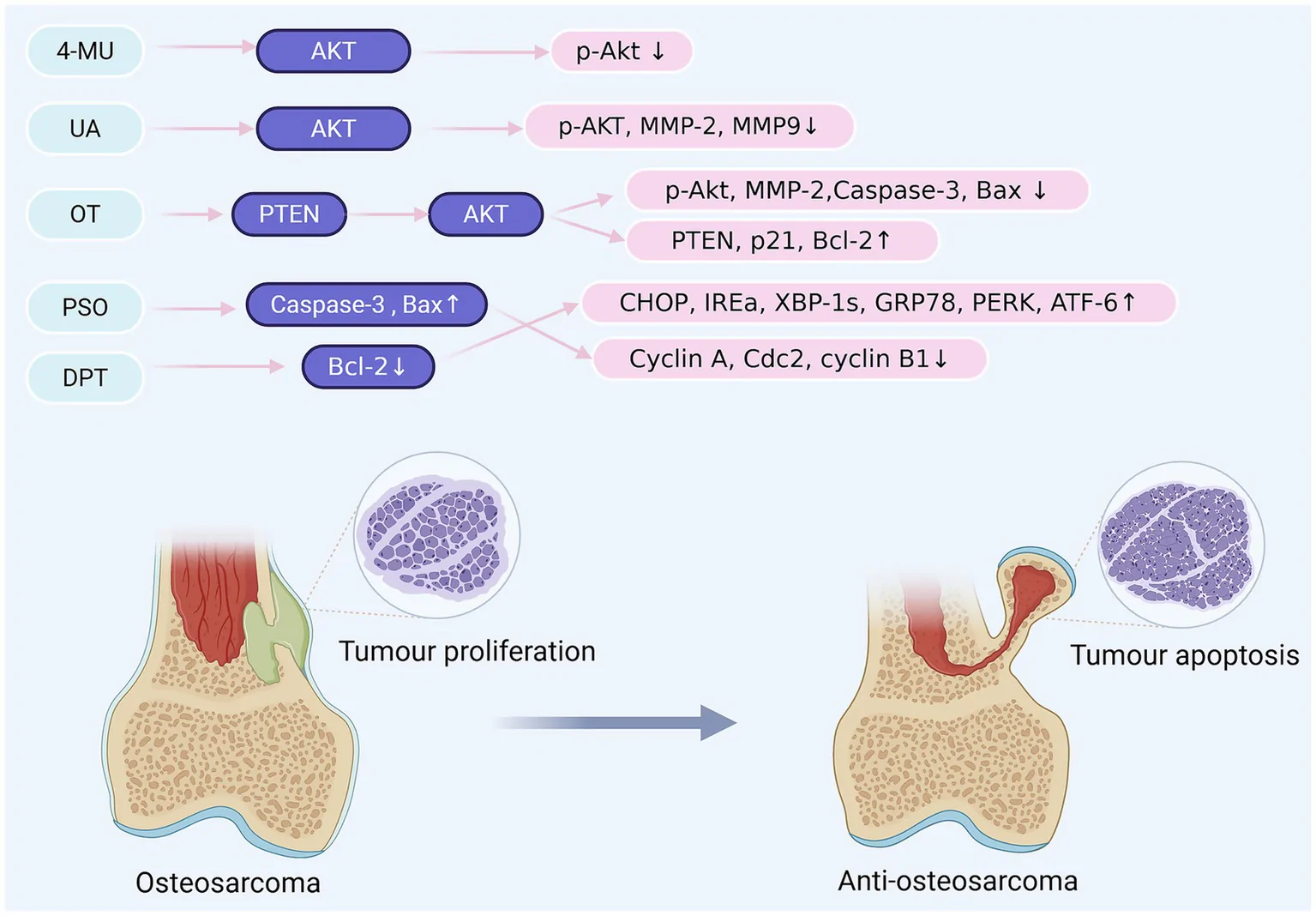
Mechanisms of coumarins in inhibiting osteosarcoma cell proliferation and metastasis via related signaling pathways. 4-MU, 4-methylumbelliferone; UA, urolithin A; OT, osthole; PSO, psoralen; DPT, daphnoretin. ↑, promotion; ↓, inhibition.

AKT, a serine/threonine kinase regulated by the PKB gene, is activated through a cascade of PI3K factors and contributes to OS tumorigenesis (254). AKT3 is the most critical cytokine in the PI3K/AKT pathway, with phosphorylation at threonine 308 and serine 473 catalyzing PDK-1 and mTORC2 activity to induce AKT generation. AKT phosphorylates downstream substrates, promoting OS cell proliferation, migration, invasion, and affecting normal cellular activities (255). 4-Methylumbelliferone (1 mM, 72 h) reduced HA synthesis and altered cell signaling pathways by decreasing Akt phosphorylation expression in MG-63 and HOS cells, thereby inhibiting OS cell proliferation and metastasis (241). Urolithin A (75 μM, 24–48 h) targeted the expression of the key pathways AKT1 and EGFR, regulated MMP activity in MG-63 cells, and effectively inhibited OS cell migration and invasion (242). Specifically, PTEN inactivation induces the accumulation of phosphatidylinositol 3,4,5-trisphosphate (PIP3) and Akt phosphorylation, promoting OS cell proliferation, growth, and survival (256). Osthole (50–150 μM, 24 h) activated PTEN expression to affect Akt phosphorylation, inhibited the cell viability of MG63 and SAOS-2 cells, and significantly induced cell death in a dose- and time-dependent manner, thereby suppressing tumor cell growth (257). In summary, coumarin compounds such as Urolithin A, Osthole, and 4-methylumbelliferone may significantly reduce the proliferative viability of OS cells by modulating MMP and apoptotic pathways. By promoting mitochondrial ER stress and inhibiting the AKT signaling pathway, these compounds not only enhance the expression of apoptosis-related proteins but may also induce cell cycle arrest, demonstrating inhibitory potential against OS cell metastasis and invasion, and highlighting their promise in the regulation of OS therapy (Figure 7). The effects of coumarins on osteosarcoma in *in vivo* and *in vitro* studies are summarized in Supplementary Table S14.

## 4 Discussion

**Figure 8:**
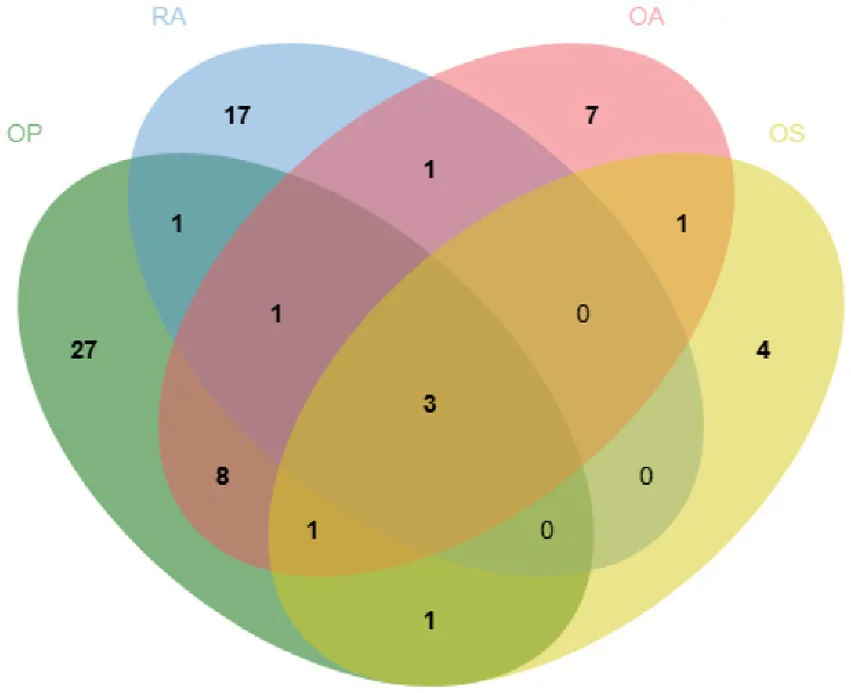
Three common overlapping coumarin compounds, including Urolithin A, Daphnetin, and Osthole, are identified in the treatment of OP, RA, OA, and OS. Each disease corresponds to a single unique coumarin compound.

In the 20th century, the anticoagulant properties of naturally active coumarins sparked growing interest, leading to the development of the vascular anticoagulant drug warfarin. However, subsequent research has gradually revealed the potential of coumarins in combating Musculoskeletal Diseases, with their phytochemical activities primarily focused on potential interventions for OP, RA, OA, and OS. Owing to their pharmacological properties, including anti-inflammatory, antioxidant, antitumor, and bone matrix metabolism regulatory activities, coumarins demonstrate enhanced selectivity for treating musculoskeletal diseases and may potentially serve as modern alternatives for managing these conditions. Coumarins and their derivatives not only mitigate inflammation-induced skeletal tissue damage but also modulate oxidative factors to promote cartilage repair and bone integrity. Among them, three compounds, including Urolithin A, Daphnetin, and Osthole, were commonly involved and studied in the treatment of OP, RA, OA, and OS with coumarins (Figure 8). Through the interplay of pathways such as NF-κB, MAPK, Wnt/*β*-catenin, and PI3K/Akt, they exhibit anti-inflammatory and antioxidant effects that regulate the overall mechanism of bone matrix metabolism. Urolithin A commonly inhibited NLRP3 inflammasome expression in both OP and RA, particularly affecting pyroptosis and inflammation in RAW264.7 and NIH/3 T3 cells through the NF-κB axis. Daphnetin promoted the secretion of the anti-inflammatory factor IL-10 and inhibited the pro-inflammatory expression of IL-6 in RA and OA by blocking the MAPK and NF-κB pathways, highlighting its joint immuno-anti-inflammatory effects in alleviating cartilage degradation. Osthole alleviated the inflammatory response and oxidative stress in OA chondrocytes, as well as the proliferation and migration of OS tumor cells, which was closely associated with the inhibition of PI3K/Akt, NF-κB, and Wnt/*β*-catenin pathway expression. Across numerous animal models and *in vitro* validation studies, these three common types of compounds appear to represent the correlations among the pathways discussed in this review across the various MSDs, particularly demonstrating a positive correlation between coumarin action and signaling pathway research across different fields of MSDs.

In OP studies, similar to Urolithin A, coumarin compounds (Umbelliferone, Bergapten, and Praeruptorin) mainly regulate osteoclast differentiation and maturation and improve bone quality and pathological features by synergistically inhibiting RANK/RANKL-activated NF-κB and MAPK phosphorylation pathways. However, compounds such as Imperatorin and Bergapten primarily promote the activation of the Wnt/*β*-catenin signaling pathway, increasing osteoblast generation particularly under Wnt stimulation, and stimulate BMP-2 and Runx2 expression activity, thereby exerting beneficial effects on BMSCs in OP. In RA studies, coumarins (Umbelliferone and Oxypeucedanin hydrate) block the cross-inflammatory signaling pathways of NF-κB and MAPK, not only alleviating FLS oxidative stress but also inhibiting upstream TLR4 to counteract NF-κB pathway-induced synovitis and joint destruction. In OA studies, coumarins have been demonstrated to primarily interfere with the NF-κB pathway in OA rat cartilage to alleviate the expression of inflammatory factors. They inhibit NF-κB pathway activation and downstream IL-6 secretion, and promote Col-II synthesis, thereby alleviating OA symptoms, by regulating the expression of upstream Sirt1 (Esculin), PI3K/Akt (Urolithin A), and TLR4 (Isofraxidin), respectively. Furthermore, activation of the Sirt1 (Esculin) pathway plays an important role in coumarin-mediated regulation of oxidative factors in OA, which also involves the Keap1/Nrf2 (Psoralen) and PINK1/Parkin (Urolithin A)-related pathways, collectively improving joint oxidative stress and maintaining chondrocyte homeostasis. The inhibitory effect of coumarins upon Wnt/β-catenin activation is mainly reflected in alleviating OA cartilage matrix degradation and degeneration, reducing MMP expression in chondrocytes through this pathway. In OS studies, coumarins inhibit MMP activity and promote the levels of apoptosis-related proteins (such as Caspase and Bax), primarily by blocking AKT and its phosphorylation pathway activation to alleviate tumor cell migration and proliferation.

Alterations in the bone microenvironment, target cell types, and differential cascade signaling pathways appear closely associated with ROS signaling, leading coumarins to exhibit dual roles in modulating mitochondrial oxidative stress: they inhibit ROS levels in OP, RA, and OA to alleviate skeletal damage, yet elevate oxidative factor levels in OS to exert anti-tumor migration effects. ROS signaling is involved in multiple biological processes, including cell death, inflammation, and proliferation. Under elevated oxidative stress during OP, RA, and OA progression, coumarins act as antioxidants to suppress ROS-induced bone destruction and tissue damage. Specifically, coumarins scavenge excessive ROS to maintain normal metabolic pathways in bone and joint tissues, thereby reducing the susceptibility of impaired osteoblasts and chondrocytes to oxidative stress-induced cell death (apoptosis and pyroptosis). In OS, however, coumarins disrupt the antioxidant system to elevate ER stress beyond a lethal threshold, enhancing the immune microenvironment and thereby regulating tumor cell migration and promoting apoptosis. This OS regulatory mechanism—utilizing enhanced NADPH oxidase activity to induce tumor cell death—appears consistent with the ROS-dependent antitumor effects of other natural compounds. It is noteworthy that ROS-mediated signaling exerts dual roles depending on cell type specificity within musculoskeletal tissues. Coumarins activate pro-survival pathways, such as Wnt/*β*-catenin, TGF-β, and p38 MAPK in OP osteoblasts and the Nrf2/ARE pathway in OA chondrocytes, to counteract ROS and oxidative stress. Conversely, in cartilage damage, they mitigate mitochondrial redox activity and reduce chondrocyte death and matrix degradation by blocking NF-κB, PI3K/AKT, and Wnt/β-catenin pathways. Thus, the oxidative regulatory nature of coumarins is not intrinsically pro-oxidant or antioxidant but is context-dependent based on cellular specificity, rendering them versatile modulators for treating different Musculoskeletal Diseases.

Epigenetic mechanisms play a key role in regulating the complex interplay between inflammatory factors and the bone microenvironment. Through processes such as DNA methylation, histone and chromatin modifications, and non-coding RNA-mediated regulation, epigenetics modulates gene expression to influence bone mass, bone remodeling, and cellular homeostasis in synovial tissues (258). Coumarins have been shown to significantly impact neuromuscular function and lipid metabolism by influencing epigenetic changes in F0–F3 germ cells (259). Similarly, Fraxetin treatment in MG-63 cells inhibited TNF-*α*, IL-1β, caspase-8, and caspase-3 expression activity by counteracting anti-Fas IgM-induced apoptotic DNA fragmentation, thereby antagonizing inflammation-mediated apoptosis in osteoblasts to prevent OP (137). Modifications in the VIM gene encoding the columbianadin inhibitor reduced the apoptosis rate in FLS cells; however, columbianadin itself inhibited vimentin overexpression and decreased mitochondrial membrane potential, blocking VAV2/Rac-1-induced synovial hyperplasia in RA (160). Esculin, a coumarin derivative, upregulates silent information regulator 1 (SIRT1) activity to inhibit downstream NF-κB expression, reducing joint levels of COX-2, iNOS, and MMP-13 and suppressing pro-inflammatory factors and joint degeneration in chondrocytes (196). Therefore, coumarin-modulated epigenome-wide association studies have revealed susceptibility loci for Musculoskeletal Diseases, and elucidating biological processes such as deacetylase activity will enhance future understanding of treating these diseases. Moreover, exploring genetic function-based technologies (GFT) for coumarins, such as TACE inhibitors and TGF-β1 product markers (260, 261), may offer positive implications for improving oral bioavailability, bone quality, and anti-inflammatory efficacy.

Coumarins, as a class of chemically diverse compounds, can interfere with the metabolism or interaction of other drugs. Furanocoumarin derivatives such as bergamottin, important components of grapefruit juice, are inhibitors of the CYP3A4 hepatic microsomal enzyme and can reduce the metabolism of several related drugs, including calcium channel blockers (nitrendipine) or statins (simvastatin), thereby increasing adverse effects in humans (262). Particular caution should be exercised because coumarin-containing dried plants may, under certain circumstances, have the capacity to produce dicoumarins, which are contraindicated especially in patients receiving coumarin anticoagulants (e.g., sintrom, aspirin, and clopidogrel) who present with bleeding, as this may also lead to hepatitis, allergic reactions, and cholesterol embolism (263). Furthermore, warfarin, an anticoagulant drug that uses coumarin as an important raw material, is used to treat thromboembolism and pulmonary embolism associated with atrial fibrillation. However, there is an 84% potential increased risk of bleeding due to warfarin effects. Long-term administration of anticoagulants for more than two years in elderly patients with atrial fibrillation can cause bleeding (gastrointestinal and respiratory) and is closely associated with a fourfold increased risk of newly diagnosed cancer (264). The use of P2Y12 inhibitors or combination therapy with warfarin increases the probability of spontaneous fundus hemorrhage in elderly patients within 90 days (265). Therefore, ethyl biscoumacetate, a semi-synthetic compound, is used as an alternative anticoagulant to warfarin due to its fewer side effects. Additionally, patients receiving warfarin therapy for more than one year are associated with a higher risk of OP fractures (266). This highlights the importance of careful selection of coumarins and bleeding prevention in the context of anti-MSD progression, especially in patients with concurrent gastrointestinal bleeding. Meanwhile, coumarin compounds that may mask or interfere with diagnostic or clinical follow-up symptoms warrant attention. For example, methoxypsoralen derivatives are potent photosensitizers that can become mutagenic, phototoxic, and carcinogenic upon activation by near-ultraviolet radiation (267). 6,7-Furanocoumarins can induce photodermatitis, burns, and the risk of melanoma upon prolonged sun exposure due to their photosensitizing properties (268). Excessive application of furanocoumarins present in plants can induce plant photodermatitis, which is activated upon exposure to ultraviolet radiation, particularly UVA rays. Furanocoumarins such as psoralen, angelicin, and imperatorin (an 8-methoxypsoralen derivative) are also commonly used in the treatment of skin diseases such as vitiligo, psoriasis, eczema, or cutaneous T-cell lymphoma due to their photosensitizing properties (269). However, plant photodermatitis may also induce contact dermatitis or allergic contact dermatitis, which needs to be distinguished from MSDs. Importantly, the diagnosis of plant photodermatitis includes the identification of its unique clinical features (e.g., photosensitivity disorders, blisters, and hyperpigmentation), which should be differentiated from other allergic dermatological symptoms induced by plants, chemicals, or environmental factors. Although certain MSDs (such as RA and OA) share similar symptoms with skin diseases, including burning sensation, edema, and erythema, healthcare providers may be able to accurately distinguish between skin diseases and MSDs by carefully evaluating patient history, conducting skin patch tests, considering plant exposure, and performing subsequent imaging examinations, thereby preventing misdiagnosis and inappropriate treatment and ensuring optimal patient outcomes. Currently, most coumarin-related clinical studies are still in the early stages with relatively small sample sizes, and further research on additional clinical applications and follow-up is needed to confirm the reliability and broad applicability of the results.

## 5 Limitations and future perspectives

Although basic research has provided some mechanistic insights into the regulation of musculoskeletal diseases by coumarins, several limitations and challenges warrant attention. Regarding research methodology: First, the search was limited to PubMed, Web of Science, and Embase, which may have omitted relevant studies from other databases (such as China National Knowledge Infrastructure, CNKI). Second, only English-language articles were included, which may have introduced language bias. Third, heterogeneity in experimental models and outcome measures precluded a meta-analysis. Fourth, there were some variations in the quality of the included studies. Although we performed formal risk of bias assessments and stratified the level of evidence using tools such as the OHAT risk-of-bias tool, the strength of the conclusions is limited by small sample sizes, short durations, and incomplete reporting. The consistent findings from animal and *in vitro* studies support the biological plausibility of coumarins as bone-protective agents; however, future studies should incorporate additional risk of bias assessment tools (e.g., SYRCLE RoB) for more comprehensive literature quality evaluation. Regarding research mechanisms: First, coumarins are susceptible to various environmental factors (e.g., soil nutrients, water content, temperature, and light), and their physicochemical properties are often unstable, with low oral bioavailability, which affects the therapeutic efficacy in skeletal diseases. The use of modern biotechnologies (e.g., nanotechnology) with liposomal delivery systems may improve drug permeability, enhance precise targeting, increase selective drug absorption, and reduce component degradation. Second, although the mechanisms by which coumarins regulate musculoskeletal diseases may have been validated in animal experiments, robust verification of their efficacy and safety in humans through clinical trials is indispensable. Future studies should select effective doses of coumarins based on safe dose ranges to mitigate disease progression in patients, and epigenetic-based drug therapy may improve the efficacy and safety of coumarin treatment. Finally, the interventional effects of novel synthetic coumarins in combination with conventional drugs require further exploration. Most synthetic coumarins are designed to modify the benzopyrone ring to enhance anti-bone resorption and anti-bone degeneration capacity. Whether their effects can enhance efficacy or achieve synergistic therapeutic effects with non-steroidal anti-inflammatory drugs, anti-resorption inhibitors, or immunotherapies, while reducing side effects, warrants active investigation.

The gut microbiota plays a crucial regulatory role in substance metabolism, growth and development, as well as bone metabolism and homeostasis (270). Disruption of the gut microbiota (dysbiosis) may accelerate bone destruction and loss by increasing intestinal permeability, promoting systemic inflammation, and impairing nutrient absorption (71). Studies in germ-free mice have revealed a significant reduction in osteoclast numbers and decreased levels of pro-inflammatory mediators (IL-6, PGE-*α*, TNF-α, and RANKL), which were restored upon microbiota reconstitution. For example, oral administration of the coumarin extract BX (once daily, 15–135 mg/kg for 12 weeks) increased the F/B ratio, enriched beneficial intestinal bacterial genera such as Faecalibaculum and Phascolarctobacterium in OVX mice, reduced the abundance of Alloprevotella, and increased serum levels of BALP and PINP, thereby maintaining the balance between bone resorption and new bone formation in OP (66). Nodakenin treatment (once daily, 20 mg/kg for 6 weeks) improved intestinal villus height, reduced crypt depth and the expression of intestinal inflammatory factors in OVX mice, increased the relative abundance of Muribaculaceae, Parasutterella, Faecalibaculum, Allobaculum, and Coriobacteriaceae UCG-002, and decreased the relative abundance of Roseburia, Blautia, and Lachnoclostridium (69). In RA, oral administration of Columbianadin (once daily, 20 mg/kg for 3 weeks) promoted the relative abundance of beneficial bacteria such as Lactobacillaceae, Rikenellaceae, and Lactobacillus in mice, reduced the relative abundance of harmful bacteria including Bacteroidaceae, Ruminococcaceae, and Enterobacteriaceae, restored normal microbiota balance, and inhibited RA joint and synovial inflammation (271). In OA, oral administration of Isopsoralen (once daily, 10 mg/kg for 5 weeks) restored beneficial gut microbiota including Blautia, Allobaculum, Lachnospiraceae_NK4A136, and Dubosiella in DMM mice, and inhibited harmful bacteria such as Erysipelatoclostridium, Parabacteroides, Anaerovorax, Helicobacter, Faecalibaculum, Desulfovibrio, and Bacteroides, thereby improving gut microbiota balance to alleviate OA inflammation and bone microstructural destruction (272). Therefore, coumarins may reduce pathological bone loss and promote cartilage and bone growth and repair through the gut-immune-bone axis, gradually highlighting the potential of coumarins in gut-mediated immunomodulation for maintaining bone health. However, research on the intestinal bioavailability and *in vivo* metabolic fate of coumarins remains insufficient, and additional regulatory mechanisms of gut microbiota and their metabolite changes in OS-related tumor migration and proliferation warrant further exploration.

## 6 Conclusion

Coumarins possess extensive biological mechanistic capabilities and potential for clinical translation, representing an indispensable class of novel phytochemical compounds for targeting and intervening in musculoskeletal diseases. The translation of these compounds still requires validation in rigorous clinical settings, and their current applicability is limited by challenges such as data standardization, model interpretability, and *in vivo* safety assessment.
