# Supplementary material for: Coumarins in musculoskeletal health: a systematic review
Source: Front Nutr. 2026 Sep 9;13:1737767. doi: 10.3389/fnut.2026.1737767 (PMC13597780; doi:10.3389/fnut.2026.1737767)
Supplement: Supplementary file 1 [file Supplementary_file_1.zip › Supplementary Tables S11-S14.DOCX]

**Supplementary Table S11. Mechanisms of coumarins in vivo and in vitro models of osteoporosis.**

| Coumarin compounds | Source | Availability part | Intervention model | | Intervention dosage | Signalling pathway | Intervention target | References |
| --- | --- | --- | --- | --- | --- | --- | --- | --- |
|  |  |  | In vivo/  In vitro | Animal  /Cell |  |  |  |  |
| Urolithin A (UA) | Pomegranates and Nuts | Fruit | In vivo | C57BL/6 mice | 10 and 20 mg/kg/day UA for 8 weeks | NF-κB | TNF-α, IL-6, IL-1β, Acp5, CTSK, OSCAR, NFATc1, MMP9, NLRP3, Caspase-1, GSDMD, IL-18 and RANKL/OPG↓; IL-10↑ | (63) |
|  |  |  | In vitro | RAW 264.7 cells | 5 and 10 μM UA |  |  |  |
| Pteryxin (PTX) | Peucedanum | Root | In vivo | C57BL/6 mice | 5 and 10 mg/kg/day PTX for 7 weeks | ERK, p38 MAPK, JNK, NF-κB | p-ERK 1/2, p-p38 MAPK, p-JNK 1/2, p-p65, RANKL, Ca^2+^, c-FOS, NFATc1, CTSK↓ | (64) |
|  |  |  | In vitro | BMM and MC3T3-E1 cells | 10 and 20 μM PTX |  |  |  |
| BX | Psoraleae Fructus | Fruit | In vivo | C57BL/6 mice | 15, 45, and 135 mg/kg/day BX for 12 weeks | N/A | BALP↓; PINP↑ | (65) |
| Psoralen (PSO) | Psoralea corylifolia | Seed | In vivo | Kunming (KM) mice | 10, 20, and 40 mg/kg/day PSO for 4 weeks | N/A | HSD17B2↓; OCN, ALP↑ | (66) |
|  |  |  | In vitro | HepG2 and 293T cells | 0.01, 0.1, 1, 5, 10 and 100 μM PSO |  |  |  |
| Isoimperatorin (ISO) | Angelica dahurica, Adenophora japonica | Root | In vivo | C57BL/6 mice; | 5 and 10 mg/kg/day ISO for 8 weeks | N/A | OPN, BGLAP, RUNX2, ALP, BGLAP↑ | (67) |
|  |  |  | In vitro | BMM and MC3T3-E1 cells | 0, 2.5, 5 and 10μM ISO |  |  |  |
| Nodakenin (NDK) | Angelica sinensis | Root | In vivo | C57BL/6 mice | 20 mg/kg/day NDK for 6 weeks | N/A | TRAP, RANKL, IL-1β, TNFα↓; B-ALP, BGP, VDR, ALP, Col-1, OCN↑ | (68) |
| Coumarin Derivative KY-054 | Aesculetin, Osthole, and  Scopolin | Synthesized | In vivo | F344/NSlc rats | 3 and 10 mg/ml/kg/day KY-054 for 8 weeks | N/A | CDK8↓; ALP↑ | (69, 70) |
|  |  |  | In vivo | ST2 cells | 0, 2.5, 5 and 10μM KY-054 |  |  |  |
| Dalbergin（DBN） | Dalbergia sissoo | Bark | In vivo | BALB/c mice | 1-10 mg/kg/d for 8 weeks | N/A | TRAP, RANK↓; OPG/RANK, OCN, OSX, RUNX2、BMP2, COL1↑ | (71) |
|  |  |  | In vitro | BMSCs | 100 pM, 10 mM and 1 μM |  |  |  |
| Methoxsalen (MTS) | Ammi majus, celery, parsley, carrot, orange | Fruit | In vivo | C3H/HeN mice | 0.02% MTS | NF-κB | IL-6↓; Runx-2, OSX, OCN, OCN↑ | (72) |
| Bergapten（BP） | Bergamot essential | Flavedo | In vivo | C57BL/6 mice | 20 mg/kg/d for 12 weeks | Wnt/β-catenin | ALP, OSX , Cola1, GSK3β, β-catenin, RUNX-2, OCN↑ | (73) |
|  |  |  | In vitro | BMSCs | 0.1, 1 and 10μM |  |  |  |
| Wedelolactone (WED) | Ecliptae | Leaves | In vivo | C57BL/6 mice | 20 mg/kg/d for 12 weeks | Wnt/CK2α/GSK3β/β-catenin | p-ERK1/2↓; p-GSK3β, ALP, OSX, OCN, Runx2↑ | (74) |
|  |  |  | In vitro | BMSCs | 1μg/mL and 30 µM |  |  |  |
| Bergamottin (BM) | Grapefruits and  pomelos | Fruit | In vivo | C57BL/6 mice | 15 and 30 mg/kg/d for 8 weeks | Wnt/β-catenin | GSK3β↓;LRP6, Col-1, TCF4, TCF7, ALP, OCN, Runx2↑ | (75) |
|  |  |  | In vitro | MC3T3-E1 cells | 5, 10, and 20 μM |  |  |  |
| Coumarin−Dihydropyridine Hybrids | Hybridization of coumarin and dihydropyridine subunits | Synthesized | In vivo | C57BL/6 mice | 1, 5, 10 mg/kg/d for 8 weeks | N/A | OPG/RANKL↓; Runx-2, ALP, Col-I,  BMP-2, and OCN↑ | (76) |
|  |  |  | In vitro | Osteoblasts cells | 10nM-100μM |  |  |  |
| Isopsoralen (IPL) | Psoralea corylifolia | Fruit | In vivo | Balb/c mice | 5, 10 and 20mg/kg/day ISO for 4 weeks | AhR/ERα, cGMP/PKG | AhR, CYP1A1, PDE5↓; ERα, cGMP, PKGI, PKGII, Runx2, ALP↑ | (78, 87) |
|  |  |  | In vitro | MC3T3-E1 cells | 2-50μmol/L |  |  |  |
| Osthole | Fructus Cnidii | Fruit | In vivo | C57BL/6 mice | 5 mg/kg/d for 8 weeks | N/A | NFATc1, c‑Fos, RANKL, MMP-9↓; OCN, OPG↑ | (79) |
| Bergapten and Methoxsale | celeriac, celery, lemon, parsley, parsnip | Fruit, vegetable | In vivo | C57BL/6 mice | 0.02% at HFD diet for 4 weeks | N/A | GSK3β, β-catenin, RUNX2, NFATc1 , TRAP↓; OPG, OCN↑ | (80) |
| Bergapten (BP) | bergamot, grapefruit | Essential oil | In vivo | C57BL/6 mice; | 10 and 20 mg/kg/d for 4 weeks | ERK, p38 MAPK, JNK, NF-κB | p-ERK 1/2, p-p38 MAPK, p-JNK 1/2, p-p65, NFATc1, c-fos, TRAP, CK, V-ATPase a3, VATPase d2↓ | (81) |
|  |  |  | In vitro | BMMs and MC3T3-E1 cells | 1.2, 2.5, and 5μM |  |  |  |
| Psoralen | Psoralea corylifolia | Fruit | In vitro | BMSCs | 20 µg/ml | N/A | Runx2, OSX, ALP↑ | (86) |
| Aesculetin (AES) | Cortex Fraxini | Bark | In vitro | MC3T3-E1 cells | 1, 5, and 10 μM | N/A | Runx2, Col-1, ALP, PHOSPHO1, Tenascin C↑ | (88) |
| Wedelolactone (WED) | Ecliptae | Leaves | In vivo | C57BL/6 mice | 10 mg/kg/d for 4 weeks | Wnt/CK2α/GSK3β/β-catenin | p-ERK1/2↓; p-GSK3β, CK2α, ALP, Runx2↑ | (89) |
|  |  |  | In vitro | BMSCs | 1μg/mL and 30 µM |  |  |  |
| Imperatorin (IMP) | Angelica archangelica | Root | In vivo | SD rats | 50 mL/kg/d for 12 weeks | AKT/GSK3 β/β-catenin | p-AKT, p-GSK3β, β-catenin, RUNX-2, OCN, COL1A1↑ | (90) |
|  |  |  | In vitro | BMSCs | 25, 50 and 75μM |  |  |  |
| Dalbergin（DBN） | Dalbergia sissoo | Bark | In vivo | BALB/c mice | 0.1-5 mg/kg/d for 6 weeks | N/A | TRAP, RANK↓; OPG, OCN, OSX, RUNX2, ALP, BMP2, COL1↑ | (92) |
|  |  |  | In vitro | Primary osteoblast cells | 100pM, 10mM and 1μM |  |  |  |
| Isocoumarin A | P. amplexicaule | Roots | In vitro | MC3T3-E1 cells | 2.5, 5 and 10μM | PI3K/Akt/Erk | p-Akt, p-Erk, ALP, OCN, COL-1, BMP-2, RUNX2, ALP↑ | (93) |
| Osthole | Fructus Cnidii | Fruit | In vivo | SD rats | 100 mg/kg/d for 8 weeks | BMP-2/p38 MAPK, Erk1/2, Wnt/β-catenin, | p-ERK1/2, p-p38 MAPK, BMP-2, SMAD1/5/8, ALP, Wnt1, Wnt3a ,  Wnt4, β-catenin, OCN, COL1, OSX↑ | (95, 96, 97) |
|  |  |  | In vitro | MG-63 cells | 5, 10, 20, 50 and 100μM |  |  |  |
| 5′-Hydroxy Auraptene (5′-HA) | Lotus lalambensis Schweinf | Roots | In vitro | BMSCs | 1–100μM | N/A | BMP-2, ALP, OCN, Dlx5, Msx2, p-SMAD1/5/8,  Smad4 ↑ | (98) |
| Psoralen | Psoralea corylifolia | Fruit | In vitro | Primary calvarial osteoblasts | 1, 10 and 100μM | N/A | Bmp2, Bmp4, p-SMAD1/5/8, Col1a1, Alp, Oc, Bsp↑ | (99) |
| Imperatorin(IP) and Bergapten (BP) | Angelica archangelica, bergamot, grapefruit | Root, Essential oil | In vivo | SD rats | 30 μM/d IP and 10 μl/d BP for a week | p38 MAPK, ERK1/2 | BMP-2, p-ERK1/2, p-p38 MAPK, Collagen, ALP↑ | (100) |
|  |  |  | In vitro | Primary osteoblastic cells | 1, 3 and 10μM |  |  |  |
| Psoralidin (PSD) | Psoralea corylifolia | Fruit | In vitro | Primary osteoblastic cells | 10^−8^, 10^−6^, 10^−4^ mol/l | N/A | IGF-1, β-catenin, Runx-2, OSX, RANKL, OPG, Nrf2, COX-2↓; BMP-2, OCN, Col-I, ALP↑ | (104) |
| Isoimperatorin (ISO) | Angelica dahurica, Adenophora japonica | Root | In vitro | BMMs | 2–10 μM | ERK、p38 MAPK, JNK, NF-κB | CP5, c-Fos, CTSK, ATP6V0D2, [NFATc1](https://www.sciencedirect.com/topics/biochemistry-genetics-and-molecular-biology/transcription-factor-nfat), Ca^2+^, ACP5, C-Fos, CTSK, ATP6V0D2, [NFATc1](https://www.sciencedirect.com/topics/immunology-and-microbiology/transcription-factor-nfat), p-ERK 1/2, p-p38 MAPK, p-JNK 1/2, p-p65↓ | (108) |
| Osthole (OT) | Fructus Cnidii | Fruit | In vivo | C57BL/6 mice | 10 mg/kg/d for 12 weeks | N/A | NFATc1, c-Fos, MMP-9, Ctsk, TRAP, Car2↓ | (109) |
|  |  |  | In vitro | BMMs | 0.5, 1, 2, 3, 4 and 5μM |  |  |  |
| Psoralidin | Psoralea corylifolia | Fruit | In vivo | C57BL/6 mice | 5 and 50 mg/kg/d for a week | ERK, p38 MAPK, JNK, NF-κB | p-ERK 1/2, p-p38 MAPK, p-JNK 1/2, p-p65, TNF-α, IL-1β, IL-6, RANKL, NFATc1, c-Fos↓; OPG↑ | (110) |
|  |  |  | In vitro | BMMs | 1, 10, and 30μM |  |  |  |
| Praeruptorin A | Peucedanum praeruptorum Dunn | Root | In vitro | BMMs | 10μM | p38 MAPK/Akt-c-Fos-NFATc1 | c-Fos, TRAP, NFATc1, Ca^2+^↓ | (111) |
| Xanthotoxin (XAT) | Ammi majus | seeds | In vitro | BMMs | 0.1, 1, and 5μM | CaMKK/PYK2 | CaMKK, PYK2, c-Fos, TRAP, NFATc1, RANKL, Ca^2+^↓ | (112) |
| Aesculin (AES) | Aesculus hipcasanum (horse chestnut) | Fruit | In vivo | SD rats | 25 and 100 mg/kg/d for 4 weeks | ERK, p38 MAPK, JNK, NF-κB | p-ERK 1/2, p-p38 MAPK, p-JNK 1/2, p-p65, Trap, Atp6v0d2, Cathepsin K,RANK, MMP-9↓ | (115) |
|  |  |  | In vitro | RAW264.7 cells | 5, 10, and 20μM |  |  |  |
| Bergapten (BP) | bergamot, grapefruit | Essential oil | In vivo | C57BL/6 mice; cells | 10 and 20 mg/kg/d for 4 weeks | ERK, p38 MAPK, JNK, NF-κB | p-ERK 1/2, p-p38 MAPK, p-JNK 1/2, p-p65, NFATc1, c-fos, TRAP, CK, V-ATPase a3、VATPase d2↓ | (116) |
|  |  |  | In vitro | BMMs and MC3T3-E1 | 1.2, 2.5, and 5μM |  |  |  |
| Osthole | Fructus Cnidii | Fruit | In vitro | RAW264.7cells | 10^−6,^ 10^−5^ mol/l | NF-κB | NFATc1, TRAP, β3-Integrin, c-Src, MMP-9, CTSK↓ | (117) |
| Osthole-loaded N-octyl-O-sulfonyl chitosan micelles (NSC-OST) | Fructus Cnidii | Fruit | In vivo | SD rats | 100 mg/kg/d for 12 weeks | NF-κB | NFATc1, c-Fos, CTSK, TRAP, MMP-9, p-IκB, p-p65↓ | (118) |
|  |  |  | In vitro | RAW264.7cells and BMMs | 25 ng/mL |  |  |  |
| Citrus Nobiletin (CN) | Citrus fruits | Fruit | In vivo | DAB/1J mice | 2 and 20 mg/kg/d for 12 weeks | ERK, p38 MAPK, JNK, NF-κB | p-ERK 1/2, p-p38 MAPK, p-JNK 1/2, p-p65↓ | (119) |
|  |  |  | In vitro | RAW264.7 cells | 4, 20, and 50 µM |  |  |  |
| Isofraxidin (IFD) | Fraxinus, Eleutherococcus | Bark, Root | In vitro | BMMs | 6.25 and 12.5 μM | AKT, NF-κB | p-AKT, p-p65, p-IκBα, MMP-9, c-Src, cathepsin K, NFATc1↓ | (120) |
| Praeruptorin B | Peucedanum praeruptorum Dunn | Root | In vivo | C57BL/6 mice | 10 mg/kg/d for 6 weeks | NF-κB | p-p65, IKKβ↓; p-GSTP1↑ | (121) |
|  |  |  | In vitro | BMMs and MC3T3-E1 cells | 5 and 10 μM |  |  |  |
| Urolithin B | pomegranate, walnut, berries | Fruit | In vivo | C57BL/6 mice | 10 and 20 mg/kg/d for 8 weeks | ERK, p38 MAPK, JNK, NF-κB | p-ERK 1/2, p-p38 MAPK, p-JNK 1/2, p-p65, MMP-9, CTSK, TRAP, ATPase, c-fos, NFATc1↓ | (122) |
|  |  |  | In vitro | RAW264.7 cells | 1, 5, and 25μM |  |  |  |
| Urolithin A | Pomegranates, nuts | Fruit | In vivo | C57BL/6 mice | 10 and 20 mg/kg/d for 8 weeks | ERK, p38 MAPK, JNK, NF-κB | p-ERK 1/2, p-p38 MAPK, p-JNK 1/2, p-p65, MMP-9, CTSK, TRAP, ATPase, c-fos, NFATc1↓ | (124) |
|  |  |  | In vitro | RAW264.7 cells | 1, 5, and 25μM |  |  |  |
| 5'-hydroxy auraptene (5'-HA) | Lotus lalambensis Schweinf | Leaves | In vitro | BMMs | 1, 10, and 50μM | ERK, p38 MAPK, JNK, NF-κB | p-ERK 1/2, p-p38 MAPK, p-JNK 1/2, p-p65, Ctsk, Trap, MMP-9, NFATc1↓ | (125) |
| Umbelliferone (UMB) | carrot, angelica, coriander | Fruit, Leaves | In vivo | ICR mice | 60 and 120 mg/kg/d for 24 weeks | Akt-c-Fos-NFATc1 | p-AKT, p-ERK 1/2, p-p38 MAPK, p-JNK 1/2, p-p65, c-Fos, NFATc1, CTX-I , OCN↓ | (127) |
|  |  |  | In vitro | BMMs | 5, 10, and 50μM |  |  |  |
| Daphnetin | Daphne genus | Root,  Flowers,  Fruit, Leaves | In vitro | BMMs | 20 and 40 μM | NF-κB, Akt/GSK‐3β | p‐p65, p‐IκBα, GSK‐3β, TRAF6、c-Fos、NFATc1, cathepsin k↓ | (128) |
| Osthole and Notopterol | Angelica  Pubescens, Notopterygium franchetii | Root | In vitro | AB strain zebrafish | 1, 2 and 4 μM | N/A | NO, ROS↓ | (131) |
| Urolithin B | pomegranate, walnut, berries | Fruit | In vitro | RAW264.7 cells | 10, 50 and 100 μM | ERK, p38 MAPK, JNK, NF-κB | p-AKT, p-ERK, p-p38 MAPK, p-JNK 1/2, p-p65, c-Fos, NFATc1↓; Nrf2, HO-1, CAT, GCLC↑ | (134) |
| Fraxin | Acer tegmentosum, Fraxinus ornus | Bark | In vivo | C57BL/6 mice | 6 mg/kg/d for 7 weeks | p38 MAPK, JNK, NF-κB | p-ERK, p-p38 MAPK, p-IκB-α, NFATc1, MMP-9, Ca^2+^,  cathepsin K, c-Fos, Acp5, Atp6v0d2↓; HO-1, CSR, Catalse↑ | (135) |
|  |  |  | In vitro | BMMs | 5 and 10μM |  |  |  |
| Fraxetin | Cortex Fraxini | Bark | In vitro | MC3T3-E1 cells | 1-20 μM | N/A | TNF-α, IL-1β, caspase-8, caspase-3↓ | (137) |
| Orcinol (ORC) | Curculigo orchioides Gaertn | Root | In vitro | U937 cells | 2.5, 5 and 10μM | NF-κB | p-IκB-α, p-p65↓ | (138) |
| Osthole | Fructus Cnidii | Fruit | In vivo | SD rats | 400 mg/kg/d for 4 weeks | N/A | Ca^2+^, Mg^2+^, AKP↓; NO, NOS, OCN↑ | (139) |

**Supplementary Table S12. Mechanisms of coumarins in vivo and in vitro models of rheumatoid arthritis.**

| Coumarin compounds | Source | Availability part | Intervention model | | Intervention dosage | Signalling pathway | Intervention target | References |
| --- | --- | --- | --- | --- | --- | --- | --- | --- |
|  |  |  | In vivo/  In vitro | Animal  /Cell |  |  |  |  |
| Isopsorale (IPRN) | Psoralea corylifolia | Fruit | In vivo | DBA/1J mice | 5 and 20 mg/kg/day UA for 8 weeks | N/A | IL-6, IL-8, MMP1, MMP3, CXCL9, and CXCL10, COMP↓; IPRN, IL-10↑ | (147) |
|  |  |  | In vitro | RA FLS cells | 10 μM and 20 μM ISO |  |  |  |
| Urtica dentata Hand (UDH) | Laportea bulbifera | Root | In vivo | Balb/c mice | 20, 40 and 60 mg/kg/day PTX for 4 weeks | N/A | IFN, IL-2, T-bet↓; IL-10, CD4, CD25, Foxp3↑ | (148) |
| 8-methoxy chromen-2-one (MCO) | Ruta graveolens | Leaves | In vivo | Wistar rats | 2, 5, and 20 mg/kg/day BX for 3 weeks | N/A | TNF-α, IL-1β, IL-6, NO, iNOS↓ | (149) |
|  |  |  | In vitro | J774 cells | 5, 10 and 20 μg/ml MCO |  |  |  |
| Scopolin | Erycibe obtusifolia Banth | Stems | In vivo | Wistar rats | 50 and 100 mg/kg/day for 3 weeks | N/A | IL-6, VEGF, FGF-2↓ | (151) |
| Daphnetin (DAP) | Daphne odora Var. marginata | Root | In vivo | Wistar rats | 1 and 4 mg/kg/day for 3 weeks | NF-κB | IL-17, IL-6, IL-4, INF-γ,  TGF-β1, IL-22, IL-23, RORγt, NF-κB, Foxp3, CD77↓; Foxp3, IL-10↑ | (152) |
| 3-phenylcoumarin derivative 6,7-dihydroxy-3-[3',4'-methylenedioxyphenyl]-coumarin | Chemical synthesis | Synthesis | In vivo | Wistar rats | 1.5 mg/kg/day for 6h | N/A | TNF-α, IL-1β, IL-6↓ | (153) |
| Auraptene (AUR) and Umbelliprenin (UMB) | Citrus and Ferula fruit | Peel, Fruit | In vivo | Wistar rats | 16, 32, and 64 μm/kg/day AUR and UMB for 2 weeks | N/A | IL-17, PGE-2, NO, IFN-γ, IL-2, TNF-α↓; IL-4, IL-10↑ | (154) |
| Glytabastan B (GB) | Glycine tabacina | Root | In vitro | SW982 synovial cells | 3 and 6μM | ERK, p38 MAPK, JNK, PI3K/AKT | ERK 1/2, p-p38 MAPK, p-JNK 1/2, p-p65, p-PI3K, p-AKT, COX-2, MMP-9, TNF-α, IL-6, IL-8, MMP-1↓ | (155) |
| Columbianadin (CBN) | Angelicae pubescentis radix | Root | In vivo and  in vitro | DBA/1 J mice | 10 and 40 mg/kg/day CBN for 6 weeks | VAV2/Rac-1 | p62, Beclin-1, BAX, Bcl-2, p53, MMP2, MMP9, p-VAV2, Rac-1↓ | (160) |
|  |  |  | In vitro | MH7A cells | 5, 25, and 50 μm |  |  |  |
| Umbelliferone (UMB) | carrot, angelica, coriander | Fruit, Leaves | In vivo | SD rats | 25 and 50 mg/kg/day for 4 weeks | Wnt/β-catenin | Wnt1, p-GSK-3β, β-catenin, IL-1β, IL-6, IL-8, TNF-α, MMP-2, MMP-9↓ | (161) |
|  |  |  | In vitro | MH7A cells | 0-160 μM |  |  |  |
| Daphnetin | Daphne odora Var. marginata | Root | In vitro | RA FLS cells | 40μg/mL | N/A | FasL, Cyt-c, Bax, Caspase-3, Caspase-8, Caspase-9↑; Bcl2↓ | (162, 163) |
| Imperatorin | Angelica archangelica | Root | In vivo | Wistar rats | 5 mg/kg/day for 7 weeks | N/A | MMP-1, MMP-3, Bcl-2↓; Bax, cleaved caspase-3, cleaved caspase-9, cleaved PARP,  caspase-3, caspase-9, Cyt-c, PARP↑ | (164) |
|  |  |  | In vitro | RA FLS cells | 0.713–71.3 μM |  |  |  |
| 3R-(4'-hydroxyl-3'-O-β-D-glucopyranosyl phenyl)-dihydro isocoumarin (GDC) | Paniculiflorum | Stems | In vitro | RA FLS cells | 3 and 30μM | ERK、p38 MAPK, JNK, NF-κB | p-ERK 1/2, p-p38 MAPK, p-JNK 1/2, p-p65, iNOS, TNF-α, IL-6, IL-1β, CNTF, fractalkine, IFN-γ, IL-4, TIMP-1↓ | (165) |
| Scopoletin (SCO) | Erycibe obtusifolia,  Aster tataricus, Foeniculum vulgare | Root, Stems | In vitro | RA FLS cells | 15, 30 and 60μM | MAPK/PKC/CREB | p-p38 MAPK, p-ERK, p-PKC, CREB, IL-6↓ | (166) |
| Dolichosin A (DOA) | Glycine tabacina | Leguminosae | In vitro | SW982 synovial cells | 10 and 20μM | ERK, p38 MAPK, JNK, NF-κB, PI3K/AKT | p-ERK 1/2, p-p38 MAPK, p-JNK 1/2, p-p65, p-PI3K, p-AKT, TNF-α, IL-6, COX-2, MMP-3, IL-1β↓; IL-10↑ | (170) |
| Cinnamomulactone | Cinnamomum cassia | Stems | In vitro | RA FLS cells | 0.1-10μM | N/A | IL-1β, MMP-1, MMP-3↓ | (171) |
| Decursin (DEC) | Angelicae gigas | Root | In vitro | RAW264.7cells | 20, 40 and 60 μM | ERK, NF-kB | p-ERK 1/2, p-p65, IL-8, MCP-1, IL-1β, TNF-α, MMP-9, MMP-2, iNOS↓ | (172) |
| Daphnetin | Daphne odora Var. marginata | Root | In vivo | SD rats; Splenocytes | 2.25 and 4.5 mg/kg/day for 3 weeks; 0-160 μM | N/A | IL-1, TNF-α, MIF, Foxp3, IL-17, IL-6, TGF-β, IFN-γ, IL-4↓ | (173, 174, 175) |
| 4-methylesculetin (4-ME) | Artemisia annua | Stems, Leaves, Flowers | In vivo | Wistar rats | 10, 25 and 50 mg/kg/d for 4 weeks | NF-kB, AKT | p-p65, p-AKT, MMP-13, MMP-3, MMP-9, TNF-a, IL-1β, IL-6, COX-2, PGE2↓ | (176) |
| Osthole (OT) | Fructus Cnidii | Fruit | In vivo and  in vitro | Wistar rats | 20-40 mg/kg/d for 3 weeks | AMPK | p-AMPK, Mfn2, IL-10, Caspase-3↑; NLRP3, Caspase-1, IL-1β, ASC, Drp1, TNFα, Bcl-XL, CXCL10, CCL5, MCP1, IL-33, COX2, IL-6, IL-8↓ | (177) |
|  |  |  | In vitro | RA FLS cells | 25-50 μM |  |  |  |
| Umbelliferone (UMB) | carrot, angelica, coriander | Fruit, Leaves | In vivo | Wistar rats | 10, 20 and 40 mg/kg/d for 4 weeks | NF-κB | NF-κB, COX-2, PGE2, VEGF, TNF-α,IL-1β, IL-6, IL-17↓; IL-10↑ | (178) |
| Osthole (OT) | Fructus Cnidii | Fruit | In vivo | DBA1/J mice | 30 mg/kg/d for 3 weeks | miR-1224-3p/AGO1 | IL-1β, IL-6, AGO1 ↓; miR-1224-3p↑ | (182) |
|  |  |  | In vitro | RA FLS cells | 50 μM |  |  |  |
| Oxypeucedanin hydrat (OXH) | Notopterygium incisum， Angelica dahurica， Whiteflower Hogfennel | Root | In vivo | Wistar rats | 13.5 mg/kg/d for 3 weeks | ERK, p38 MAPK, JNK, TLR4-MD2/NF-κB | p-ERK 1/2, p-p38 MAPK, p-JNK 1/2, p-IκB, COX-2, iNOS, IL-6, IL-1β, TNF-α, TLR4, DMSO↓ | (183) |
|  |  |  | In vivo | Wistar rats | 13.5 mg/kg/d for 3 weeks |  |  |  |
| Oxyimperatorin (OPT) | Angelica dahurica | Root | In vivo | DBA/1 mice | 25 and 50 mg/kg/d for 3 weeks | PI3K/AKT | p-PI3K, p-AKT, CDK1, cyclin B1, survivin↓ | (186) |
|  |  |  | In vitro | RA FLS cells | 50 and 100μg/ml |  |  |  |
| 7-Hydroxycoumarin (7-HC) | Prunellae Spica,  Radix Angelicae Biseratae and Eucommiae Cortex, coriander, carrot, golden apple | Root, Fruit | In vivo | SD rats | 15, 30 and 60 mg/kg/d for 3 weeks | Wnt/β-catenin | Wnt1, LRP6, Bcl-2, IL-1β, IL-6, TNF-α, p-GSK-3β, β-catenin, cyclin D1↓; Bax, caspase 3, cleaved caspase 3↑ | (187) |
|  |  |  | In vitro | MH7A cells | 20, 40 and 80μg/ml |  |  |  |
| Urolithin A (UA) | Pomegranates, nuts | Fruit | In vivo | DBA mice | 50 mg/kg/d for 2 weeks | AMPK/NF-κB | p-p65, NLRP3, GSDMD, IL-1β, caspase-1, and ASC↓; p-AMPK↑ | (188) |
|  |  |  | In vitro | NIH/3T3 cells | 10, 20 and 40μM |  |  |  |
| Dendrocoumarin (DEN) | Dendropanax dentiger | Root, | In vitro | MH7A cells | 20 and 40μM | NF-κB, Akt, JNK | p-p65, p-AKT, p-JNK, IL-2, IL-6, IL-8, IFN-γ↓ | (189) |

**Supplementary Table S13. Mechanisms of coumarins in vivo and in vitro models of osteoarthritis.**

| Coumarin compounds | Source | Availability part | Intervention model | | Intervention dosage | Signalling pathway | Intervention target | References |
| --- | --- | --- | --- | --- | --- | --- | --- | --- |
|  |  |  | In vivo/  In vitro | Animal  /Cell |  |  |  |  |
| Psoralen | Psoralea corylifolia | Fruit | In vivo | New Zealand rabbits | 1 mg/kg/day ESC for 4 weeks | N/A | TFF1, CTSD, PGR, IL-6, TNF-α, IL-1β↓ | (194) |
|  |  |  | In vitro | Primary human chondrocytes | 12μg/ml |  |  |  |
| Scopoletin | Erycibe obtusifolia,  Aster tataricus, Foeniculum vulgare | Root, Stems | In vivo | SD rats | 200 and 400 mg/kg/day for 4 weeks | N/A | MMP-1, MMP-3, MMP-13, TNF-α, IL-1β, NO↓ | (195) |
| Esculin (ESC) | Aesculus hipcasanum (horse chestnut) | Fruit | In vivo | C57BL/6 mice | 40 mg/kg/day ESC for 4 weeks | Sirt1/NF-κB | p-p65, TNF-α, IL-6, COX-2, PGE2, NO, iNOS, MMP-13↓; Sirt1, COL II↑ | (196) |
|  |  |  | In vitro | Mice primary cartilages | 5, 10 and 20 μM |  |  |  |
| 4-methylumbelliferone (4-MU) | Umbelliferae and Asteraceae plant | Root | In vivo | C57BL / 6 and CD-1 mice | 150-250 mg/kg/day for 2 weeks; | N/A | MMP-3, MMP-13↓ | (197) |
|  |  |  | In vitro | Bovine cartilage | 4 μM |  |  |  |
| Nodakenin (NDK) | Angelica sinensis | Root | In vivo | C57BL/6 mice | 10 mg/kg/day for 8 weeks | Drp1 / ROS / NLRP3 | COX2, IL-1β, TNF-α, p-Drp1, MMP13, NLRP3↓; Collagen II↑ | (198) |
|  |  |  | In vitro | Primary mouse cartilages | 5-75 μM |  |  |  |
| Urolithin B (UB) | pomegranates, raspberries, blueberries, chestnuts | Fruit | In vivo | C57BL/6 mice | 50 mg/kg/day UB for 8 weeks | NF-κB | p-p65, iNOS, COX-2, MMP3, MMP13↓; collagen II, Aggrecan, SOX-9↑ | (199) |
|  |  |  | In vitro | Primary mouse cartilages | 5 and 10 μM |  |  |  |
| Osthole | Fructus Cnidii | Fruit | In vivo | ICR mice | 2 and 5 mg/kg/d for 4 weeks | NF-κB, HIF-2α | p65, COX-2, HIF-2α, RUNX-2, MMP-13, Syndecan IV, ADAMTS-5↓ | (200) |
| Byakangelicin (BYA) | Angelica dahurica | Root | In vivo | C57BL/6 mice | 10 mg/kg/day for 12 weeks | NF-κB | p-p65, iNOS, COX-2, TNF-α, IL-6, MMP-3, MMP-13, ADAMTS-5↓; Collagen II↑ | (202) |
|  |  |  | In vitro | Primary mouse cartilages | 0.625, 1.25 and 2.5μM |  |  |  |
| Imperatorin  (IMB) | Angelica archangelica | Root | In vivo | SD rats | 5 mg/kg/day for 6 weeks | N/A | HIF-1α, TGF-β1, TIMP1, VEGF, NLRP3, ASC, pro-caspase-1, P10↓ | (204) |
|  |  |  | In vitro | Primary rat FLS cells | 50 μM |  |  |  |
| 4-Methylumbelliferone (4-MU) | Umbelliferae and Asteraceae plant | Root | In vitro | Primary human chondrocytes | 2.25 and 4.5 mg/kg/day for 3 weeks; 8μM | NF-κB, AKT | p-NF-κB, p-AKT, MMP-1, MMP-3, IL-6↓ | (205) |
| Fraxetin (FT) | Cortex Fraxini | Bark | In vitro | Primary rat FLS | 10 and 50μM | TLR4/MyD88/NF-κB | TLR4, MyD88, NF-κB p65, TNF-α, MMP-13, caspase-3, caspase-8 ↓; IκBα, Collagen II↑ | (206) |
| Daphnetin (DAP) | Daphne odora Var. marginata | Root | In vivo | [New Zealand rabbits](https://www.sciencedirect.com/topics/pharmacology-toxicology-and-pharmaceutical-science/new-zealand-white-rabbit) | 12, 24 and 48 ng/ml/day for 8 weeks | PI3K/AKT, ERK, p38 MAPK, JNK, NF-κB | p-p38, p-ERK, p-Akt, p-PI3K, p-P65, MMP-1, TNF-α, IL-6, IL-12, MMP-3, MMP-9, MMP-13, Caspase-3, BAX↓; IL-10, Bcl-2↑ | (207) |
|  |  |  | In vitro | Primary rabbit chondrocytes | 5-50 ng/mL |  |  |  |
| Bergapten (BP) | bergamot, grapefruit | Essential oil | In vitro | Primary rat cartilages | 1–10 μm | ANP32A/ATM | TNF-α, IL-6, IL-1, MMP-13, ANP32A, ATM↑ | (208) |
| Coumestrol | clover, alfalfa, soy, spinach | Stems, Leaves, Flowers | In vitro | Primary human chondrocytes | 1, 5 and 10 μM | N/A | MMP-1, MMP-3, MMP-13, NO, iNOS, COX-2, PGE2↓; Aggrecan, Collagen II↑ | (209) |
| Psoralidin (PSD) | Psoralea corylifolia | Fruit | In vitro | Primary rat cartilages | 1, 5, 10, 15 and 20μM | NF-κB | p-p65, Bax, NO, MMP-1, MMP-13, caspase-3, caspase-9↓; Bcl-2↑ | (212) |
| Psoralen (PSO) | Psoralea corylifolia | Fruit | In vitro | New Zealand rabbits; Primary rat chondrocytes | 10 and 20 mg/kg/d for 8 weeks; 50 and 100μM | Keap1/Nrf2/ARE | cleaved-caspase3, BAX, MMP13, ADAMTS5, RUNX2, OSX↓; Keap1, Nrf2, ARE, Aggrecan, Col-II, Bcl-2↑ | (213) |
| Urolithin A (UA) | Pomegranates, nuts | Fruit | In vivo | C57BL/6J mice | 50 and 250 mg/kg/d for 8 weeks | PINK1/Parkin | MMP-3↓; PARK2, SQSTM1, MAP1LC3B, BNIP3↑ | (214) |
|  |  |  | In vitro | Primary human chondrocytes | 6.25 and 12μM |  |  |  |
| Isofraxidin (IFD) | Fraxinus, Eleutherococcus | Bark, Root | In vitro | Primary human chondrocytes | 1, 10 and 50μM | NF-κB | p-p65, p-IκB-α, COX-2, iNOS, MMP-1, MMP-3, MMP-13, ADAMTS-4, ADAMTS-5↓ | (215) |
| Hydroxypyridinone-Coumarin (HDC) | Hydroxypyridinone, Coumarin | Synthesized | In vivo | Wistar rats | 5, 10 and 15 mg/kg/d for 4 weeks | NF-κB | p-p65, p-IκB-α↓ ; CDK6, CDK4, and cyclin D1↑ | (218) |
|  |  |  | In vitro | Primary rat cartilages | 5, 30, 40 and 50 μM |  |  |  |
| Wedelolactone (WED) | Ecliptae | Leaves | In vivo | C57BL/6J mice | 50 mg/kg/d for 8 weeks | NF-κB | COX‑2, iNOS, NO, PEG2, TNF-α, IL-6, ADAMTS-5, MMP1, MMP3, MMP13↓；Collagen II, SOX9↑ | (219) |
|  |  |  | In vitro | Primary human chondrocytes | 10, 20 and 50 μM |  |  |  |
| Isofraxidin (IFD) | Fraxinus, Eleutherococcus | Bark, Root | In vivo | C57BL/6J mice | 20 mg/kg/d for 8 weeks | TLR4/MD-2 | TLR4, MD-2, p65, PGE2, IL-6, COX-2, iNOS, TNF-α, MMP-13, ADAMTS-5↓; IκBα, Collagen II, aggrecan↑ | (221) |
|  |  |  | In vitro | Primary human chondrocytes | 5, 10 and 20μM |  |  |  |
| Scoparone (SCO) | Artemisia capillaris | Stems, Leaves, Flowers | In vitro | Primary human chondrocytes | 5, 10 and 20μM | PI3K/Akt/NF-κB | p-PI3K, p-Akt, p-p65, p-IκBα, PGE2, MMP-3, MMP-13, ADAMTS-4, NO, ADAMTS-5, COX-2, iNOS↓ | (222) |
| Urolithin A (UA) | Pomegranates, nuts | Fruit | In vivo | C57BL/6J mice; | 20 mg/kg/d for 8 weeks | PI3K/Akt/NF-κB | p-PI3K, p-Akt, p-p65, p-IκBα, PGE2, IL-6, MMP-13, NO, ADAMTS-5, COX-2, iNOS↓; Collagen II↑ | (223) |
|  |  |  | In vitro | Primary human chondrocytes | 3, 10 and 30μM |  |  |  |
| Dicoumarol (DIC) | Melilotus officinalis | Stems, Leaves, Flowers | In vivo | SD rats | 10 mg/kg/d for 4 weeks | NF-κB | NF-κB, TGF-β, TIMP, Col1α, IL-1β, IL-18, NLRP3, GSDMD, cleaved-GSDMD, ASC↓ | (224) |
|  |  |  | In vitro | FLS cells | 12.5, 25 and 50μM |  |  |  |
| Imperatorin (IMP) | Angelica archangelica | Root | In vitro | Primary human chondrocytes | 10, 25 and 50μM | ERK-MAPK/AP1 | p-ERK, p-IκB-α, p-p-38, p-JNK, c-Fos, c-Jun, NO, COX-2↓ | (227) |
| Xanthotoxin (XAT) | Ammi majus | seeds | In vitro | C3H10T1/2 BMSCs | 50 and 100μM | p38 MAPK/HDAC4 | p-p38, Col10a1, RUNX2, MMP13; HDAC4 | (228) |
| Psoralen (PSO) | Psoralea corylifolia | Fruit | In vitro | Primary rat chondrocytes | 10^−7^, 10^−6^ and 10^−5^ mol/l | Wnt/β-catenin | GSK-3β↓; Wnt-4, Frizzled-2, β-catenin, cyclin D1, Col-II↑ | (230) |
| Osthole | Fructus Cnidii | Fruit | In vitro | Primary rat chondrocytes | 6.25 μM, 12.5 μM, and 25 μM | Wnt7b/β-catenin | Wnt7b, MMP13, ADMATS5, β-catenin, TCF4, PCNA, OCN, Runx2, VEGF, ALP↓; SOX-9↑ | (232) |

**Supplementary Table S14. Mechanisms of coumarins in vivo and in vitro models of osteosarcoma.**

| Coumarin compounds | Source | Availability part | Intervention model | | Intervention dosage | Signalling pathway | Intervention target | References |
| --- | --- | --- | --- | --- | --- | --- | --- | --- |
|  |  |  | In vivo/  In vitro | Animal  /Cell |  |  |  |  |
| 4-methylumbelliferone (4-MU) | Umbelliferae and Asteraceae plant | Root | In vivo | C3H/He mice | 10 mg/body for 2 weeks | Akt | p-Akt, CD44↓; Has,bcl-2↑ | (240) |
|  |  |  | In vitro | LM8 cells | 1.0 mM |  |  |  |
| Coumarin galbanic acid (GBA) | Ferula szowitsiana | Root | In vitro | MG-63 cells | 25, 50 and 100 μM | N/A | MMP-2, MMP-9, ADAM17, ADAMTS5↓ | (241) |
| Urolithin A (UA) | Pomegranates, nuts | Fruit | In vitro | MG-63 cells | 75 μM | AKT | p-AKT, MMP-2, MMP9↓ | (242) |
| 7-Geranyloxycoumarin | Citrus plants | Fruit | In vitro | MG-63 cells | 25, 50 and 100 μM | N/A | MMP-2, MMP-9↓ | (261) |
| Esculetin (ES) and Fraxetin (FT) | Cortex Fraxini | Bark | In vitro | C3H/He mice; LM8 cells | 3 and 10 mg/kg/d ES and 10 mg/kg/d FT for 5 weeks; 10, -100 μM ES and 50-100 μM FT | N/A | MMP-2, TGF-β1, VEGF, IL-10, MCP-1↓ | (245) |
| Daphnetin | Daphne odora Var. marginata | Root | In vitro | LM8 cells | 1-30 μM | N/A | RhoA, Cdc42 ↓ | (246) |
| Osthole (OT) | Fructus Cnidii | Fruit | In vitro | MG-63 cells | 0.5, 2.5 and 12.5 μM | N/A | Bcl-2↓; Caspase-3 , Bax↑ | (249) |
| Psoralen (PSO) | Psoralea corylifolia | Fruit | In vitro | MG-63 cells and U2OS cells | 80 μg/mL and 90 μg/mL | N/A | Bcl-2↓; Caspase-3 , Bax, CHOP, IREa, XBP-1s, GRP78, PERK, ATF-6↑ | (251) |
| Methoxsalen | Psoralea corylifolia, Ammi majus | Fruit | In vitro | MG-63 cells | 100 and 500 μM | N/A | Ca²⁺↑ | (252) |
| Daphnoretin (DPT) | Stellaa chamaejasme | Root | In vitro | HOS, U2-OS, MG-63 cells | 1, 2 and 4 μM | N/A | Bcl-2, Cyclin A, Cdc2, cyclin B1↓; Bax, Caspase-9, Caspase-3 ↑ | (253) |
| Osthole (OT) | Fructus Cnidii | Fruit | In vitro | MG63, SAOS-2 cells | 50, 100 and 150 μM | PTEN/Akt | p-Akt, MMP-2↓; PTEN, p21↑ | (257) |
