# Supplementary material for: Coumarins in musculoskeletal health: a systematic review
Source: Front Nutr. 2026 Sep 9;13:1737767. doi: 10.3389/fnut.2026.1737767 (PMC13597780; doi:10.3389/fnut.2026.1737767)
Supplement: Supplementary file 1 [file Supplementary_file_1.zip › Supplementary Tables S7-S10.DOCX]

Supplementary Table S7. OHAT risk-of-bias tool for in OP animal studies.

| **NO.** | **OHAT in vitro studies domains** | **Included studies** | | | | | | | | | | | | | | |
| --- | --- | --- | --- | --- | --- | --- | --- | --- | --- | --- | --- | --- | --- | --- | --- | --- |
|  |  | Tao et al. [1] | Sun et al. [2] | Wei et al. [3] | Lu et al. [4] | Hu et al. [5] | Liu et al. [6] | Yamamoto et al. [7] | Takashima et al. [8] | Sashidhara et al. [9] | Liu et al. [10] | Ham et al. [11] | Deng et al. [12] | Yan et al. [13] | Xiao et al. [14] | Wang et al. [15] |
| 1 | Was the administered dose or exposure level adequately randomized? | + + | + + | + + | + + | + + | + + | + + | + + | - ^$^ | + + | + + | - ^$^ | - ^$^ | + + | + + |
| 2 | Was allocation to study groups adequately concealed? | - ^$^ | - ^$^ | - ^$^ | - ^$^ | - ^$^ | - ^$^ | - ^$^ | - ^$^ | - ^$^ | - ^$^ | - ^$^ | - ^$^ | - ^$^ | - ^$^ | - ^$^ |
| 3 | Did the study design or analysis account for important confounding and  modifying variables? | + + | + + | + + | + + | + + | + + | + + | + + | + + | + + | + + | + + | + + | + + | + + |
| 4 | Were experimental conditions identical across study groups? | + + | + + | + + | + + | + + | + + | + + | - ^$^ | + + | + + | + + | + + | + + | + + | + + |
| 5 | Were research personnel blinded to the study group during the study? | - ^$^ | - ^$^ | - ^$^ | - ^$^ | - ^$^ | - ^$^ | - ^$^ | - ^$^ | - ^$^ | - ^$^ | - ^$^ | - ^$^ | - ^$^ | - ^$^ | - ^$^ |
| 6 | Were outcome data complete without attrition or exclusion from analysis? | + + | + + | + + | + + | + + | + + | + + | + + | + + | + + | + + | + + | + + | + + | + + |
| 7 | Can we be confident in the exposure characterization? | ++ | - ^$$^ | - ^$$$^ | - ^$$$^ | - ^$$$^ | - ^$$^ | - ^$$^ | - ^$$, θ^ | + + | + + | + + | + + | -^$$^ | + + | + + |
| 8 | Can we be confident in the outcome assessment? | + + | + + | + + | + + | + + | + + | + + | + + | + + | + + | + + | + + | + + | + + | + + |
| 9 | Were all measured outcomes reported? | + + | + + | + + | + + | + + | + + | + + | + + | + + | + + | + + | + + | + + | + + | + + |
| 10 | Were there no other potential threats to internal validity? | - ^θθ^ | - ^θθ^ | + + | + + | - ^θθ^ | - ^θθ^ | - ^θθ^ | - ^θθ^ | + + | + + | + + | - ^θθ^ | + + | + + | - ^θθ^ |
| **Overall appraisal: (Tier 1, Tier 2 or Tier 3)** | | **1** | **2** | **1** | **1** | **2** | **1** | **1** | **2** | **1** | **1** | **1** | **1** | **1** | **1** | **1** |

Note: + +, Definitely low risk; +, Probably low risk; -, Unclear/Probably high risk; - -, Definitely high risk

$No direct evidence

$$ Did not disclose the purity of coumarin used

$$$ Did not disclose the number of doses of coumarin administered.

θ No quantitative analysis

θθ No dietary information/control of coumarin

Supplementary Table S7. OHAT risk-of-bias tool for in OP animal studies (continue).

| **NO.** | **OHAT in vitro studies domains** | **Included studies** | | | | | | | | | | | | | | |
| --- | --- | --- | --- | --- | --- | --- | --- | --- | --- | --- | --- | --- | --- | --- | --- | --- |
|  |  | Kumar et al. [16] | Choudhary et al. [17] | Tang et al. [18] | Tang et al. [19] | Li et al. [20] | Li et al. [21] | Zhao et al. [22] | Kong et al. [23] | Ham et al. [24] | Dou et al. [25] | Hong et al. [26] | Zhao et al. [27] | Chen et al. [28] | Wang et al. [29] | Murakami et al. [30] |
| 1 | Was the administered dose or exposure level adequately randomized? | - ^$^ | - ^$^ | + + | - ^$^ | + + | + + | + + | + + | + + | - ^$^ | - ^$^ | - ^$^ | + + | + + | - ^$^ |
| 2 | Was allocation to study groups adequately concealed? | - ^$^ | - ^$^ | - ^$^ | - ^$^ | - ^$^ | - ^$^ | - ^$^ | - ^$^ | - ^$^ | - ^$^ | - ^$^ | - ^$^ | - ^$^ | - ^$^ | - ^$^ |
| 3 | Did the study design or analysis account for important confounding and  modifying variables? | + + | + + | + + | + + | + + | + + | + + | + + | + + | + + | + + | + + | + + | + + | + + |
| 4 | Were experimental conditions identical across study groups? | + + | + + | + + | + + | + + | + + | + + | + + | + + | + + | + + | + + | + + | + + | + + |
| 5 | Were research personnel blinded to the study group during the study? | - ^$^ | - ^$^ | - ^$^ | - ^$^ | - ^$^ | - ^$^ | - ^$^ | - ^$^ | - ^$^ | - ^$^ | - ^$^ | - ^$^ | - ^$^ | - ^$^ | - ^$^ |
| 6 | Were outcome data complete without attrition or exclusion from analysis? | + + | + + | + + | + + | + + | + + | + + | + + | + + | + + | + + | + + | + + | + + | + + |
| 7 | Can we be confident in the exposure characterization? | - ^$$^ | - ^$$^ | - ^$$^ | - ^$$^ | - ^$$^ | + + | - ^$$^ | + + | + + | - ^$$^ | + + | - ^$$^ | + + | + + | + + |
| 8 | Can we be confident in the outcome assessment? | + + | + + | + + | + + | + + | + + | + + | + + | + + | + + | + + | + + | + + | + + | + + |
| 9 | Were all measured outcomes reported? | + + | + + | + + | + + | + + | + + | + + | + + | + + | + + | + + | + + | + + | + + | + + |
| 10 | Were there no other potential threats to internal validity? | - ^θθ^ | + + | - ^θθ^ | - ^θθ^ | + + | + + | - ^θθ^ | + + | + + | + + | - ^θθ^ | - ^θθ^ | + + | + + | - ^θθ^ |
| **Overall appraisal: (Tier 1, Tier 2 or Tier 3)** | | **2** | **1** | **1** | **2** | **1** | **1** | **1** | **1** | **1** | **1** | **2** | **2** | **1** | **1** | **1** |

Note: + +, Definitely low risk; +, Probably low risk; -, Unclear/Probably high risk; - -, Definitely high risk

$No direct evidence

$$ Did not disclose the purity of coumarin used

θ No quantitative analysis

θθ No dietary information/control of coumarin

Supplementary Table S7. OHAT risk-of-bias tool for in OP animal studies (continue).

| **NO.** | **OHAT in vitro studies domains** | **Included studies** | | | | | | | | | | | |
| --- | --- | --- | --- | --- | --- | --- | --- | --- | --- | --- | --- | --- | --- |
|  |  | Xu et al. [31] | Li et al. [32] | Yang et al. [33] | Wei et al. [34] | Li et al. [35] | Zhang et al. [36] | Kwak et al. [37] | Zheng et al. [38] | Qin et al. [39] | Guo et al. [40] | Zhou et al. [41] | Si et al. [42] |
| 1 | Was the administered dose or exposure level adequately randomized? | + + | + + | + + | + + | + + | + + | - ^$^ | - ^$^ | + + | + + | + + | + + |
| 2 | Was allocation to study groups adequately concealed? | - ^$^ | - ^$^ | - ^$^ | - ^$^ | - ^$^ | - ^$^ | - ^$^ | - ^$^ | - ^$^ | - ^$^ | - ^$^ | - ^$^ |
| 3 | Did the study design or analysis account for important confounding and  modifying variables? | + + | + + | + + | + + | + + | + + | + + | + + | + + | + + | + + | + + |
| 4 | Were experimental conditions identical across study groups? | + + | + + | + + | + + | + + | + + | + + | + + | + + | + + | + + | + + |
| 5 | Were research personnel blinded to the study group during the study? | - ^$^ | - ^$^ | - ^$^ | - ^$^ | - ^$^ | - ^$^ | - ^$^ | - ^$^ | - ^$^ | - ^$^ | - ^$^ | - ^$^ |
| 6 | Were outcome data complete without attrition or exclusion from analysis? | + + | + + | + + | + + | + + | + + | + + | + + | + + | + + | + + | + + |
| 7 | Can we be confident in the exposure characterization? | - ^$$^ | - ^$$^ | + + | + + | - ^$$^ | + + | - ^$$^ | + + | + + | - ^$$^ | + + | + + |
| 8 | Can we be confident in the outcome assessment? | + + | + + | + + | + + | + + | + + | + + | + + | + + | + + | + + | + + |
| 9 | Were all measured outcomes reported? | + + | + + | + + | + + | + + | + + | + + | + + | + + | + + | + + | + + |
| 10 | Were there no other potential threats to internal validity? | - ^θθ^ | - ^θθ^ | - ^θθ^ | - ^θθ^ | + + | - ^θθ^ | - ^θθ^ | + + | - ^θθ^ | - ^θθ^ | - ^θθ^ | + + |
| **Overall appraisal: (Tier 1, Tier 2 or Tier 3)** | | **1** | **1** | **1** | **1** | **1** | **1** | **1** | **1** | **1** | **1** | **1** | **1** |

Note: + +, Definitely low risk; +, Probably low risk; -, Unclear/Probably high risk; - -, Definitely high risk

$No direct evidence

$$ Did not disclose the purity of coumarin used

θ No quantitative analysis

θθ No dietary information/control of coumarin

**References:**

1. Tao H, Li W, Zhang W, Yang C, Zhang C, Liang X, et al. Urolithin A suppresses RANKL-induced osteoclastogenesis and postmenopausal osteoporosis by, suppresses inflammation and downstream NF-κB activated pyroptosis pathways. Pharmacol Res. 2021 Dec;174:105967. doi: 10.1016/j.phrs.2021.105967

2. Sun R, Hai N, Yang B, Chen J, Li J, Li Q, et al. Pteryxin suppresses osteoclastogenesis and prevents bone loss via inhibiting the MAPK/Ca signaling pathways mediated by ROS. Biomed Pharmacother. 2023 Sep;165:114898. doi: 10.1016/j.biopha.2023.114898

3. Wei Q, Zhou Y, Hu Z, Shi Y, Ning Q, Ren K, et al. Function-oriented mechanism discovery of coumarins from Psoralea corylifolia L. in the treatment of ovariectomy-induced osteoporosis based on multi-omics analysis. J Ethnopharmacol. 2024 Jul 15;329:118130. doi: 10.1016/j.jep.2024.118130

4. Lu Y, Zhang M, Zhang J, Jiang M, Bai G. Psoralen prevents the inactivation of estradiol and treats osteoporosis via covalently targeting HSD17B2. J Ethnopharmacol. 2023 Jul 15;311:116426. doi: 10.1016/j.jep.2023.116426

5. Hu G, Wen J, Yang H, Zhang K. Isoimperatorin improves osteoporosis by increasing YBX1 expression to promote BGLAP m5C modification. Sci Rep. 2025 Mar 21;15(1):9734. doi: 10.1038/s41598-025-94215-7

6. Liu C, Chen J, Wang Z, Li Y, Zhang Y, Li G. Nodakenin Ameliorates Ovariectomy-Induced Bone Loss by Regulating Gut Microbiota. Molecules. 2024 Mar 11;29(6):1240. doi: 10.3390/molecules29061240

7. Yamamoto M, Ito Y, Fukui M, Otake K, Shoji Y, Kitao T, et al. Osteogenic Effects of KY-054, a Novel Coumarin Derivative on Femur Cortical Bone in Ovariectomized Female Rats. Biol Pharm Bull. 2023;46(10):1435-1443. doi: 10.1248/bpb.b23-00324

8. Takashima S, Morishita K, Yamamoto M, Ando M, Kawai S, Otake K, et al. A Novel Series of Coumarin Derivatives That Exert Osteoblastogenic Effects in Mesenchymal Stem Cells and Osteogenic Effects in Ovariectomized Female Rats. Chem Pharm Bull (Tokyo). 2024;72(12):1084-1104. doi: 10.1248/cpb.c24-00559

9. Sashidhara KV, Kumar M, Khedgikar V, Kushwaha P, Modukuri RK, Kumar A, et al. Discovery of coumarin-dihydropyridine hybrids as bone anabolic agents. J Med Chem. 2013 Jan 10;56(1):109-22. doi: 10.1021/jm301281e

10. Liu D, Ma L, Zheng J, Zhang Z, Zhang N, Han Z, et al. Isopsoralen Improves Glucocorticoid-induced Osteoporosis by Regulating Purine Metabolism and Promoting cGMP/PKG Pathway-mediated Osteoblast Differentiation. Curr Drug Metab. 2024;25(4):288-297. doi: 10.2174/0113892002308141240628071541

11. Ham JR, Choi RY, Yee ST, Hwang YH, Kim MJ, Lee MK. Methoxsalen supplementation attenuates bone loss and inflammatory response in ovariectomized mice. Chem Biol Interact. 2017 Dec 25;278:135-140. doi: 10.1016/j.cbi.2017.10.014

12. Deng X, Tan S, Zhu D, Sun Y, Yu J, Meng X, et al. The combined effect of oleonuezhenide and wedelolactone on proliferation and osteoblastogenesis of bone marrow mesenchymal stem cells. Phytomedicine. 2019 Dec;65:153103. doi: 10.1016/j.phymed.2019.153103

13. Yan DY, Tang J, Chen L, Wang B, Weng S, Xie Z, et al. Imperatorin promotes osteogenesis and suppresses osteoclast by activating AKT/GSK3 β/β-catenin pathways. J Cell Mol Med. 2020 Feb;24(3):2330-2341. doi: 10.1111/jcmm.14915

14. Xiao JJ, Zhao WJ, Zhang XT, Zhao WL, Wang XX, Yin SH, et al. Bergapten promotes bone marrow stromal cell differentiation into osteoblasts in vitro and in vivo. Mol Cell Biochem. 2015 Nov;409(1-2):113-22. doi: 10.1007/s11010-015-2517-9

15. Wang X, Tian Y, Liang X, Yin C, Huai Y, Zhao Y, et al. Bergamottin promotes osteoblast differentiation and bone formation via activating the Wnt/β-catenin signaling pathway. Food Funct. 2022 Mar 7;13(5):2913-2924. doi: 10.1039/d1fo02755g

16. Kumar P, Kushwaha P, Ahmad N, Maurya SW, Dev K, Khedgikar V, et al. Design and synthesis of dalbergin analogues and evaluation of anti-osteoporotic activity. Bioorg Med Chem Lett. 2017 Apr 15;27(8):1765-1775. doi: 10.1016/j.bmcl.2017.02.062

17. Choudhary D, Kushwaha P, Gautam J, Kumar P, Verma A, Kumar A, et al. Fast and long acting neoflavonoids dalbergin isolated from Dalbergia sissoo heartwood is osteoprotective in ovariectomized model of osteoporosis: Osteoprotective effect of Dalbergin. Biomed Pharmacother. 2016 Oct;83:942-957. doi: 10.1016/j.biopha.2016.08.010

18. Tang DZ, Yang F, Yang Z, Huang J, Shi Q, Chen D, et al. Psoralen stimulates osteoblast differentiation through activation of BMP signaling. Biochem Biophys Res Commun. 2011 Feb 11;405(2):256-61. doi: 10.1016/j.bbrc.2011.01.021

19. Tang CH, Yang RS, Chien MY, Chen CC, Fu WM. Enhancement of bone morphogenetic protein-2 expression and bone formation by coumarin derivatives via p38 and ERK-dependent pathway in osteoblasts. Eur J Pharmacol. 2008 Jan 28;579(1-3):40-9. doi: 10.1016/j.ejphar.2007.10.013

20. Li X, Xue C, Wang L, Tang D, Huang J, Zhao Y, et al. Osteoprotective effects of osthole in a mouse model of 5/6 nephrectomy through inhibiting osteoclast formation. Mol Med Rep. 2016 Oct;14(4):3769-76. doi: 10.3892/mmr.2016.5687

21. Li H, Deng W, Qin Q, Lin Y, Liu T, Mo G, et al. Isoimperatorin attenuates bone loss by inhibiting the binding of RANKL to RANK. Biochem Pharmacol. 2023 May;211:115502. doi: 10.1016/j.bcp.2023.115502

22. Zhao D, Wang Q, Zhao Y, Zhang H, Sha N, Tang D, et al. The naturally derived small compound Osthole inhibits osteoclastogenesis to prevent ovariectomy-induced bone loss in mice. Menopause. 2018 Dec;25(12):1459-1469. doi: 10.1097/GME.0000000000001150

23. Kong L, Ma R, Yang X, Zhu Z, Guo H, He B, et al. Psoralidin suppresses osteoclastogenesis in BMMs and attenuates LPS-mediated osteolysis by inhibiting inflammatory cytokines. Int Immunopharmacol. 2017 Oct;51:31-39. doi: 10.1016/j.intimp.2017.07.003

24. Ham JR, Choi RY, Lee HI, Lee MK. Methoxsalen and Bergapten Prevent Diabetes-Induced Osteoporosis by the Suppression of Osteoclastogenic Gene Expression in Mice. Int J Mol Sci. 2019 Mar 14;20(6):1298. doi: 10.3390/ijms20061298

25. Dou C, Chen Y, Ding N, Li N, Jiang H, Zhao C, et al. Xanthotoxin prevents bone loss in ovariectomized mice through the inhibition of RANKL-induced osteoclastogenesis. Osteoporos Int. 2016 Jul;27(7):2335-2344. doi: 10.1007/s00198-016-3496-8

26. Hong G, Zhou L, Han X, Sun P, Chen Z, He W, et al. Asiatic Acid Inhibits OVX-Induced Osteoporosis and Osteoclastogenesis Via Regulating RANKL-Mediated NF-κb and Nfatc1 Signaling Pathways. Front Pharmacol. 2020 Mar 27;11:331. doi: 10.3389/fphar.2020.00331

27. Zhao XL, Chen LF, Wang Z. Aesculin modulates bone metabolism by suppressing receptor activator of NF-κB ligand (RANKL)-induced osteoclastogenesis and transduction signals. Biochem Biophys Res Commun. 2017 Jun 17;488(1):15-21. doi: 10.1016/j.bbrc.2017.04.148

28. Chen G, Xu Q, Dai M, Liu X. Bergapten suppresses RANKL-induced osteoclastogenesis and ovariectomy-induced osteoporosis via suppression of NF-κB and JNK signaling pathways. Biochem Biophys Res Commun. 2019 Feb 5;509(2):329-334. doi: 10.1016/j.bbrc.2018.12.112

29. Wang L, Zheng S, Huang G, Sun J, Pan Y, Si Y, et al. Osthole-loaded N-octyl-O-sulfonyl chitosan micelles (NSC-OST) inhibits RANKL-induced osteoclastogenesis and prevents ovariectomy-induced bone loss in rats. J Cell Mol Med. 2020 Apr;24(7):4105-4117. doi: 10.1111/jcmm.15064

30. Murakami A, Song M, Katsumata S, Uehara M, Suzuki K, Ohigashi H. Citrus nobiletin suppresses bone loss in ovariectomized ddY mice and collagen-induced arthritis in DBA/1J mice: possible involvement of receptor activator of NF-kappaB ligand (RANKL)-induced osteoclastogenesis regulation. Biofactors. 2007;30(3):179-92. doi: 10.1002/biof.5520300305

31. Xu K, Chen Z, Hou J, Dong C, Shi C, Gao L, et al. Praeruptorin B inhibits osteoclastogenesis by targeting GSTP1 and impacting on the S-glutathionylation of IKKβ. Biomed Pharmacother. 2022 Oct;154:113529. doi: 10.1016/j.biopha.2022.113529

32. Li Y, Zhuang Q, Tao L, Zheng K, Chen S, Yang Y, et al. Urolithin B suppressed osteoclast activation and reduced bone loss of osteoporosis via inhibiting ERK/NF-κB pathway. Cell Prolif. 2022 Oct;55(10):e13291. doi: 10.1111/cpr.13291

33. Yang X, Liang J, Wang Z, Su Y, Zhan Y, Wu Z, et al. Sesamolin Protects Mice From Ovariectomized Bone Loss by Inhibiting Osteoclastogenesis and RANKL-Mediated NF-κB and MAPK Signaling Pathways. Front Pharmacol. 2021 Jun 14;12:664697. doi: 10.3389/fphar.2021.664697

34. Wei W, Peng C, Gu R, Yan X, Ye J, Xu Z, et al. Urolithin A attenuates RANKL-induced osteoclastogenesis by co-regulating the p38 MAPK and Nrf2 signaling pathway. Eur J Pharmacol. 2022 Apr 15;921:174865. doi: 10.1016/j.ejphar.2022.174865

35. Li XJ, Zhu Z, Han SL, Zhang ZL. Bergapten exerts inhibitory effects on diabetes-related osteoporosis via the regulation of the PI3K/AKT, JNK/MAPK and NF-κB signaling pathways in osteoprotegerin knockout mice. Int J Mol Med. 2016 Dec;38(6):1661-1672. doi: 10.3892/ijmm.2016.2794

36. Zhang B, Yang LL, Ding SQ, Liu JJ, Dong YH, Li YT, et al. Anti-Osteoporotic Activity of an Edible Traditional Chinese Medicine Cistanche deserticola on Bone Metabolism of Ovariectomized Rats Through RANKL/RANK/TRAF6-Mediated Signaling Pathways. Front Pharmacol. 2019 Nov 26;10:1412. doi: 10.3389/fphar.2019.01412

37. Kwak SC, Baek JM, Lee CH, Yoon KH, Lee MS, Kim JY. Umbelliferone Prevents Lipopolysaccharide-Induced Bone Loss and Suppresses RANKL-Induced Osteoclastogenesis by Attenuating Akt-c-Fos-NFATc1 Signaling. Int J Biol Sci. 2019 Sep 7;15(11):2427-2437. doi: 10.7150/ijbs.28609

38. Zheng Y, Wang X, Pan YJ, Shi XF, Yang L, Lou YL. Orientin suppresses osteoclastogenesis and ameliorates ovariectomy-induced osteoporosis via suppressing ROS production. Food Sci Nutr. 2023 Jun 28;11(9):5582-5595. doi: 10.1002/fsn3.3516

39. Qin Y, Song D, Liao S, Chen J, Xu M, Su Y, et al. Isosinensetin alleviates estrogen deficiency-induced osteoporosis via suppressing ROS-mediated NF-κB/MAPK signaling pathways. Biomed Pharmacother. 2023 Apr;160:114347. doi: 10.1016/j.biopha.2023.114347

40. Guo LY, Wang T, Ma HX, Chen S, Chang ZY, Li F. Synergistic effect of osthole and notopterol combination against Alzheimer's disease and osteoporosis by applying zebrafish AD/OP comorbidity model. Eur J Pharmacol. 2024 Sep 15;979:176829. doi: 10.1016/j.ejphar.2024.176829

41. Zhou H, Chen P, Zhao C, Zou S, Wu H, Huang C, et al. Fraxin inhibits ovariectomized-induced bone loss and osteoclastogenesis by suppressing ROS activity. Int Immunopharmacol. 2025 Feb 6;147:113871. doi: 10.1016/j.intimp.2024.113871

42. Si Z, Zhou S, Shen Z, Luan F. High-Throughput Metabolomics Discovers Metabolic Biomarkers and Pathways to Evaluating the Efficacy and Exploring Potential Mechanisms of Osthole Against Osteoporosis Based on UPLC/Q-TOF-MS Coupled With Multivariate Data Analysis. Front Pharmacol. 2020 May 20;11:741. doi: 10.3389/fphar.2020.00741

Supplementary Table S8. OHAT risk-of-bias tool for in RA animal studies.

| **NO.** | **OHAT in vitro studies domains** | **Included studies** | | | | | | | | | | | | | | |
| --- | --- | --- | --- | --- | --- | --- | --- | --- | --- | --- | --- | --- | --- | --- | --- | --- |
|  |  | Han et al. [1] | Luo et al. [2] | Sahu et al. [3] | Pan et al. [4] | Pan et al. [5] | Yao et al. [6] | Albiero et al. [7] | Shirani et al. [8] | Han et al. [9] | Cai et al. [10] | Gao et al. [11] | Tu et al. [12] | Zhai et al. [13] | Tu et al. [14] | Hemshekhar et al. [15] |
| 1 | Was the administered dose or exposure level adequately randomized? | + + | - ^$^ | + + | - ^$^ | - ^$^ | - ^$^ | - ^$^ | + + | + + | + + | + + | + + | + + | + + | - ^$^ |
| 2 | Was allocation to study groups adequately concealed? | - ^$^ | - ^$^ | - ^$^ | - ^$^ | - ^$^ | - ^$^ | - ^$^ | - ^$^ | - ^$^ | - ^$^ | - ^$^ | - ^$^ | - ^$^ | - ^$^ | - ^$^ |
| 3 | Did the study design or analysis account for important confounding and  modifying variables? | + + | + + | + + | + + | + + | + + | + + | + + | + + | + + | + + | + + | + + | + + | + + |
| 4 | Were experimental conditions identical across study groups? | + + | + + | + + | + + | + + | + + | + + | + + | + + | + + | + + | + + | + + | + + | + + |
| 5 | Were research personnel blinded to the study group during the study? | - ^$^ | - ^$^ | - ^$^ | - ^$^ | - ^$^ | - ^$^ | - ^$^ | - ^$^ | - ^$^ | - ^$^ | - ^$^ | - ^$^ | - ^$^ | - ^$^ | - ^$^ |
| 6 | Were outcome data complete without attrition or exclusion from analysis? | + + | + + | + + | + + | + + | + + | + + | + + | + + | + + | + + | + + | + + | + + | + + |
| 7 | Can we be confident in the exposure characterization? | ++ | - ^$$^ | - ^$$^ | ++ | - ^$$^ | + + | - ^$$^ | + + | + + | + + | + + | + + | + + | - ^$$^ | - ^$$^ |
| 8 | Can we be confident in the outcome assessment? | ++ | ++ | + + | + + | + + | + + | + + | + + | + + | + + | + + | + + | + + | + + | + + |
| 9 | Were all measured outcomes reported? | + + | + + | + + | + + | + + | + + | + + | + + | + + | + + | + + | + + | + + | + + | + + |
| 10 | Were there no other potential threats to internal validity? | - ^θθ^ | ++ | + + | + + | + + | + + | + + | + + | + + | + + | + + | + + | + + | + + | - ^θθ^ |
| **Overall appraisal: (Tier 1, Tier 2 or Tier 3)** | | **1** | **1** | **1** | **1** | **1** | **1** | **1** | **1** | **1** | **1** | **1** | **1** | **1** | **1** | **2** |

+ +, Definitely low risk; +, Probably low risk; -, Unclear/Probably high risk; - -, Definitely high risk

$No direct evidence

$$ Did not disclose the purity of coumarin used

θ No quantitative analysis

θθ No dietary information/control of coumarin

Supplementary Table S8. OHAT risk-of-bias tool for in RA animal studies (continue).

| **NO.** | **OHAT in vitro studies domains** | **Included studies** | | | | | | |
| --- | --- | --- | --- | --- | --- | --- | --- | --- |
|  |  | Liu et al. [16] | Jiang et al. [17] | Zhang et al. [18] | Gao et al. [19] | Cai et al. [20] | Chen et al. [21] | Wu et al. [22] |
| 1 | Was the administered dose or exposure level adequately randomized? | + + | + + | - ^$^ | + + | + + | + + | - ^$^ |
| 2 | Was allocation to study groups adequately concealed? | - ^$^ | - ^$^ | - ^$^ | - ^$^ | - ^$^ | - ^$^ | - ^$^ |
| 3 | Did the study design or analysis account for important confounding and  modifying variables? | + + | + + | + + | + + | + + | + + | + + |
| 4 | Were experimental conditions identical across study groups? | + + | + + | + + | + + | + + | + + | + + |
| 5 | Were research personnel blinded to the study group during the study? | - ^$^ | - ^$^ | - ^$^ | - ^$^ | - ^$^ | - ^$^ | - ^$^ |
| 6 | Were outcome data complete without attrition or exclusion from analysis? | + + | + + | + + | + + | + + | + + | + + |
| 7 | Can we be confident in the exposure characterization? | ++ | + + | - ^$$^ | ++ | + + | - ^$$^ | - ^$$^ |
| 8 | Can we be confident in the outcome assessment? | ++ | ++ | ++ | ++ | ++ | ++ | ++ |
| 9 | Were all measured outcomes reported? | + + | + + | + + | + + | + + | + + | + + |
| 10 | Were there no other potential threats to internal validity? | - ^θθ^ | ++ | - ^θθ^ | - ^θθ^ | + + | - ^θθ^ | - ^θθ^ |
| **Overall appraisal: (Tier 1, Tier 2 or Tier 3)** | | **1** | **1** | **1** | **1** | **1** | **1** | **2** |

Note: + +, Definitely low risk; +, Probably low risk; -, Unclear/Probably high risk; - -, Definitely high risk

$No direct evidence

$$ Did not disclose the purity of coumarin used

θ No quantitative analysis

θθ No dietary information/control of coumarin

**References:**

1. Han Y, Wang J, Li S, Li Y, Zhang Y, Zhang R, et al. Isopsoralen ameliorates rheumatoid arthritis by targeting MIF. Arthritis Res Ther. 2021 Sep 17;23(1):243. doi: 10.1186/s13075-021-02619-3

2. Luo X, Li LL, Zhang SS, Lu JL, Zeng Y, Zhang HY, et al. Therapeutic effects of total coumarins from Urtica dentata Hand on collagen-induced arthritis in Balb/c mice. J Ethnopharmacol. 2011 Nov 18;138(2):523-9. doi: 10.1016/j.jep.2011.09.050

3. Sahu D, Raghav SK, Gautam H, Das HR. A novel coumarin derivative, 8-methoxy chromen-2-one alleviates collagen induced arthritis by down regulating nitric oxide, NFκB and proinflammatory cytokines. Int Immunopharmacol. 2015 Dec;29(2):891-900. doi: 10.1016/j.intimp.2015.08.012

4. Pan R, Gao XH, Li Y, Xia YF, Dai Y. Anti-arthritic effect of scopoletin, a coumarin compound occurring in Erycibe obtusifolia Benth stems, is associated with decreased angiogenesis in synovium. Fundam Clin Pharmacol. 2010 Aug;24(4):477-90. doi: 10.1111/j.1472-8206.2009.00784.x

5. Pan R, Dai Y, Gao X, Xia Y. Scopolin isolated from Erycibe obtusifolia Benth stems suppresses adjuvant-induced rat arthritis by inhibiting inflammation and angiogenesis. Int Immunopharmacol. 2009 Jul;9(7-8):859-69. doi: 10.1016/j.intimp.2009.02.019

6. Yao R, Fu Y, Li S, Tu L, Zeng X, Kuang N. Regulatory effect of daphnetin, a coumarin extracted from Daphne odora, on the balance of Treg and Th17 in collagen-induced arthritis. Eur J Pharmacol. 2011 Nov 16;670(1):286-94. doi: 10.1016/j.ejphar.2011.08.019

7. Albiero LR, de Andrade MF, Marchi LF, Landi-Librandi AP, de Figueiredo-Rinhel ASG, Carvalho CA, et al. Immunomodulating action of the 3-phenylcoumarin derivative 6,7-dihydroxy-3-[3',4'-methylenedioxyphenyl]-coumarin in neutrophils from patients with rheumatoid arthritis and in rats with acute joint inflammation. Inflamm Res. 2020 Jan;69(1):115-130. doi: 10.1007/s00011-019-01298-w

8. Shirani K, Iranshahi M, Askari VR, Gholizadeh Z, Zadeh AA, Zeinali M, et al. Comparative evaluation of the protective effects of oral administration of auraptene and umbelliprenin against CFA-induced chronic inflammation with polyarthritis in rats. Biomed Pharmacother. 2021 Jul;139:111635. doi: 10.1016/j.biopha.2021.111635

9. Han Y, Liu C, Chen S, Sun H, Jia Z, Shi J, et al. Columbianadin ameliorates rheumatoid arthritis by attenuating synoviocyte hyperplasia through targeted vimentin to inhibit the VAV2/Rac-1 signaling pathway. J Adv Res. 2025 Aug;74:609-620. doi: 10.1016/j.jare.2024.09.030

10. Cai L, Zhou MY, Hu S, Liu FY, Wang MQ, Wang XH, et al. Umbelliferone Inhibits Migration, Invasion and Inflammation of Rheumatoid Arthritis Fibroblast-Like Synoviocytes and Relieves Adjuvant-Induced Arthritis in Rats by Blockade of Wnt/β-Catenin Signaling Pathway. Am J Chin Med. 2022;50(7):1945-1962. doi: 10.1142/S0192415X22500835

11. Gao Q, Shan J, Di L, Jiang L, Xu H. Therapeutic effects of daphnetin on adjuvant-induced arthritic rats. J Ethnopharmacol. 2008 Nov 20;120(2):259-63. doi: 10.1016/j.jep.2008.08.031

12. Tu L, Li S, Fu Y, Yao R, Zhang Z, Yang S, et al. The therapeutic effects of daphnetin in collagen-induced arthritis involve its regulation of Th17 cells. Int Immunopharmacol. 2012 Aug;13(4):417-23. doi: 10.1016/j.intimp.2012.04.001

13. Zhai KF, Duan H, Chen Y, Khan GJ, Cao WG, Gao GZ, et al. Apoptosis effects of imperatorin on synoviocytes in rheumatoid arthritis through mitochondrial/caspase-mediated pathways. Food Funct. 2018 Apr 25;9(4):2070-2079. doi: 10.1039/c7fo01748k

14. Tu Y, Tan L, Lu T, Wang K, Wang H, Han B, et al. Glytabastan B, a coumestan isolated from Glycine tabacina, alleviated synovial inflammation, osteoclastogenesis and collagen-induced arthritis through inhibiting MAPK and PI3K/AKT pathways. Biochem Pharmacol. 2022 Mar;197:114912. doi: 10.1016/j.bcp.2022.114912

15. Hemshekhar M, Sunitha K, Thushara RM, Sebastin Santhosh M, Shanmuga Sundaram M, Kemparaju K, et al. Antiarthritic and antiinflammatory propensity of 4-methylesculetin, a coumarin derivative. Biochimie. 2013 Jun;95(6):1326-35. doi: 10.1016/j.biochi.2013.02.014

16. Liu M, Huo X, Li C, Hu Y, Lei H, Wang D, et al. Oxypeucedanin hydrate alleviates rheumatoid arthritis by inhibiting the TLR4-MD2/NF-κB/MAPK signaling axis. Acta Biochim Biophys Sin (Shanghai). 2024 May 11;56(12):1789-1801. doi: 10.3724/abbs.2024076

17. Jiang X, Lu Z, Zhang Q, Yu J, Han D, Liu J, et al. Osthole: A potential AMPK agonist that inhibits NLRP3 inflammasome activation by regulating mitochondrial homeostasis for combating rheumatoid arthritis. Phytomedicine. 2023 Feb;110:154640. doi: 10.1016/j.phymed.2022.154640

18. Zhang W, Li Z, Dai Z, Chen S, Guo W, Wang Z, et al. GelMA Hydrogel as a Promising Delivery System for Osthole in the Treatment of Rheumatoid Arthritis: Targeting the miR-1224-3p/AGO1 Axis. Int J Mol Sci. 2023 Aug 25;24(17):13210. doi: 10.3390/ijms241713210

19. Gao X, Lyu S, Lin T, He J, Pan W, He J, et al. Oxyimperatorin Suppresses the Pathologies of Rheumatoid Arthritis and the Growth of Synovial Organoids Through Targeting the PI3K/AKT Pathway. Phytother Res. 2025 Aug;39(8):3526-3544. doi: 10.1002/ptr.70009

20. Cai L, Zong P, Zhou MY, Liu FY, Meng B, Liu MM, et al. 7-Hydroxycoumarin mitigates the severity of collagen-induced arthritis in rats by inhibiting proliferation and inducing apoptosis of fibroblast-like synoviocytes via suppression of Wnt/β-catenin signaling pathway. Phytomedicine. 2022 Jan;94:153841. doi: 10.1016/j.phymed.2021.153841

21. Chen H, Zhang Z, Lu C, Ding Y, Huang Z, Li M, et al. Urolithin A attenuates rheumatoid arthritis by inhibiting inflammation and pyroptosis in fibroblasts via the AMPK/NF-κB signaling pathway. Int Immunopharmacol. 2025 May 16;155:114604. doi: 10.1016/j.intimp.2025.114604

22. Wu G, Nie W, Wang Q, Hao Y, Gong S, Zheng Y, et al. Umbelliferone Ameliorates Complete Freund Adjuvant-Induced Arthritis via Reduction of NF-κB Signaling Pathway in Osteoclast Differentiation. Inflammation. 2021 Aug;44(4):1315-1329. doi: 10.1007/s10753-021-01418-x

Supplementary Table S9. OHAT risk-of-bias tool for in OA animal studies.

| **NO.** | **OHAT in vitro studies domains** | **Included studies** | | | | | | | | | | | | | | |
| --- | --- | --- | --- | --- | --- | --- | --- | --- | --- | --- | --- | --- | --- | --- | --- | --- |
|  |  | Huang et al. [1] | Wan et al. [2] | Sun et al. [3] | Tsuchiya et al. [4] | Yi et al. [5] | Zhang et al. [6] | Xue et al. [7] | Zhang et al. [8] | Wang et al. [9] | Zhang et al. [10] | Fan et al. [11] | D'Amico et al. [12] | Yang et al. [13] | Sun et al. [14] | Jin et al. [15] |
| 1 | Was the administered dose or exposure level adequately randomized? | + + | + + | + + | + + | + + | + + | + + | + + | + + | + + | + + | + + | + + | + + | - ^$^ |
| 2 | Was allocation to study groups adequately concealed? | - ^$^ | - ^$^ | - ^$^ | - ^$^ | - ^$^ | - ^$^ | - ^$^ | - ^$^ | - ^$^ | - ^$^ | - ^$^ | - ^$^ | - ^$^ | - ^$^ | - ^$^ |
| 3 | Did the study design or analysis account for important confounding and modifying variables? | + + | + + | + + | + + | + + | + + | + + | + + | + + | + + | + + | + + | + + | + + | + + |
| 4 | Were experimental conditions identical across study groups? | + + | + + | + + | + + | + + | + + | + + | + + | + + | + + | + + | + + | + + | + + | + + |
| 5 | Were research personnel blinded to the study group during the study? | - ^$^ | - ^$^ | - ^$^ | - ^$^ | - ^$^ | - ^$^ | - ^$^ | - ^$^ | - ^$^ | - ^$^ | - ^$^ | - ^$^ | - ^$^ | - ^$^ | - ^$^ |
| 6 | Were outcome data complete without attrition or exclusion from analysis? | + + | + + | + + | + + | + + | + + | + + | + + | + + | + + | + + | + + | + + | + + | + + |
| 7 | Can we be confident in the exposure characterization? | - ^$$^ | - ^$$^ | + + | - ^$$^ | + + | + + | - ^$$^ | + + | + + | - ^$$^ | - ^$$^ | - ^$$^ | - ^$$^ | - ^$$^ | + + |
| 8 | Can we be confident in the outcome assessment? | ++ | ++ | ++ | ++ | ++ | ++ | ++ | ++ | ++ | ++ | ++ | ++ | ++ | ++ | ++ |
| 9 | Were all measured outcomes reported? | + + | + + | + + | + + | + + | + + | + + | + + | + + | + + | + + | + + | + + | + + | + + |
| 10 | Were there no other potential threats to internal validity? | - ^θθ^ | ++ | - ^θθ^ | + + | + + | - ^θθ^ | - ^θθ^ | - ^θθ^ | + + | - ^θθ^ | - ^θθ^ | + + | + + | + + | - ^θθ^ |
| **Overall appraisal: (Tier 1, Tier 2 or Tier 3)** | | **1** | **1** | **1** | **1** | **1** | **1** | **1** | **1** | **1** | **1** | **1** | **1** | **1** | **1** | **1** |

+ +, Definitely low risk; +, Probably low risk; -, Unclear/Probably high risk; - -, Definitely high risk

$No direct evidence

$$ Did not disclose the purity of coumarin used

θ No quantitative analysis

θθ No dietary information/control of coumarin

Supplementary Table S9. OHAT risk-of-bias tool for in OA animal studies (continue).

| **NO.** | **OHAT in vitro studies domains** | **Included studies** | | | |
| --- | --- | --- | --- | --- | --- |
|  |  | Fu et al. [16] | Chern et al. [17] | Ge et al. [18] | Du et al. [19] |
| 1 | Was the administered dose or exposure level adequately randomized? | + + | + + | - ^$^ | - ^$^ |
| 2 | Was allocation to study groups adequately concealed? | - ^$^ | - ^$^ | - ^$^ | - ^$^ |
| 3 | Did the study design or analysis account for important confounding and modifying variables? | + + | + + | + + | + + |
| 4 | Were experimental conditions identical across study groups? | + + | + + | + + | + + |
| 5 | Were research personnel blinded to the study group during the study? | - ^$^ | - ^$^ | - ^$^ | - ^$^ |
| 6 | Were outcome data complete without attrition or exclusion from analysis? | + + | + + | + + | - ^$^ |
| 7 | Can we be confident in the exposure characterization? | + + | - ^$$^ | - ^$$^ | - ^$$^ |
| 8 | Can we be confident in the outcome assessment? | + + | + + | + + | + + |
| 9 | Were all measured outcomes reported? | + + | + + | + + | + + |
| 10 | Were there no other potential threats to internal validity? | - ^θθ^ | - ^θθ^ | + + | - ^θθ^ |
| **Overall appraisal: (Tier 1, Tier 2 or Tier 3)** | | **1** | **1** | **1** | **2** |

Note: + +, Definitely low risk; +, Probably low risk; -, Unclear/Probably high risk; - -, Definitely high risk

$No direct evidence

$$ Did not disclose the purity of coumarin used

θ No quantitative analysis

θθ No dietary information/control of coumarin

**References:**

Huang K, Wu B, Hou Z, Ahmad A, Ahmed M, Khan AA, et al. Psoralen downregulates osteoarthritis chondrocyte inflammation via an estrogen-like effect and attenuates osteoarthritis. Aging (Albany NY). 2022 Aug 24;14(16):6716-6726. doi: 10.18632/aging.204245

2. Wan Osman WN, Lau SF, Mohamed S. Scopoletin-standardized Morinda elliptica leaf extract suppressed inflammation and cartilage degradation to alleviate osteoarthritis: A preclinical study. Phytother Res. 2017 Dec;31(12):1954-1961. doi: 10.1002/ptr.5949

3. Sun T, Bai B, Wu H, Xing H, Li J. Esculin Alleviates Osteoarthritis Progression Through the Sirt1/NF-κB Pathway. Phytother Res. 2024 Dec;38(12):5793-5805. doi: 10.1002/ptr.8349

4. Tsuchiya S, Ohashi Y, Ishizuka S, Ishiguro N, O'Rourke DP, Knudson CB, et al. Suppression of murine osteoarthritis by 4-methylumbelliferone. J Orthop Res. 2020 May;38(5):1122-1131. doi: 10.1002/jor.24541

5. Yi N, Mi Y, Xu X, Li N, Chen B, Yan K, et al. Nodakenin attenuates cartilage degradation and inflammatory responses in a mice model of knee osteoarthritis by regulating mitochondrial Drp1/ROS/NLRP3 axis. Int Immunopharmacol. 2022 Dec;113(Pt A):109349. doi: 10.1016/j.intimp.2022.109349

6. Zhang J, Li K, Qiu X. Exploring causal correlations between inflammatory cytokines and knee osteoarthritis: a two-sample Mendelian randomization. Front Immunol. 2024 Apr 18;15:1362012. doi: 10.3389/fimmu.2024.1362012

7. Xue H, Zhou H, Lou Q, Yuan P, Feng Z, Qiao L, et al. Urolithin B reduces cartilage degeneration and alleviates osteoarthritis by inhibiting inflammation. Food Funct. 2024 Apr 2;15(7):3552-3565. doi: 10.1039/d3fo03793b

8. Zhang H, Ding L, Shi X, Mei W, Huang Z, Zhang L, et al. Imperatorin alleviated NLR family pyrin domain-containing 3 inflammasome cascade-induced synovial fibrosis and synovitis in rats with knee osteoarthritis. Bioengineered. 2021 Dec;12(2):12954-12964. doi: 10.1080/21655979.2021.2012949

9. Wang Q, Zhuang D, Feng W, Ma B, Qin L, Jin L. Fraxetin inhibits interleukin-1β-induced apoptosis, inflammation, and matrix degradation in chondrocytes and protects rat cartilage in vivo. Saudi Pharm J. 2020 Dec;28(12):1499-1506. doi: 10.1016/j.jsps.2020.09.016

10. Zhang X, Yao J, Wu Z, Zou K, Yang Z, Huang X, et al. Chondroprotective and antiarthritic effects of Daphnetin used in vitro and in vivo osteoarthritis models. Life Sci. 2020 Jan 1;240:116857. doi: 10.1016/j.lfs.2019.116857

11. Fan Z, Gao J, Chen Y, Chen Q, Peng Q, Le J, et al. Psoralen remodels the articular cartilage microenvironment by pharmacologically regulating the Nrf2 pathway in osteoarthritis treatment. Int Immunopharmacol. 2025 Mar 6;149:114221. doi: 10.1016/j.intimp.2025.114221

12. D'Amico D, Olmer M, Fouassier AM, Valdés P, Andreux PA, Rinsch C, et al. Urolithin A improves mitochondrial health, reduces cartilage degeneration, and alleviates pain in osteoarthritis. Aging Cell. 2022 Aug;21(8):e13662. doi: 10.1111/acel.13662

13. Yang K, Chen G, Wang X. Promotion of G1/S Transition and Inhibition of Inflammatory Cytokine Production by Hydroxypyridinone-Coumarin in Osteoarthritis Rats. Med Sci Monit. 2020 Mar 3;26:e920784. doi: 10.12659/MSM.920784

14. Sun W, Yue J, Cui Y, Feng J, He Q, Liang J, et al. Wedelolactone alleviates inflammation and cartilage degeneration by suppressing the NF-κB signaling pathway in osteoarthritis. Int Immunopharmacol. 2024 Dec 25;143(Pt 1):113359. doi: 10.1016/j.intimp.2024.113359

15. Jin J, Yu X, Hu Z, Tang S, Zhong X, Xu J, et al. Isofraxidin targets the TLR4/MD-2 axis to prevent osteoarthritis development. Food Funct. 2018 Nov 14;9(11):5641-5652. doi: 10.1039/c8fo01445k

16. Fu X, Gong LF, Wu YF, Lin Z, Jiang BJ, Wu L, et al. Urolithin A targets the PI3K/Akt/NF-κB pathways and prevents IL-1β-induced inflammatory response in human osteoarthritis: in vitro and in vivo studies. Food Funct. 2019 Sep 1;10(9):6135-6146. doi: 10.1039/c9fo01332f

17. Chern CM, Zhou H, Wang YH, Chang CL, Chiou WF, Chang WT, et al. Osthole ameliorates cartilage degradation by downregulation of NF-κB and HIF-2α pathways in an osteoarthritis murine model. Eur J Pharmacol. 2020 Jan 15;867:172799. doi: 10.1016/j.ejphar.2019.172799

18. Ge W, Zhang X, Wang Q, Mao J, Jia P, Cai J. Dicoumarol attenuates NLRP3 inflammasome activation to inhibit inflammation and fibrosis in knee osteoarthritis. Mol Med Rep. 2024 Jun;29(6):100. doi: 10.3892/mmr.2024.13224

19. Du G, Song Y, Wei L, Li L, Wang X, Xu Q, et al. Osthole Inhibits Proliferation and Induces Catabolism in Rat Chondrocytes and Cartilage Tissue. Cell Physiol Biochem. 2015;36(6):2480-93. doi: 10.1159/000430208

Supplementary Table S10. OHAT risk-of-bias tool for in OS animal studies.

| **NO.** | **OHAT in vitro studies domains** | **Included studies** | | |
| --- | --- | --- | --- | --- |
|  |  | Kimura et al. [1] | Arai et al. [2] | Wang et al. [3] |
| 1 | Was the administered dose or exposure level adequately randomized? | - ^$^ | + + | + + |
| 2 | Was allocation to study groups adequately concealed? | - ^$^ | - ^$^ | - ^$^ |
| 3 | Did the study design or analysis account for important confounding and modifying variables? | + + | + + | + + |
| 4 | Were experimental conditions identical across study groups? | + + | + + | + + |
| 5 | Were research personnel blinded to the study group during the study? | - ^$^ | - ^$^ | - ^$^ |
| 6 | Were outcome data complete without attrition or exclusion from analysis? | + + | + + | + + |
| 7 | Can we be confident in the exposure characterization? | - ^$$^ | - ^$$^ | + + |
| 8 | Can we be confident in the outcome assessment? | + + | + + | + + |
| 9 | Were all measured outcomes reported? | + + | + + | + + |
| 10 | Were there no other potential threats to internal validity? | - ^θθ^ | - ^θθ^ | - ^θθ^ |
| **Overall appraisal: (Tier 1, Tier 2 or Tier 3)** | | **2** | **1** | **1** |

Note: + +, Definitely low risk; +, Probably low risk; -, Unclear/Probably high risk; - -, Definitely high risk

$No direct evidence

$$ Did not disclose the purity of coumarin used

θ No quantitative analysis

θθ No dietary information/control of coumarin

**References:**

1. Kimura Y, Sumiyoshi M. Antitumor and antimetastatic actions of dihydroxycoumarins (esculetin or fraxetin) through the inhibition of M2 macrophage differentiation in tumor-associated macrophages and/or G1 arrest in tumor cells. Eur J Pharmacol. 2015 Jan 5;746:115-25. doi: 10.1016/j.ejphar.2014.10.048

2. Arai E, Nishida Y, Wasa J, Urakawa H, Zhuo L, Kimata K, et al. Inhibition of hyaluronan retention by 4-methylumbelliferone suppresses osteosarcoma cells in vitro and lung metastasis in vivo. Br J Cancer. 2011 Dec 6;105(12):1839-49. doi: 10.1038/bjc.2011.459

3. Wang L, Yang L, Lu Y, Chen Y, Liu T, Peng Y, et al. Osthole Induces Cell Cycle Arrest and Inhibits Migration and Invasion via PTEN/Akt Pathways in Osteosarcoma. Cell Physiol Biochem. 2016;38(6):2173-82. doi: 10.1159/000445573
