# Supplementary material for: Coumarins in musculoskeletal health: a systematic review
Source: Front Nutr. 2026 Sep 9;13:1737767. doi: 10.3389/fnut.2026.1737767 (PMC13597780; doi:10.3389/fnut.2026.1737767)
Supplement: Supplementary file 1 [file Supplementary_file_1.zip › Supplementary Tables S3-S6.DOCX]

Supplementary Table S3. OHAT risk-of-bias tool for in vitro OP studies.

| **NO.** | **OHAT in vitro studies domains** | **Included studies** | | | | | | | | | | | | | | |
| --- | --- | --- | --- | --- | --- | --- | --- | --- | --- | --- | --- | --- | --- | --- | --- | --- |
|  |  | Tao et al. [1] | Sun et al. [2] | Lu et al. [3] | Hu et al. [4] | Yamamoto et al. [5] | Takashima et al. [6] | Sashidhara et al. [7] | Huang et al. [8] | Ge et al. [9] | Na et al. [10] | Dong et al. [11] | Deng et al. [12] | Yan et al. [13] | Xiao et al. [14] | Wang et al. [15] |
| 1 | Was the administered dose or exposure level adequately randomized? | ++ | ++ | ++ | ++ | ++ | ++ | ++ | ++ | ++ | ++ | ++ | ++ | ++ | ++ | ++ |
| 2 | Was allocation to study groups adequately concealed? | - ^$^ | - ^$^ | - ^$^ | - ^$^ | - ^$^ | - ^$^ | - ^$^ | - ^$^ | - ^$^ | - ^$^ | - ^$^ | - ^$^ | - ^$^ | - ^$^ | - ^$^ |
| 3 | Were experimental conditions identical across study groups? | + + | + + | + + | + + | + + | - ^$^ | + + | + + | + + | + + | + + | + + | + + | + + | + + |
| 4 | Were research personnel blinded to the study group during the study? | - * | - * | - * | - * | - * | - * | - * | - * | - * | - * | - * | - * | - * | - * | - * |
| 5 | Were outcome data complete without attrition or exclusion from analysis? | + + | + + | + + | + + | + + | + + | + + | + + | + + | + + | + + | + + | + + | + + | + + |
| 6 | Can we be confident in the exposure characterization? | -^##^ | - ^$$^ | -^##^ | - ^$$, ##^ | - ^$$, ##^ | --^$$, **, ##^ | - ^$$, ##^ | - ^##^ | - ^$$, ##^ | - ^$$^ | + + | + + | -^$$, ##^ | + + | - ^##^ |
| 7 | Can we be confident in the outcome assessment? | + + | + + | + + | + + | + + | - ^###^ | + + | + + | + + | + + | + + | + + | + + | + + | + + |
| 8 | Were all measured outcomes reported? | + + | + + | + + | + + | + + | + + | + + | + + | + + | + + | + + | + + | + + | + + | + + |
| 9 | Were there no other potential threats to internal validity? | + + | + + | + + | + + | + + | + + | + + | + + | + + | + + | + + | + + | + + | + + | + + |
| **Overall appraisal: (Tier 1, Tier 2 or Tier 3)** | | **1** | **1** | **1** | **1** | **1** | **2** | **1** | **1** | **1** | **1** | **1** | **1** | **1** | **1** | **1** |

Note: + +, Definitely low risk; +, Probably low risk; -, Unclear/Probably high risk; - -, Definitely high risk

$No direct evidence

$$ Did not disclose the purity of coumarin used

* Did not mention about experimental blinding

** Not consistent or insufficient information of cell concentration across parameters

\# Used cell number instead of concentration in treatment

\## Did not disclose the stock preparation, storage and/or the maximal concentration of vehicle used

###No quantitative analysis

Supplementary Table S3. OHAT risk-of-bias tool for in vitro OP studies (continue) .

| **NO.** | **OHAT in vitro studies domains** | **Included studies** | | | | | | | | | | | | | | |
| --- | --- | --- | --- | --- | --- | --- | --- | --- | --- | --- | --- | --- | --- | --- | --- | --- |
|  |  | Fritz et al. [16] | Choudhary et al. [17] | Liu et al. [18] | Kuo et al. [19] | Tang et al. [20] | Ming et al. [21] | Abdallah et al. [22] | Tang et al. [23] | Tang et al. [24] | Zhai et al. [25] | Li et al. [26] | Li et al. [27] | Zhao et al. [28] | Kong et al. [29] | Yeon et al. [30] |
| 1 | Was the administered dose or exposure level adequately randomized? | ++ | ++ | ++ | ++ | ++ | ++ | ++ | ++ | ++ | ++ | ++ | ++ | ++ | ++ | ++ |
| 2 | Was allocation to study groups adequately concealed? | - ^$^ | - ^$^ | - ^$^ | - ^$^ | - ^$^ | - ^$^ | - ^$^ | - ^$^ | - ^$^ | - ^$^ | - ^$^ | - ^$^ | - ^$^ | - ^$^ | - ^$^ |
| 3 | Were experimental conditions identical across study groups? | + + | + + | + + | + + | + + | - ^$^ | + + | + + | + + | + + | + + | + + | + + | + + | + + |
| 4 | Were research personnel blinded to the study group during the study? | - * | - * | - * | - * | - * | - * | - * | - * | - * | - * | - * | - * | - * | - * | - * |
| 5 | Were outcome data complete without attrition or exclusion from analysis? | + + | + + | + + | + + | + + | + + | + + | + + | + + | + + | + + | + + | + + | + + | + + |
| 6 | Can we be confident in the exposure characterization? | --^$$, **, #, ##^ | - ^$$, ##^ | -^$$, ##^ | --^$$, **, #, ##^ | - ^$$^ | -^$$, ##^ | - ^$$^ | + + | - ^$$, ##^ | - ^##^ | - ^$$, ##^ | + + | -^$$, ##^ | + + | -^$$, ##^ |
| 7 | Can we be confident in the outcome assessment? | + + | + + | + + | + + | + + | + + | + + | + + | + + | + + | + + | + + | + + | + + | + + |
| 8 | Were all measured outcomes reported? | + + | + + | + + | + + | + + | + + | + + | + + | + + | + + | + + | + + | + + | + + | + + |
| 9 | Were there no other potential threats to internal validity? | + + | + + | + + | + + | + + | + + | + + | + + | + + | + + | + + | + + | + + | + + | + + |
| **Overall appraisal: (Tier 1, Tier 2 or Tier 3)** | | **1** | **1** | **1** | **1** | **1** | **1** | **1** | **1** | **1** | **1** | **1** | **1** | **1** | **1** | **1** |

Note: + +, Definitely low risk; +, Probably low risk; -, Unclear/Probably high risk; - -, Definitely high risk

$No direct evidence

$$ Did not disclose the purity of coumarin used

* Did not mention about experimental blinding

** Not consistent or insufficient information of cell concentration across parameters

\# Used cell number instead of concentration in treatment

\## Did not disclose the stock preparation, storage and/or the maximal concentration of vehicle used

###No quantitative analysis

Supplementary Table S3. OHAT risk-of-bias tool for in vitro OP studies (continue) .

| **NO.** | **OHAT in vitro studies domains** | **Included studies** | | | | | | | | | | | | | | |
| --- | --- | --- | --- | --- | --- | --- | --- | --- | --- | --- | --- | --- | --- | --- | --- | --- |
|  |  | Dou et al. [31] | Hong et al. [32] | Zhao et al. [33] | Chen et al. [34] | Ma et al. [35] | Wang et al. [36] | Murakami et al. [37] | Wang et al. [38] | Xu et al. [39] | Li et al. [40] | Yang et al. [41] | Wei et al. [42] | Abdallah et al. [43] | Kwak et al. [44] | Liu et al. [45] |
| 1 | Was the administered dose or exposure level adequately randomized? | ++ | ++ | ++ | ++ | ++ | ++ | ++ | ++ | ++ | ++ | ++ | ++ | ++ | ++ | ++ |
| 2 | Was allocation to study groups adequately concealed? | - ^$^ | - ^$^ | - ^$^ | - ^$^ | - ^$^ | - ^$^ | - ^$^ | - ^$^ | - ^$^ | - ^$^ | - ^$^ | - ^$^ | - ^$^ | - ^$^ | - ^$^ |
| 3 | Were experimental conditions identical across study groups? | + + | + + | + + | + + | + + | + + | + + | + + | + + | + + | + + | + + | + + | + + | + + |
| 4 | Were research personnel blinded to the study group during the study? | - * | - * | - * | - * | - * | - * | - * | - * | - * | - * | - * | - * | - * | - * | - * |
| 5 | Were outcome data complete without attrition or exclusion from analysis? | + + | + + | + + | + + | + + | + + | + + | + + | + + | + + | + + | + + | + + | + + | + + |
| 6 | Can we be confident in the exposure characterization? | -^$$, ##^ | - ^**, ##^ | - ^$$, ##^ | + + | - ^##^ | - ^**^ | - ^##^ | - ^$$^ | - ^$$^ | - ^$$^ | - ^##^ | + + | + + | - ^$$, ##^ | - ^$$, ##^ |
| 7 | Can we be confident in the outcome assessment? | + + | + + | - ^###^ | + + | + + | + + | + + | + + | + + | + + | + + | + + | + + | + + | + + |
| 8 | Were all measured outcomes reported? | + + | + + | + + | + + | + + | + + | + + | + + | + + | + + | + + | + + | + + | + + | + + |
| 9 | Were there no other potential threats to internal validity? | + + | + + | + + | + + | + + | + + | + + | + + | + + | + + | + + | + + | + + | + + | + + |
| **Overall appraisal: (Tier 1, Tier 2 or Tier 3)** | | **1** | **1** | **1** | **1** | **1** | **1** | **1** | **1** | **1** | **1** | **1** | **1** | **1** | **1** | **1** |

Note: + +, Definitely low risk; +, Probably low risk; -, Unclear/Probably high risk; - -, Definitely high risk

$No direct evidence

$$ Did not disclose the purity of coumarin used

* Did not mention about experimental blinding

** Not consistent or insufficient information of cell concentration across parameters

\# Used cell number instead of concentration in treatment

\## Did not disclose the stock preparation, storage and/or the maximal concentration of vehicle used

###No quantitative analysis

Supplementary Table S3. OHAT risk-of-bias tool for in vitro OP studies (continue) .

| **NO.** | **OHAT in vitro studies domains** | **Included studies** | | | | | | | |
| --- | --- | --- | --- | --- | --- | --- | --- | --- | --- |
|  |  | Zheng et al. [46] | Qin et al. [47] | Guo et al. [48] | He et al. [49] | Qu et al. [50] | Zhou et al. [51] | Kuo et al. [52] | Rama et al. [53] |
| 1 | Was the administered dose or exposure level adequately randomized? | ++ | ++ | ++ | ++ | ++ | ++ | ++ | ++ |
| 2 | Was allocation to study groups adequately concealed? | - ^$^ | - ^$^ | - ^$^ | - ^$^ | - ^$^ | - ^$^ | - ^$^ | - ^$^ |
| 3 | Were experimental conditions identical across study groups? | + + | + + | - ^$^ | + + | + + | + + | + + | + + |
| 4 | Were research personnel blinded to the study group during the study? | - * | - * | - * | - * | - * | - * | - * | - * |
| 5 | Were outcome data complete without attrition or exclusion from analysis? | + + | + + | + + | + + | + + | + + | + + | + + |
| 6 | Can we be confident in the exposure characterization? | -^##^ | + + | - ^##^ | - ^$$, ##^ | -^##, **^ | -^##^ | - ^$$, ##^ | -^##^ |
| 7 | Can we be confident in the outcome assessment? | + + | + + | + + | + + | + + | + + | + + | + + |
| 8 | Were all measured outcomes reported? | + + | + + | + + | + + | + + | + + | + + | + + |
| 9 | Were there no other potential threats to internal validity? | + + | + + | + + | + + | + + | + + | + + | + + |
| **Overall appraisal: (Tier 1, Tier 2 or Tier 3)** | | **1** | **1** | **1** | **1** | **1** | **1** | **1** | **1** |

Note: + +, Definitely low risk; +, Probably low risk; -, Unclear/Probably high risk; - -, Definitely high risk

$No direct evidence

$$ Did not disclose the purity of coumarin used

* Did not mention about experimental blinding

** Not consistent or insufficient information of cell concentration across parameters

\# Used cell number instead of concentration in treatment

\## Did not disclose the stock preparation, storage and/or the maximal concentration of vehicle used

###No quantitative analysis

**References:**

1. Tao H, Li W, Zhang W, Yang C, Zhang C, Liang X, et al. Urolithin A suppresses RANKL-induced osteoclastogenesis and postmenopausal osteoporosis by, suppresses inflammation and downstream NF-κB activated pyroptosis pathways. Pharmacol Res. 2021 Dec;174:105967. doi: 10.1016/j.phrs.2021.105967

2. Sun R, Hai N, Yang B, Chen J, Li J, Li Q, et al. Pteryxin suppresses osteoclastogenesis and prevents bone loss via inhibiting the MAPK/Ca signaling pathways mediated by ROS. Biomed Pharmacother. 2023 Sep;165:114898. doi: 10.1016/j.biopha.2023.114898

3. Lu Y, Zhang M, Zhang J, Jiang M, Bai G. Psoralen prevents the inactivation of estradiol and treats osteoporosis via covalently targeting HSD17B2. J Ethnopharmacol. 2023 Jul 15;311:116426. doi: 10.1016/j.jep.2023.116426

4. Hu G, Wen J, Yang H, Zhang K. Isoimperatorin improves osteoporosis by increasing YBX1 expression to promote BGLAP m5C modification. Sci Rep. 2025 Mar 21;15(1):9734. doi: 10.1038/s41598-025-94215-7

5. Yamamoto M, Ito Y, Fukui M, Otake K, Shoji Y, Kitao T, et al. Osteogenic Effects of KY-054, a Novel Coumarin Derivative on Femur Cortical Bone in Ovariectomized Female Rats. Biol Pharm Bull. 2023;46(10):1435-1443. doi: 10.1248/bpb.b23-00324

6. Takashima S, Morishita K, Yamamoto M, Ando M, Kawai S, Otake K, et al. A Novel Series of Coumarin Derivatives That Exert Osteoblastogenic Effects in Mesenchymal Stem Cells and Osteogenic Effects in Ovariectomized Female Rats. Chem Pharm Bull (Tokyo). 2024;72(12):1084-1104. doi: 10.1248/cpb.c24-00559

7. Sashidhara KV, Kumar M, Khedgikar V, Kushwaha P, Modukuri RK, Kumar A, et al. Discovery of coumarin-dihydropyridine hybrids as bone anabolic agents. J Med Chem. 2013 Jan 10;56(1):109-22. doi: 10.1021/jm301281e

8. Huang Y, Hou Q, Su H, Chen D, Luo Y, Jiang T. miR-488 negatively regulates osteogenic differentiation of bone marrow mesenchymal stem cells induced by psoralen by targeting Runx2. Mol Med Rep. 2019 Oct;20(4):3746-3754. doi: 10.3892/mmr.2019.10613

9. Ge L, Cui Y, Cheng K, Han J. Isopsoralen Enhanced Osteogenesis by Targeting AhR/ERα. Molecules. 2018 Oct 11;23(10):2600. doi: 10.3390/molecules23102600

10. Na W, Kang MK, Park SH, Kim DY, Oh SY, Oh MS, et al. Aesculetin Accelerates Osteoblast Differentiation and Matrix-Vesicle-Mediated Mineralization. Int J Mol Sci. 2021 Nov 17;22(22):12391. doi: 10.3390/ijms222212391

11. Dong P, Zhu D, Deng X, Zhang Y, Ma J, Sun X, et al. Effect of hydroxyapatite nanoparticles and wedelolactone on osteoblastogenesis from bone marrow mesenchymal stem cells. J Biomed Mater Res A. 2019 Jan;107(1):145-153. doi: 10.1002/jbm.a.36541

12. Deng X, Tan S, Zhu D, Sun Y, Yu J, Meng X, et al. The combined effect of oleonuezhenide and wedelolactone on proliferation and osteoblastogenesis of bone marrow mesenchymal stem cells. Phytomedicine. 2019 Dec;65:153103. doi: 10.1016/j.phymed.2019.153103

13. Yan DY, Tang J, Chen L, Wang B, Weng S, Xie Z, et al. Imperatorin promotes osteogenesis and suppresses osteoclast by activating AKT/GSK3 β/β-catenin pathways. J Cell Mol Med. 2020 Feb;24(3):2330-2341. doi: 10.1111/jcmm.14915

14. Xiao JJ, Zhao WJ, Zhang XT, Zhao WL, Wang XX, Yin SH, et al. Bergapten promotes bone marrow stromal cell differentiation into osteoblasts in vitro and in vivo. Mol Cell Biochem. 2015 Nov;409(1-2):113-22. doi: 10.1007/s11010-015-2517-9

15. Wang X, Tian Y, Liang X, Yin C, Huai Y, Zhao Y, et al. Bergamottin promotes osteoblast differentiation and bone formation via activating the Wnt/β-catenin signaling pathway. Food Funct. 2022 Mar 7;13(5):2913-2924. doi: 10.1039/d1fo02755g

16. Fritz DT, Jiang S, Xu J, Rogers MB. A polymorphism in a conserved posttranscriptional regulatory motif alters bone morphogenetic protein 2 (BMP2) RNA:protein interactions. Mol Endocrinol. 2006 Jul;20(7):1574-86. doi: 10.1210/me.2005-0469

17. Choudhary D, Kushwaha P, Gautam J, Kumar P, Verma A, Kumar A, et al. Fast and long acting neoflavonoids dalbergin isolated from Dalbergia sissoo heartwood is osteoprotective in ovariectomized model of osteoporosis: Osteoprotective effect of Dalbergin. Biomed Pharmacother. 2016 Oct;83:942-957. doi: 10.1016/j.biopha.2016.08.010

18. Liu MZ, Zhou DC, Liu Q, Xie FL, Xiang DX, Tang GY, et al. Osteogenesis activity of isocoumarin a through the activation of the PI3K-Akt/Erk cascade-activated BMP/RUNX2 signaling pathway. Eur J Pharmacol. 2019 Sep 5;858:172480. doi: 10.1016/j.ejphar.2019.172480

19. Kuo PL, Hsu YL, Chang CH, Chang JK. Osthole-mediated cell differentiation through bone morphogenetic protein-2/p38 and extracellular signal-regulated kinase 1/2 pathway in human osteoblast cells. J Pharmacol Exp Ther. 2005 Sep;314(3):1290-9. doi: 10.1124/jpet.105.085092

20. Tang DZ, Hou W, Zhou Q, Zhang M, Holz J, Sheu TJ, et al. Osthole stimulates osteoblast differentiation and bone formation by activation of beta-catenin-BMP signaling. J Bone Miner Res. 2010 Jun;25(6):1234-45. doi: 10.1002/jbmr.21

21. Ming LG, Zhou J, Cheng GZ, Ma HP, Chen KM. Osthol, a coumarin isolated from common cnidium fruit, enhances the differentiation and maturation of osteoblasts in vitro. Pharmacology. 2011;88(1-2):33-43. doi: 10.1159/000328776

22. Abdallah BM, Ali EM. 5'-hydroxy Auraptene stimulates osteoblast differentiation of bone marrow-derived mesenchymal stem cells via a BMP-dependent mechanism. J Biomed Sci. 2019 Jul 5;26(1):51. doi: 10.1186/s12929-019-0544-7

23. Tang DZ, Yang F, Yang Z, Huang J, Shi Q, Chen D, et al. Psoralen stimulates osteoblast differentiation through activation of BMP signaling. Biochem Biophys Res Commun. 2011 Feb 11;405(2):256-61. doi: 10.1016/j.bbrc.2011.01.021

24. Tang CH, Yang RS, Chien MY, Chen CC, Fu WM. Enhancement of bone morphogenetic protein-2 expression and bone formation by coumarin derivatives via p38 and ERK-dependent pathway in osteoblasts. Eur J Pharmacol. 2008 Jan 28;579(1-3):40-9. doi: 10.1016/j.ejphar.2007.10.013

25. Zhai Y, Li Y, Wang Y, Cui J, Feng K, Kong X, et al. Psoralidin, a prenylated coumestan, as a novel anti-osteoporosis candidate to enhance bone formation of osteoblasts and decrease bone resorption of osteoclasts. Eur J Pharmacol. 2017 Apr 15;801:62-71. doi: 10.1016/j.ejphar.2017.03.001

26. Li X, Xue C, Wang L, Tang D, Huang J, Zhao Y, et al. Osteoprotective effects of osthole in a mouse model of 5/6 nephrectomy through inhibiting osteoclast formation. Mol Med Rep. 2016 Oct;14(4):3769-76. doi: 10.3892/mmr.2016.5687

27. Zhao D, Wang Q, Zhao Y, Zhang H, Sha N, Tang D, et al. The naturally derived small compound Osthole inhibits osteoclastogenesis to prevent ovariectomy-induced bone loss in mice. Menopause. 2018 Dec;25(12):1459-1469. doi: 10.1097/GME.0000000000001150

28. Kong L, Ma R, Yang X, Zhu Z, Guo H, He B, et al. Psoralidin suppresses osteoclastogenesis in BMMs and attenuates LPS-mediated osteolysis by inhibiting inflammatory cytokines. Int Immunopharmacol. 2017 Oct;51:31-39. doi: 10.1016/j.intimp.2017.07.003

29. Yeon JT, Kim KJ, Choi SW, Moon SH, Park YS, Ryu BJ, et al. Anti-osteoclastogenic activity of praeruptorin A via inhibition of p38/Akt-c-Fos-NFATc1 signaling and PLCγ-independent Ca2+ oscillation. PLoS One. 2014 Feb 21;9(2):e88974. doi: 10.1371/journal.pone.0088974

30. Dou C, Chen Y, Ding N, Li N, Jiang H, Zhao C, et al. Xanthotoxin prevents bone loss in ovariectomized mice through the inhibition of RANKL-induced osteoclastogenesis. Osteoporos Int. 2016 Jul;27(7):2335-2344. doi: 10.1007/s00198-016-3496-8

31. Hong G, Zhou L, Han X, Sun P, Chen Z, He W, et al. Asiatic Acid Inhibits OVX-Induced Osteoporosis and Osteoclastogenesis Via Regulating RANKL-Mediated NF-κb and Nfatc1 Signaling Pathways. Front Pharmacol. 2020 Mar 27;11:331. doi: 10.3389/fphar.2020.00331

32. Zhao XL, Chen LF, Wang Z. Aesculin modulates bone metabolism by suppressing receptor activator of NF-κB ligand (RANKL)-induced osteoclastogenesis and transduction signals. Biochem Biophys Res Commun. 2017 Jun 17;488(1):15-21. doi: 10.1016/j.bbrc.2017.04.148

33. Chen G, Xu Q, Dai M, Liu X. Bergapten suppresses RANKL-induced osteoclastogenesis and ovariectomy-induced osteoporosis via suppression of NF-κB and JNK signaling pathways. Biochem Biophys Res Commun. 2019 Feb 5;509(2):329-334. doi: 10.1016/j.bbrc.2018.12.112

34. Ma Y, Wang L, Zheng S, Xu J, Pan Y, Tu P, et al. Osthole inhibits osteoclasts formation and bone resorption by regulating NF-κB signaling and NFATc1 activations stimulated by RANKL. J Cell Biochem. 2019 Sep;120(9):16052-16061. doi: 10.1002/jcb.28886

35. Wang L, Zheng S, Huang G, Sun J, Pan Y, Si Y, et al. Osthole-loaded N-octyl-O-sulfonyl chitosan micelles (NSC-OST) inhibits RANKL-induced osteoclastogenesis and prevents ovariectomy-induced bone loss in rats. J Cell Mol Med. 2020 Apr;24(7):4105-4117. doi: 10.1111/jcmm.15064

36. Murakami A, Song M, Katsumata S, Uehara M, Suzuki K, Ohigashi H. Citrus nobiletin suppresses bone loss in ovariectomized ddY mice and collagen-induced arthritis in DBA/1J mice: possible involvement of receptor activator of NF-kappaB ligand (RANKL)-induced osteoclastogenesis regulation. Biofactors. 2007;30(3):179-92. doi: 10.1002/biof.5520300305

37. Wang W, Wang B. Isofraxidin Inhibits Receptor Activator of Nuclear Factor-κB Ligand-Induced Osteoclastogenesis in Bone Marrow-Derived Macrophages Isolated from Sprague-Dawley Rats by Regulating NF-κB/NFATc1 and Akt/NFATc1 Signaling Pathways. Cell Transplant. 2021 Jan-Dec;30:963689721990321. doi: 10.1177/0963689721990321

38. Xu K, Chen Z, Hou J, Dong C, Shi C, Gao L, et al. Praeruptorin B inhibits osteoclastogenesis by targeting GSTP1 and impacting on the S-glutathionylation of IKKβ. Biomed Pharmacother. 2022 Oct;154:113529. doi: 10.1016/j.biopha.2022.113529

39. Li Y, Zhuang Q, Tao L, Zheng K, Chen S, Yang Y, et al. Urolithin B suppressed osteoclast activation and reduced bone loss of osteoporosis via inhibiting ERK/NF-κB pathway. Cell Prolif. 2022 Oct;55(10):e13291. doi: 10.1111/cpr.13291

40. Yang X, Liang J, Wang Z, Su Y, Zhan Y, Wu Z, et al. Sesamolin Protects Mice From Ovariectomized Bone Loss by Inhibiting Osteoclastogenesis and RANKL-Mediated NF-κB and MAPK Signaling Pathways. Front Pharmacol. 2021 Jun 14;12:664697. doi: 10.3389/fphar.2021.664697

41. Wei W, Peng C, Gu R, Yan X, Ye J, Xu Z, et al. Urolithin A attenuates RANKL-induced osteoclastogenesis by co-regulating the p38 MAPK and Nrf2 signaling pathway. Eur J Pharmacol. 2022 Apr 15;921:174865. doi: 10.1016/j.ejphar.2022.174865

42. Abdallah BM, Ali EM, Elsawy H, Badr GM, Abdel-Moneim AM, Alzahrani AM. The Coumarin Derivative 5'-Hydroxy Auraptene Suppresses Osteoclast Differentiation via Inhibiting MAPK and c-Fos/NFATc1 Pathways. Biomed Res Int. 2019 Dec 28;2019:9395146. doi: 10.1155/2019/9395146

43. Kwak SC, Baek JM, Lee CH, Yoon KH, Lee MS, Kim JY. Umbelliferone Prevents Lipopolysaccharide-Induced Bone Loss and Suppresses RANKL-Induced Osteoclastogenesis by Attenuating Akt-c-Fos-NFATc1 Signaling. Int J Biol Sci. 2019 Sep 7;15(11):2427-2437. doi: 10.7150/ijbs.28609

44. Liu X, Gao X, Liu Y, Liang D, Fu T, Song Y, et al. Daphnetin inhibits RANKL-induced osteoclastogenesis in vitro. J Cell Biochem. 2019 Feb;120(2):2304-2312. doi: 10.1002/jcb.27555

45. Zheng Y, Wang X, Pan YJ, Shi XF, Yang L, Lou YL. Orientin suppresses osteoclastogenesis and ameliorates ovariectomy-induced osteoporosis via suppressing ROS production. Food Sci Nutr. 2023 Jun 28;11(9):5582-5595. doi: 10.1002/fsn3.3516

46. Qin Y, Song D, Liao S, Chen J, Xu M, Su Y, et al. Isosinensetin alleviates estrogen deficiency-induced osteoporosis via suppressing ROS-mediated NF-κB/MAPK signaling pathways. Biomed Pharmacother. 2023 Apr;160:114347. doi: 10.1016/j.biopha.2023.114347

47. Guo LY, Wang T, Ma HX, Chen S, Chang ZY, Li F. Synergistic effect of osthole and notopterol combination against Alzheimer's disease and osteoporosis by applying zebrafish AD/OP comorbidity model. Eur J Pharmacol. 2024 Sep 15;979:176829. doi: 10.1016/j.ejphar.2024.176829

48. He J, Chen K, Deng T, Xie J, Zhong K, Yuan J, et al. Corrigendum: Inhibitory effects of Rhaponticin on osteoclast formation and resorption by targeting RANKL-induced NFATc1 and ROS activity. Front Pharmacol. 2023 Dec 18;14:1297863. doi: 10.3389/fphar.2023.1297863

49. Qu Z, An H, Feng M, Huang W, Wang D, Zhang Z, et al. Urolithin B suppresses osteoclastogenesis via inhibiting RANKL-induced signalling pathways and attenuating ROS activities. J Cell Mol Med. 2022 Aug;26(16):4428-4439. doi: 10.1111/jcmm.17467

50. Zhou H, Chen P, Zhao C, Zou S, Wu H, Huang C, et al. Fraxin inhibits ovariectomized-induced bone loss and osteoclastogenesis by suppressing ROS activity. Int Immunopharmacol. 2025 Feb 6;147:113871. doi: 10.1016/j.intimp.2024.113871

51. Luo J, Li L, Shi W, Xu K, Shen Y, Dai B. Oxidative stress and inflammation: roles in osteoporosis. Front Immunol. 2025 Aug 12;16:1611932. doi: 10.3389/fimmu.2025.1611932

52. Kuo PL, Huang YT, Chang CH, Chang JK. Fraxetin inhibits the induction of anti-Fas IgM, tumor necrosis factor-alpha and interleukin-1beta-mediated apoptosis by Fas pathway inhibition in human osteoblastic cell line MG-63. Int Immunopharmacol. 2006 Jul;6(7):1167-75. doi: 10.1016/j.intimp.2006.02.010

53. Rama Krishna B, Thummuri D, Naidu VGM, Ramakrishna S, Venkata Mallavadhani U. Synthesis of some novel orcinol based coumarin triazole hybrids with capabilities to inhibit RANKL-induced osteoclastogenesis through NF-κB signaling pathway. Bioorg Chem. 2018 Aug;78:94-102. doi: 10.1016/j.bioorg.2018.03.005

Supplementary Table S4. OHAT risk-of-bias tool for in vitro RA studies.

| **NO.** | **OHAT in vitro studies domains** | **Included studies** | | | | | | | | | | | | | | |
| --- | --- | --- | --- | --- | --- | --- | --- | --- | --- | --- | --- | --- | --- | --- | --- | --- |
|  |  | Han et al. [1] | Luo et al. [2] | Sahu et al. [3] | Yao et al. [4] | Albiero et al. [5] | Cai et al. [6] | Shu et al. [7] | Chen et al. [8] | Zheng et al. [9] | Zhai et al. [10] | Dou et al. [11] | Akram et al. [12] | Tu et al. [13] | Tu et al. [14] | Kim et al. [15] |
| 1 | Was the administered dose or exposure level adequately randomized? | ++ | ++ | ++ | ++ | ++ | ++ | ++ | ++ | ++ | ++ | ++ | ++ | ++ | ++ | ++ |
| 2 | Was allocation to study groups adequately concealed? | - ^$^ | - ^$^ | - ^$^ | - ^$^ | - ^$^ | - ^$^ | - ^$^ | - ^$^ | - ^$^ | - ^$^ | - ^$^ | - ^$^ | - ^$^ | - ^$^ | - ^$^ |
| 3 | Were experimental conditions identical across study groups? | + + | + + | + + | + + | + + | - ^$^ | + + | + + | + + | + + | + + | + + | + + | + + | + + |
| 4 | Were research personnel blinded to the study group during the study? | - * | - * | - * | - * | - * | - * | - * | - * | - * | - * | - * | - * | - * | - * | - * |
| 5 | Were outcome data complete without attrition or exclusion from analysis? | + + | + + | + + | + + | + + | + + | + + | + + | + + | + + | + + | + + | + + | + + | + + |
| 6 | Can we be confident in the exposure characterization? | - ^$$^ | - ^$$, ##^ | - ^$$^ | -^##^ | - ^$$^ | -^##^ | - ^$$, ##^ | - ^$$, ##^ | + + | + + | + + | - ^$$^ | -^$$^ | -^$$^ | - ^##^ |
| 7 | Can we be confident in the outcome assessment? | + + | - ^###^ | + + | + + | + + | + + | + + | + + | + + | + + | + + | + + | + + | + + | + + |
| 8 | Were all measured outcomes reported? | + + | + + | + + | + + | + + | + + | + + | + + | + + | + + | + + | + + | + + | + + | + + |
| 9 | Were there no other potential threats to internal validity? | + + | + + | + + | + + | + + | + + | + + | + + | + + | + + | + + | + + | + + | + + | + + |
| **Overall appraisal: (Tier 1, Tier 2 or Tier 3)** | | **1** | **1** | **1** | **1** | **1** | **1** | **1** | **1** | **1** | **1** | **1** | **1** | **1** | **1** | **1** |

Note: + +, Definitely low risk; +, Probably low risk; -, Unclear/Probably high risk; - -, Definitely high risk

$No direct evidence

$$ Did not disclose the purity of coumarin used

* Did not mention about experimental blinding

** Not consistent or insufficient information of cell concentration across parameters

\# Used cell number instead of concentration in treatment

\## Did not disclose the stock preparation, storage and/or the maximal concentration of vehicle used

###No quantitative analysis

Supplementary Table S4. OHAT risk-of-bias tool for in vitro RA studies (continue).

| **NO.** | **OHAT in vitro studies domains** | **Included studies** | | | | | | | |
| --- | --- | --- | --- | --- | --- | --- | --- | --- | --- |
|  |  | Liu et al. [16] | Jiang et al. [17] | Zhang et al. [18] | Gao et al. [19] | Cai et al. [20] | ~~Cai et al. [21]~~ | Chen et al. [22] | Yang et al. [23] |
| 1 | Was the administered dose or exposure level adequately randomized? | ++ | ++ | ++ | ++ | ++ | ++ | ++ | ++ |
| 2 | Was allocation to study groups adequately concealed? | - ^$^ | - ^$^ | - ^$^ | - ^$^ | - ^$^ | - ^$^ | - ^$^ | - ^$^ |
| 3 | Were experimental conditions identical across study groups? | + + | + + | + + | + + | + + | - ^$^ | + + | + + |
| 4 | Were research personnel blinded to the study group during the study? | - * | - * | - * | - * | - * | - * | - * | - * |
| 5 | Were outcome data complete without attrition or exclusion from analysis? | + + | + + | + + | + + | + + | + + | + + | + + |
| 6 | Can we be confident in the exposure characterization? | - ^$$, ##^ | - ^##^ | -- ^$$, **, #, ##^ | - ^##^ | - ^##^ | - ^##^ | - ^$$, ##^ | - ^$$, ##^ |
| 7 | Can we be confident in the outcome assessment? | + + | + + | + + | + + | + + | + + | + + | + + |
| 8 | Were all measured outcomes reported? | + + | + + | + + | + + | + + | + + | + + | + + |
| 9 | Were there no other potential threats to internal validity? | + + | + + | + + | + + | + + | + + | + + | + + |
| **Overall appraisal: (Tier 1, Tier 2 or Tier 3)** | | **1** | **1** | **1** | **1** | **1** | **1** | **1** | **1** |

Note: + +, Definitely low risk; +, Probably low risk; -, Unclear/Probably high risk; - -, Definitely high risk

$No direct evidence

$$ Did not disclose the purity of coumarin used

* Did not mention about experimental blinding

** Not consistent or insufficient information of cell concentration across parameters

\# Used cell number instead of concentration in treatment

\## Did not disclose the stock preparation, storage and/or the maximal concentration of vehicle used

###No quantitative analysis

**References:**

1. Han Y, Wang J, Li S, Li Y, Zhang Y, Zhang R, et al. Isopsoralen ameliorates rheumatoid arthritis by targeting MIF. Arthritis Res Ther. 2021 Sep 17;23(1):243. doi: 10.1186/s13075-021-02619-3

2. Luo X, Li LL, Zhang SS, Lu JL, Zeng Y, Zhang HY, et al. Therapeutic effects of total coumarins from Urtica dentata Hand on collagen-induced arthritis in Balb/c mice. J Ethnopharmacol. 2011 Nov 18;138(2):523-9. doi: 10.1016/j.jep.2011.09.050

3. Sahu D, Raghav SK, Gautam H, Das HR. A novel coumarin derivative, 8-methoxy chromen-2-one alleviates collagen induced arthritis by down regulating nitric oxide, NFκB and proinflammatory cytokines. Int Immunopharmacol. 2015 Dec;29(2):891-900. doi: 10.1016/j.intimp.2015.08.012

4. Yao R, Fu Y, Li S, Tu L, Zeng X, Kuang N. Regulatory effect of daphnetin, a coumarin extracted from Daphne odora, on the balance of Treg and Th17 in collagen-induced arthritis. Eur J Pharmacol. 2011 Nov 16;670(1):286-94. doi: 10.1016/j.ejphar.2011.08.019

5. Albiero LR, de Andrade MF, Marchi LF, Landi-Librandi AP, de Figueiredo-Rinhel ASG, Carvalho CA, et al. Immunomodulating action of the 3-phenylcoumarin derivative 6,7-dihydroxy-3-[3',4'-methylenedioxyphenyl]-coumarin in neutrophils from patients with rheumatoid arthritis and in rats with acute joint inflammation. Inflamm Res. 2020 Jan;69(1):115-130. doi: 10.1007/s00011-019-01298-w

6. Cai L, Zhou MY, Hu S, Liu FY, Wang MQ, Wang XH, et al. Umbelliferone Inhibits Migration, Invasion and Inflammation of Rheumatoid Arthritis Fibroblast-Like Synoviocytes and Relieves Adjuvant-Induced Arthritis in Rats by Blockade of Wnt/β-Catenin Signaling Pathway. Am J Chin Med. 2022;50(7):1945-1962. doi: 10.1142/S0192415X22500835

7. Shu K, Kuang N, Zhang Z, Hu Z, Zhang Y, Fu Y, et al. Therapeutic effect of daphnetin on the autoimmune arthritis through demethylation of proapoptotic genes in synovial cells. J Transl Med. 2014 Oct 14;12:287. doi: 10.1186/s12967-014-0287-x

8. Chen X, Kuang N, Zeng X, Zhang Z, Li Y, Liu W, et al. Effects of daphnetin combined with Bcl2-siRNA on antiapoptotic genes in synovial fibroblasts of rats with collagen-induced arthritis. Mol Med Rep. 2018 Jan;17(1):884-890. doi: 10.3892/mmr.2017.8008

9. Zheng M, Kuang N, Zeng X, Wang J, Zou Y, Fu Y. Daphnetin induces apoptosis in fibroblast-like synoviocytes from collagen-induced arthritic rats mainly via the mitochondrial pathway. Cytokine. 2020 Sep;133:155146. doi: 10.1016/j.cyto.2020.155146

10. Zhai KF, Duan H, Chen Y, Khan GJ, Cao WG, Gao GZ, et al. Apoptosis effects of imperatorin on synoviocytes in rheumatoid arthritis through mitochondrial/caspase-mediated pathways. Food Funct. 2018 Apr 25;9(4):2070-2079. doi: 10.1039/c7fo01748k

11. Dou Y, Tong B, Wei Z, Li Y, Xia Y, Dai Y. Scopoletin suppresses IL-6 production from fibroblast-like synoviocytes of adjuvant arthritis rats induced by IL-1β stimulation. Int Immunopharmacol. 2013 Dec;17(4):1037-43. doi: 10.1016/j.intimp.2013.10.011

12. Akram A, Ara N, Bhattarai P, Irfan M, Zhu L. MMP-Sensitive Macrophage-Targeted Coenzyme Q10 Nanomedicine for Rheumatoid Arthritis Treatment. Mol Pharm. 2025 Sep 1;22(9):5638-5651. doi: 10.1021/acs.molpharmaceut.5c00742

13. Tu Y, Tan L, Lu T, Wang K, Wang H, Han B, et al. Glytabastan B, a coumestan isolated from Glycine tabacina, alleviated synovial inflammation, osteoclastogenesis and collagen-induced arthritis through inhibiting MAPK and PI3K/AKT pathways. Biochem Pharmacol. 2022 Mar;197:114912. doi: 10.1016/j.bcp.2022.114912

14. Tu Y, Wang K, Tan L, Han B, Hu Y, Ding H, et al. Dolichosin A, a coumestan isolated from Glycine tabacina, inhibits IL-1β-induced inflammation in SW982 human synovial cells and suppresses RANKL-induced osteoclastogenesis: From network pharmacology to experimental pharmacology. J Ethnopharmacol. 2020 Aug 10;258:112855. doi: 10.1016/j.jep.2020.112855

15. Kim GJ, Lee JY, Choi HG, Kim SY, Kim E, Shim SH, et al. Cinnamomulactone, a new butyrolactone from the twigs of Cinnamomum cassia and its inhibitory activity of matrix metalloproteinases. Arch Pharm Res. 2017 Mar;40(3):304-310. doi: 10.1007/s12272-016-0877-7

16. Liu M, Huo X, Li C, Hu Y, Lei H, Wang D, et al. Oxypeucedanin hydrate alleviates rheumatoid arthritis by inhibiting the TLR4-MD2/NF-κB/MAPK signaling axis. Acta Biochim Biophys Sin (Shanghai). 2024 May 11;56(12):1789-1801. doi: 10.3724/abbs.2024076

17. Jiang X, Lu Z, Zhang Q, Yu J, Han D, Liu J, et al. Osthole: A potential AMPK agonist that inhibits NLRP3 inflammasome activation by regulating mitochondrial homeostasis for combating rheumatoid arthritis. Phytomedicine. 2023 Feb;110:154640. doi: 10.1016/j.phymed.2022.154640

18. Zhang W, Li Z, Dai Z, Chen S, Guo W, Wang Z, et al. GelMA Hydrogel as a Promising Delivery System for Osthole in the Treatment of Rheumatoid Arthritis: Targeting the miR-1224-3p/AGO1 Axis. Int J Mol Sci. 2023 Aug 25;24(17):13210. doi: 10.3390/ijms241713210

19. Gao X, Lyu S, Lin T, He J, Pan W, He J, et al. Oxyimperatorin Suppresses the Pathologies of Rheumatoid Arthritis and the Growth of Synovial Organoids Through Targeting the PI3K/AKT Pathway. Phytother Res. 2025 Aug;39(8):3526-3544. doi: 10.1002/ptr.70009

20. Cai L, Zong P, Zhou MY, Liu FY, Meng B, Liu MM, et al. 7-Hydroxycoumarin mitigates the severity of collagen-induced arthritis in rats by inhibiting proliferation and inducing apoptosis of fibroblast-like synoviocytes via suppression of Wnt/β-catenin signaling pathway. Phytomedicine. 2022 Jan;94:153841. doi: 10.1016/j.phymed.2021.153841

21. Chen H, Zhang Z, Lu C, Ding Y, Huang Z, Li M, et al. Urolithin A attenuates rheumatoid arthritis by inhibiting inflammation and pyroptosis in fibroblasts via the AMPK/NF-κB signaling pathway. Int Immunopharmacol. 2025 May 16;155:114604. doi: 10.1016/j.intimp.2025.114604

22. Yang L, Liu R, Fang Y, He J. Anti-inflammatory effect of phenylpropanoids from Dendropanax dentiger in TNF-α-induced MH7A cells via inhibition of NF-κB, Akt and JNK signaling pathways. Int Immunopharmacol. 2021 May;94:107463. doi: 10.1016/j.intimp.2021.107463

Supplementary Table S5. OHAT risk-of-bias tool for in vitro OA studies.

| **NO.** | **OHAT in vitro studies domains** | **Included studies** | | | | | | | | | | | | | | |
| --- | --- | --- | --- | --- | --- | --- | --- | --- | --- | --- | --- | --- | --- | --- | --- | --- |
|  |  | Huang et al. [1] | Wan et al. [2] | Sun et al. [3] | Tsuchiya et al. [4] | Yi et al. [5] | Zhang et al. [6] | Xue et al. [7] | Zhang et al. [8] | Idota et al. [9] | Wang et al. [10] | Zhang et al. [11] | He et al. [12] | You et al. [13] | Rao et al. [14] | Fan et al. [15] |
| 1 | Was the administered dose or exposure level adequately randomized? | ++ | ++ | ++ | ++ | ++ | ++ | ++ | ++ | ++ | ++ | ++ | ++ | ++ | ++ | ++ |
| 2 | Was allocation to study groups adequately concealed? | - ^$^ | - ^$^ | - ^$^ | - ^$^ | - ^$^ | - ^$^ | - ^$^ | - ^$^ | - ^$^ | - ^$^ | - ^$^ | - ^$^ | - ^$^ | - ^$^ | - ^$^ |
| 3 | Were experimental conditions identical across study groups? | + + | + + | + + | + + | + + | + + | + + | + + | + + | + + | + + | + + | + + | + + | + + |
| 4 | Were research personnel blinded to the study group during the study? | - * | - * | - * | - * | - * | - * | - * | - * | - * | - * | - * | - * | - * | - * | - * |
| 5 | Were outcome data complete without attrition or exclusion from analysis? | + + | + + | + + | + + | + + | + + | + + | + + | + + | + + | + + | + + | + + | + + | + + |
| 6 | Can we be confident in the exposure characterization? | - ^$$, ##^ | - ^$$^ | + + | - ^$$, ##^ | - ^##^ | - ^##^ | - ^$$, ##^ | - ^**,^ ^##^ | - ^##^ | - ^##^ | - ^$$, ##^ | - ^##^ | - ^$$, ##^ | - ^$$^ | - ^$$,^ ^#^ |
| 7 | Can we be confident in the outcome assessment? | + + | + + | + + | + + | + + | + + | + + | + + | + + | + + | + + | + + | + + | + + | + + |
| 8 | Were all measured outcomes reported? | + + | + + | + + | + + | + + | + + | + + | + + | + + | + + | + + | + + | + + | + + | + + |
| 9 | Were there no other potential threats to internal validity? | + + | + + | + + | + + | + + | + + | + + | + + | + + | + + | + + | + + | + + | + + | + + |
| **Overall appraisal: (Tier 1, Tier 2 or Tier 3)** | | **1** | **1** | **1** | **1** | **1** | **1** | **1** | **1** | **1** | **1** | **1** | **1** | **1** | **1** | **1** |

Note: + +, Definitely low risk; +, Probably low risk; -, Unclear/Probably high risk; - -, Definitely high risk

$No direct evidence

$$ Did not disclose the purity of coumarin used

* Did not mention about experimental blinding

** Not consistent or insufficient information of cell concentration across parameters

\# Used cell number instead of concentration in treatment

\## Did not disclose the stock preparation, storage and/or the maximal concentration of vehicle used

###No quantitative analysis

Supplementary Table S5. OHAT risk-of-bias tool for in vitro OA studies (continue).

| **NO.** | **OHAT in vitro studies domains** | **Included studies** | | | | | | | | | | | |
| --- | --- | --- | --- | --- | --- | --- | --- | --- | --- | --- | --- | --- | --- |
|  |  | D'Amico et al. [16] | Lin et al. [17] | Yang et al. [18] | Sun et al. [19] | Jin et al. [20] | Lu et al. [21] | Fu et al. [22] | Ge et al. [23] | Ahmadet al. [24] | Cao et al. [25] | Zheng et al. [26] | Du et al. [27] |
| 1 | Was the administered dose or exposure level adequately randomized? | ++ | ++ | ++ | ++ | ++ | ++ | ++ | ++ | ++ | ++ | ++ | ++ |
| 2 | Was allocation to study groups adequately concealed? | - ^$^ | - ^$^ | - ^$^ | - ^$^ | - ^$^ | - ^$^ | - ^$^ | - ^$^ | - ^$^ | - ^$^ | - ^$^ | - ^$^ |
| 3 | Were experimental conditions identical across study groups? | + + | + + | + + | + + | + + | + + | + + | + + | + + | + + | + + | + + |
| 4 | Were research personnel blinded to the study group during the study? | - * | - * | - * | - * | - * | - * | - * | - * | - * | - * | - * | - * |
| 5 | Were outcome data complete without attrition or exclusion from analysis? | + + | + + | + + | + + | + + | + + | + + | + + | + + | + + | + + | + + |
| 6 | Can we be confident in the exposure characterization? | - ^$$^ | - ^$$^ | - ^$$^ | - ^$$, ##^ | -^##^ | - ^$$, ##^ | + + | - ^##^ | - ^##^ | - ^$$^ | - ^**^ | - ^$$, ##^ |
| 7 | Can we be confident in the outcome assessment? | + + | + + | + + | + + | + + | + + | + + | + + | + + | + + | + + | + + |
| 8 | Were all measured outcomes reported? | + + | + + | + + | + + | + + | + + | + + | + + | + + | + + | + + | + + |
| 9 | Were there no other potential threats to internal validity? | + + | + + | + + | + + | + + | + + | + + | + + | + + | + + | + + | + + |
| **Overall appraisal: (Tier 1, Tier 2 or Tier 3)** | | **1** | **1** | **1** | **1** | **1** | **1** | **1** | **1** | **1** | **1** | **1** | **1** |

Note: + +, Definitely low risk; +, Probably low risk; -, Unclear/Probably high risk; - -, Definitely high risk

$No direct evidence

$$ Did not disclose the purity of coumarin used

* Did not mention about experimental blinding

** Not consistent or insufficient information of cell concentration across parameters

\# Used cell number instead of concentration in treatment

\## Did not disclose the stock preparation, storage and/or the maximal concentration of vehicle used

###No quantitative analysis

**References:**

1. Huang K, Wu B, Hou Z, Ahmad A, Ahmed M, Khan AA, et al. Psoralen downregulates osteoarthritis chondrocyte inflammation via an estrogen-like effect and attenuates osteoarthritis. Aging (Albany NY). 2022 Aug 24;14(16):6716-6726. doi: 10.18632/aging.204245

2. Wan Osman WN, Lau SF, Mohamed S. Scopoletin-standardized Morinda elliptica leaf extract suppressed inflammation and cartilage degradation to alleviate osteoarthritis: A preclinical study. Phytother Res. 2017 Dec;31(12):1954-1961. doi: 10.1002/ptr.5949

3. Sun T, Bai B, Wu H, Xing H, Li J. Esculin Alleviates Osteoarthritis Progression Through the Sirt1/NF-κB Pathway. Phytother Res. 2024 Dec;38(12):5793-5805. doi: 10.1002/ptr.8349

4. Tsuchiya S, Ohashi Y, Ishizuka S, Ishiguro N, O'Rourke DP, Knudson CB, et al. Suppression of murine osteoarthritis by 4-methylumbelliferone. J Orthop Res. 2020 May;38(5):1122-1131. doi: 10.1002/jor.24541

5. Yi N, Mi Y, Xu X, Li N, Chen B, Yan K, et al. Nodakenin attenuates cartilage degradation and inflammatory responses in a mice model of knee osteoarthritis by regulating mitochondrial Drp1/ROS/NLRP3 axis. Int Immunopharmacol. 2022 Dec;113(Pt A):109349. doi: 10.1016/j.intimp.2022.109349

6. Zhang J, Li K, Qiu X. Exploring causal correlations between inflammatory cytokines and knee osteoarthritis: a two-sample Mendelian randomization. Front Immunol. 2024 Apr 18;15:1362012. doi: 10.3389/fimmu.2024.1362012

7. Xue H, Zhou H, Lou Q, Yuan P, Feng Z, Qiao L, et al. Urolithin B reduces cartilage degeneration and alleviates osteoarthritis by inhibiting inflammation. Food Funct. 2024 Apr 2;15(7):3552-3565. doi: 10.1039/d3fo03793b

8. Zhang H, Ding L, Shi X, Mei W, Huang Z, Zhang L, et al. Imperatorin alleviated NLR family pyrin domain-containing 3 inflammasome cascade-induced synovial fibrosis and synovitis in rats with knee osteoarthritis. Bioengineered. 2021 Dec;12(2):12954-12964. doi: 10.1080/21655979.2021.2012949

9. Idota M, Ishizuka S, Hiraiwa H, Yamashita S, Oba H, Kawamura Y, et al. 4-Methylumbelliferone suppresses catabolic activation in anterior cruciate ligament-derived cells via a mechanism independent of hyaluronan inhibition. J Orthop Surg Res. 2021 Aug 17;16(1):507. doi: 10.1186/s13018-021-02637-6

10. Wang Q, Zhuang D, Feng W, Ma B, Qin L, Jin L. Fraxetin inhibits interleukin-1β-induced apoptosis, inflammation, and matrix degradation in chondrocytes and protects rat cartilage in vivo. Saudi Pharm J. 2020 Dec;28(12):1499-1506. doi: 10.1016/j.jsps.2020.09.016

11. Zhang X, Yao J, Wu Z, Zou K, Yang Z, Huang X, et al. Chondroprotective and antiarthritic effects of Daphnetin used in vitro and in vivo osteoarthritis models. Life Sci. 2020 Jan 1;240:116857. doi: 10.1016/j.lfs.2019.116857

12. He Y, Zisan Z, Lu Z, Zheng L, Zhao J. Bergapten alleviates osteoarthritis by regulating the ANP32A/ATM signaling pathway. FEBS Open Bio. 2019 Jun;9(6):1144-1152. doi: 10.1002/2211-5463.12648

13. You JS, Cho IA, Kang KR, Oh JS, Yu SJ, Lee GJ, et al. Coumestrol Counteracts Interleukin-1β-Induced Catabolic Effects by Suppressing Inflammation in Primary Rat Chondrocytes. Inflammation. 2017 Feb;40(1):79-91. doi: 10.1007/s10753-016-0455-7

14. Rao Z, Wang S, Wang J. Protective effects of psoralidin on IL-1β-induced chondrocyte apoptosis. Mol Med Rep. 2018 Feb;17(2):3418-3424. doi: 10.3892/mmr.2017.8248

15. Fan Z, Gao J, Chen Y, Chen Q, Peng Q, Le J, et al. Psoralen remodels the articular cartilage microenvironment by pharmacologically regulating the Nrf2 pathway in osteoarthritis treatment. Int Immunopharmacol. 2025 Mar 6;149:114221. doi: 10.1016/j.intimp.2025.114221

16. D'Amico D, Olmer M, Fouassier AM, Valdés P, Andreux PA, Rinsch C, et al. Urolithin A improves mitochondrial health, reduces cartilage degeneration, and alleviates pain in osteoarthritis. Aging Cell. 2022 Aug;21(8):e13662. doi: 10.1111/acel.13662

17. Lin J, Li X, Qi W, Yan Y, Chen K, Xue X, et al. Isofraxidin inhibits interleukin-1β induced inflammatory response in human osteoarthritis chondrocytes. Int Immunopharmacol. 2018 Nov;64:238-245. doi: 10.1016/j.intimp.2018.09.003

18. Yang K, Chen G, Wang X. Promotion of G1/S Transition and Inhibition of Inflammatory Cytokine Production by Hydroxypyridinone-Coumarin in Osteoarthritis Rats. Med Sci Monit. 2020 Mar 3;26:e920784. doi: 10.12659/MSM.920784

19. Sun W, Yue J, Cui Y, Feng J, He Q, Liang J, et al. Wedelolactone alleviates inflammation and cartilage degeneration by suppressing the NF-κB signaling pathway in osteoarthritis. Int Immunopharmacol. 2024 Dec 25;143(Pt 1):113359. doi: 10.1016/j.intimp.2024.113359

20. Jin J, Yu X, Hu Z, Tang S, Zhong X, Xu J, et al. Isofraxidin targets the TLR4/MD-2 axis to prevent osteoarthritis development. Food Funct. 2018 Nov 14;9(11):5641-5652. doi: 10.1039/c8fo01445k

21. Lu C, Li Y, Hu S, Cai Y, Yang Z, Peng K. Scoparone prevents IL-1β-induced inflammatory response in human osteoarthritis chondrocytes through the PI3K/Akt/NF-κB pathway. Biomed Pharmacother. 2018 Oct;106:1169-1174. doi: 10.1016/j.biopha.2018.07.062

22. Fu X, Gong LF, Wu YF, Lin Z, Jiang BJ, Wu L, et al. Urolithin A targets the PI3K/Akt/NF-κB pathways and prevents IL-1β-induced inflammatory response in human osteoarthritis: in vitro and in vivo studies. Food Funct. 2019 Sep 1;10(9):6135-6146. doi: 10.1039/c9fo01332f

23. Ge W, Zhang X, Wang Q, Mao J, Jia P, Cai J. Dicoumarol attenuates NLRP3 inflammasome activation to inhibit inflammation and fibrosis in knee osteoarthritis. Mol Med Rep. 2024 Jun;29(6):100. doi: 10.3892/mmr.2024.13224

24. Ahmad N, Ansari MY, Bano S, Haqqi TM. Imperatorin suppresses IL-1β-induced iNOS expression via inhibiting ERK-MAPK/AP1 signaling in primary human OA chondrocytes. Int Immunopharmacol. 2020 Aug;85:106612. doi: 10.1016/j.intimp.2020.106612

25. Cao Z, Bai Y, Liu C, Dou C, Li J, Xiang J, et al. Hypertrophic differentiation of mesenchymal stem cells is suppressed by xanthotoxin via the p38-MAPK/HDAC4 pathway. Mol Med Rep. 2017 Sep;16(3):2740-2746. doi: 10.3892/mmr.2017.6886

26. Zheng W, Lin P, Ma Y, Shao X, Chen H, Chen D, et al. Psoralen promotes the expression of cyclin D1 in chondrocytes via the Wnt/β-catenin signaling pathway. Int J Mol Med. 2017 Nov;40(5):1377-1384. doi: 10.3892/ijmm.2017.3148

27. Du G, Song Y, Wei L, Li L, Wang X, Xu Q, et al. Osthole Inhibits Proliferation and Induces Catabolism in Rat Chondrocytes and Cartilage Tissue. Cell Physiol Biochem. 2015;36(6):2480-93. doi: 10.1159/000430208

Supplementary Table S6. OHAT risk-of-bias tool for in vitro OS studies.

| **NO.** | **OHAT in vitro studies domains** | **Included studies** | | | | | | | | | | |
| --- | --- | --- | --- | --- | --- | --- | --- | --- | --- | --- | --- | --- |
|  |  | Ahmadi et al. [1] | Ahmadi et al. [2] | Hosseini et al. [3] | Kimura et al. [4] | Arai et al. [5] | Fukuda et al. [6] | Dinget al. [7] | Li et al. [8] | Lu et al. [9] | Gu et al. [10] | Wang et al. [11] |
| 1 | Was the administered dose or exposure level adequately randomized? | ++ | ++ | ++ | ++ | ++ | ++ | ++ | ++ | ++ | ++ | ++ |
| 2 | Was allocation to study groups adequately concealed? | - ^$^ | - ^$^ | - ^$^ | - ^$^ | - ^$^ | - ^$^ | - ^$^ | - ^$^ | - ^$^ | - ^$^ | - ^$^ |
| 3 | Were experimental conditions identical across study groups? | + + | + + | + + | + + | + + | + + | + + | + + | + + | + + | + + |
| 4 | Were research personnel blinded to the study group during the study? | - * | - * | - * | - * | - * | - * | - * | - * | - * | - * | - * |
| 5 | Were outcome data complete without attrition or exclusion from analysis? | + + | + + | + + | + + | + + | + + | + + | + + | + + | + + | + + |
| 6 | Can we be confident in the exposure characterization? | - ^$$^ | - ^$$, **^ | - ^$$, ##^ | - ^$$, ##^ | - ^$$^ | - ^$$^ | - ^$$, ##^ | - ^$$, ##^ | - ^$$, ##^ | - ^$$, ##^ | - ^##^ |
| 7 | Can we be confident in the outcome assessment? | + + | + + | + + | + + | + + | + + | + + | + + | + + | + + | + + |
| 8 | Were all measured outcomes reported? | + + | + + | + + | + + | + + | + + | + + | + + | + + | + + | + + |
| 9 | Were there no other potential threats to internal validity? | + + | + + | + + | + + | + + | + + | + + | + + | + + | + + | + + |
| **Overall appraisal: (Tier 1, Tier 2 or Tier 3)** | | **1** | **1** | **1** | **1** | **1** | **1** | **1** | **1** | **1** | **1** | **1** |

Note: + +, Definitely low risk; +, Probably low risk; -, Unclear/Probably high risk; - -, Definitely high risk

$No direct evidence

$$ Did not disclose the purity of coumarin used

* Did not mention about experimental blinding

** Not consistent or insufficient information of cell concentration across parameters

\# Used cell number instead of concentration in treatment

\## Did not disclose the stock preparation, storage and/or the maximal concentration of vehicle used

###No quantitative analysis

**References:**

1. Ahmadi A, Hosseini F, Sarvi ZN, Iranshahi M, Rassouli FB. Harnessing the metastatic potential of human osteosarcoma cells by natural coumarin galbanic acid. Naunyn Schmiedebergs Arch Pharmacol. 2025 Sep;398(9):12633-12646. doi: 10.1007/s00210-025-04128-5

2. Ahmadi A, Hosseini F, Iranshahy M, Rassouli FB. Urolithin A suppressed osteosarcoma cell migration and invasion via targeting MMPs and AKT1. Sci Rep. 2025 Jul 17;15(1):25941. doi: 10.1038/s41598-025-11804-2

3. Hosseini F, Ahmadi A, Sarvi ZN, Iranshahi M, Rassouli FB. 7-Geranyloxycoumarin modulated metastatic potential of osteosarcoma cells via interaction with MMPs and JAK1/2. Naunyn Schmiedebergs Arch Pharmacol. 2025 Aug;398(8):10139-10153. doi: 10.1007/s00210-025-03847-z

4. Kimura Y, Sumiyoshi M. Antitumor and antimetastatic actions of dihydroxycoumarins (esculetin or fraxetin) through the inhibition of M2 macrophage differentiation in tumor-associated macrophages and/or G1 arrest in tumor cells. Eur J Pharmacol. 2015 Jan 5;746:115-25. doi: 10.1016/j.ejphar.2014.10.048

5. Arai E, Nishida Y, Wasa J, Urakawa H, Zhuo L, Kimata K, et al. Inhibition of hyaluronan retention by 4-methylumbelliferone suppresses osteosarcoma cells in vitro and lung metastasis in vivo. Br J Cancer. 2011 Dec 6;105(12):1839-49. doi: 10.1038/bjc.2011.459

6. Fukuda H, Nakamura S, Chisaki Y, Takada T, Toda Y, Murata H, et al. Daphnetin inhibits invasion and migration of LM8 murine osteosarcoma cells by decreasing RhoA and Cdc42 expression. Biochem Biophys Res Commun. 2016 Feb 26;471(1):63-7. doi: 10.1016/j.bbrc.2016.01.179

7. Ding Y, Lu X, Hu X, Ma J, Ding H. Osthole inhibits proliferation and induces apoptosis in human osteosarcoma cells. Int J Clin Pharmacol Ther. 2014 Feb;52(2):112-7. doi: 10.5414/CP201923

8. Li S, Tu H. Psoralen inhibits the proliferation and promotes apoptosis through endoplasmic reticulum stress in human osteosarcoma cells. Folia Histochem Cytobiol. 2022;60(1):101-109. doi: 10.5603/FHC.a2022.0010

9. Lu YC, Chou CT, Liang WZ, Kuo CC, Hsu SS, Wang JL, et al. Effect of Methoxsalen on Ca²⁺ Homeostasis and Viability in Human Osteosarcoma Cells. Chin J Physiol. 2017 Jun 30;60(3):174-182. doi: 10.4077/CJP.2017.BAF482

10. Gu S, He J. Daphnoretin induces cell cycle arrest and apoptosis in human osteosarcoma (HOS) cells. Molecules. 2012 Jan 9;17(1):598-612. doi: 10.3390/molecules17010598

11. Wang L, Yang L, Lu Y, Chen Y, Liu T, Peng Y, et al. Osthole Induces Cell Cycle Arrest and Inhibits Migration and Invasion via PTEN/Akt Pathways in Osteosarcoma. Cell Physiol Biochem. 2016;38(6):2173-82. doi: 10.1159/000445573
