# Supplementary material for: Coumarins in musculoskeletal health: a systematic review
Source: Front Nutr. 2026 Sep 9;13:1737767. doi: 10.3389/fnut.2026.1737767 (PMC13597780; doi:10.3389/fnut.2026.1737767)
Supplement: Supplementary file 1 [file Supplementary_file_1.zip › Supplementary Table S2.DOCX]

Supplementary Table 2. PICO framework for screening of included preclinical studies of musculoskeletal diseases (MSDs).

| PICO FRAMEWORK | | Musculoskeletal Diseases | | | |
| --- | --- | --- | --- | --- | --- |
|  |  | Osteoporosis | Rheumatoid arthritis | Osteoarthritis | Osteosarcoma |
| Population | In Vivo Models | In vivo models include rats, mice, and zebrafish. Female rats simulating age-related bone loss include SD (Sprague-Dawley) and F344/NSlc (Fischer 344) rats (used for studying early skeletal growth). Young and aged female mice include C57BL/6, BALB/c, KM (Kunming mice), ICR, C3H/HeN, and DBA/1J mice, as well as AB strain zebrafish. | In vivo study populations involve rat and mouse models (e.g., intraplantar injection of proinflammatory substances). Rats: Wistar and SD (Sprague-Dawley); mice: DBA/1J, DBA/1, DBA, Balb/c. | In vivo study populations involve rat, mouse, and rabbit models (e.g., ACLT, DMM, and intraplantar injection of proinflammatory substances). Rats: Wistar and SD (Sprague-Dawley); mice: C57BL/6, C57BL/6J, CD-1, and ICR; rabbits: New Zealand rabbits. | In vivo study populations involve C3H/He mouse models (e.g., intraplantar injection of tumor substances). |
|  | In Vitro Models | In vitro studies include primary cells and cell lines. Primary cells include BMMs (bone marrow-derived macrophages), BMSCs (bone marrow mesenchymal stem cells), primary calvarial osteoblasts, and primary osteoblastic cells. Cell lines include MC3T3-E1 (mouse preosteoblast cells), RAW 264.7 (mouse macrophages), MG-63 (human osteosarcoma cells), U937 (human monocytes), ST2 (mouse bone marrow stromal cells), HepG2 (human hepatocellular carcinoma cells), and 293T (human embryonic kidney cells). | In vitro studies include synoviocytes/fibroblast-like synoviocytes: RA FLS (rheumatoid arthritis fibroblast-like synoviocytes), MH7A (human rheumatoid arthritis synoviocyte cell line), SW982 (human synovial sarcoma cell line); macrophage/osteoclast-related cells: RAW264.7 (mouse macrophages), J774 (mouse macrophage cell line); other cells: NIH/3T3 (mouse embryonic fibroblasts), splenocytes. | In vitro studies include primary chondrocytes: primary human chondrocytes, primary mouse cartilages, primary rat cartilages, primary rabbit chondrocytes, primary rat chondrocytes; primary synoviocytes: primary rat FLS cells (primary rat fibroblast-like synoviocytes), primary rat FLS; cell lines: C3H10T1/2 BMSCs (mouse embryonic mesenchymal stem cell line). | In vitro studies include human osteosarcoma cell lines: MG-63, U2OS (U2-OS), SAOS-2, HOS; and mouse osteosarcoma cell lines: LM8. |
| Intervention/  Exposure | | Study exposures or interventions considered coumarin intake or specific coumarin-type extracts, including simple coumarins (e.g., Coumarin, Osthole, Umbelliferone, 7-Hydroxycoumarin, 4-Methylumbelliferone, 4-Methylesculetin, 7-Geranyloxycoumarin, 8-Methoxychromen-2-one, 5′-Hydroxyauraptene, Auraptene, Orcinol, Scoparone, Dicoumarol, Fraxin, Fraxetin, Esculin, Aesculin, Aesculetin, Scopoletin, Scopolin, Daphnetin, Isofraxidin, Notopterol, GDC, and Osthole-loaded N-octyl-O-sulfonyl chitosan micelles), linear furanocoumarins (e.g., Psoralen, Isopsoralen, Bergapten, Methoxsalen, Xanthotoxin, Oxypeucedanin hydrate, Oxyimperatorin, Byakangelicin, Bergamottin, Imperatorin, and Isoimperatorin), linear pyranocoumarins (e.g., Praeruptorin A, Praeruptorin B, Decursin, Nodakenin, and Columbianadin), angular pyranocoumarins (e.g., Seselin, Xanthyletin, and Dendrocoumarin), isocoumarins (e.g., Isocoumarin A, GDC, 3-Phenylcoumarin derivative, and Hydroxypyridinone-coumarin), benzocoumarins (e.g., Coumestrol and Wedelolactone), urolithins (e.g., Urolithin A and Urolithin B), as well as other coumarin-like compounds (e.g., Pteryxin, Psoralidin, Urtica dentata Hand, Glytabastan B, Dolichosin A, Cinnamomulactone, Umbelliprenin, and Daphnoretin). | | | |
| Comparators | | Controls used in these studies included vehicle control groups and groups without any intervention or exposure. Positive controls employed different doses or standard drug treatments, such as estradiol as a positive control in animal models of osteoporosis (OP), methotrexate as a positive control in animal models of rheumatoid arthritis (RA), and celecoxib as a positive control in animal models of osteoarthritis (OA). | | | |
| Outcomes | Primary outcomes | Areal bone mineral density (aBMD) measured by DXA at the lumbar spine, total hip, femoral neck, and whole body; bone microarchitecture parameters such as bone volume fraction (BV/TV), trabecular thickness (Tb.Th), trabecular number (Tb.N), and trabecular separation (Tb.Sp) | Bone/cartilage microarchitecture parameters (e.g., bone volume fraction (BV/TV), trabecular thickness (Tb.Th), trabecular number (Tb.N), and trabecular separation (Tb.Sp)); arthritis swelling index | Bone microarchitecture parameters (e.g., bone volume fraction (BV/TV), trabecular thickness (Tb.Th), trabecular number (Tb.N), and trabecular separation (Tb.Sp)); pain behavior scores (e.g., von Frey test) | Tumor migration/invasion capacity; survival rate |
|  | Secondary outcomes | Calcium balance/retention; bone metabolism-related molecular and pathway endpoints (e.g., BMP-2, Runx2, OPG/RANKL, osteoclast and osteoblast assays); oxidative stress-related molecular and pathway endpoints (e.g., ROS and NO assays); inflammatory-related molecular and pathway endpoints (e.g., IL-1β, IL-6, MAPK, and NF-κB assays) | Oxidative stress-related molecular and pathway endpoints (e.g., reactive oxygen species (ROS)); levels of inflammation-related factors and pathways (e.g., TNF-α, IL-1β, IL-6, IL-17, MAPK, and NF-κB) | Oxidative stress-related molecular and pathway endpoints (e.g., reactive oxygen species (ROS)); inflammation-related factors and pathway endpoints (e.g., TNF-α, IL-1β, IL-6, PI3K/AKT, and NF-κB) | Apoptosis-related molecules (e.g., Bcl-2 and Caspase-3); oxidative stress-related molecular endpoints (e.g., reactive oxygen species (ROS) detection); levels of inflammation-related factors (e.g., TNF-α, IL-1β, and AKT) |
