# Supplementary material for: Coumarins in musculoskeletal health: a systematic review
Source: Front Nutr. 2026 Sep 9;13:1737767. doi: 10.3389/fnut.2026.1737767 (PMC13597780; doi:10.3389/fnut.2026.1737767)
Supplement: Supplementary file 1 [file Supplementary_file_1.zip › Supplementary Table S1.DOCX]

Supplementary Table 1. The search strategy used in this systematic review.

| **Database** | **Search strategy** |
| --- | --- |
| Web of Science | ((ALL=((Coumarins) OR (linear furan coumarins OR angular furan coumarins OR linear pyranocoumarins OR angular pyranocoumarins OR coumarin OR osthenol OR fraxetin OR demethylsuberosin OR scopoletin OR esculetin OR umbelliferone OR Psoralen OR bergaptol OR Seselin OR xanthyletin)))) AND ALL=((Osteoporosis OR Bone Diseases, Metabolic OR Osteoarthritis OR Cartilage Diseases OR Arthritis, Rheumatoid OR Osteosarcoma) OR (osteoporosis OR bone loss OR decreased bone density OR osteoarthritis OR cartilage injury OR rheumatoid arthritis OR osteosarcoma)) |
| PubMed | ((Coumarins) OR (linear furan coumarins OR angular furan coumarins OR linear pyranocoumarins OR angular pyranocoumarins OR coumarin OR osthenol OR fraxetin OR demethylsuberosin OR scopoletin OR esculetin OR umbelliferone OR Psoralen OR bergaptol OR Seselin OR xanthyletin)) AND ((Osteoporosis OR Bone Diseases, Metabolic OR Osteoarthritis OR Cartilage Diseases OR Arthritis, Rheumatoid OR Osteosarcoma) OR (osteoporosis OR bone loss OR decreased bone density OR osteoarthritis OR cartilage injury OR rheumatoid arthritis OR osteosarcoma)) |
| Embase | (("Coumarins"[MeSH]) OR (linear furan coumarins[tiab] OR angular furan coumarins[tiab] OR linear pyranocoumarins[tiab] OR angular pyranocoumarins[tiab] OR coumarin[tiab] OR osthenol[tiab] OR fraxetin[tiab] OR demethylsuberosin[tiab] OR scopoletin[tiab] OR esculetin[tiab] OR umbelliferone[tiab] OR Psoralen[tiab] OR bergaptol[tiab] OR Seselin[tiab] OR xanthyletin[tiab])) AND (("Osteoporosis"[MeSH] OR "Osteoarthritis"[MeSH] OR "Bone Diseases, Metabolic"[MeSH] OR "Cartilage Diseases"[MeSH] OR "Arthritis, Rheumatoid"[MeSH] OR "Osteosarcoma"[MeSH]) OR (osteoporosis[tiab] OR bone loss[tiab] OR decreased bone density[tiab] OR osteoarthritis[tiab] OR cartilage injury[tiab] OR rheumatoid arthritis[tiab] OR osteosarcoma[tiab])) |
